# Versatile Electrochemical Synthesis of Selenylbenzo[*b*]Furan Derivatives Through the Cyclization of 2-Alkynylphenols

**DOI:** 10.3389/fchem.2022.880099

**Published:** 2022-05-17

**Authors:** Carlos V. Doerner, Marcos R. Scheide, Celso R. Nicoleti, Daniele C. Durigon, Vinícius D. Idiarte, Martinho J. A. Sousa, Samuel R. Mendes, Sumbal Saba, José S. S. Neto, Guilherme M. Martins, Jamal Rafique, Antonio L. Braga

**Affiliations:** ^1^ Departamento de Química, Universidade Federal de Santa Catarina—UFSC, Florianópolis, Brazil; ^2^ Instituto de Química, Universidade Federal do Mato Grosso do Sul.—UFMS, Campo Grande, Brazil; ^3^ Departamento de Química, Universidade do Estado de Santa Catarina, Joinville, Brazil; ^4^ Instituto de Química, Universidade Federal de Goiás—UFG, Goiânia, Brazil; ^5^ Department of Chemical Sciences, Faculty of Science, University of Johannesburg, Doornfontein, South Africa

**Keywords:** selenylbenzo[*b*]furans, seleno-cyclization, electrosynthesis, diselenide, selenium

## Abstract

We report an electrochemical oxidative intramolecular cyclization reaction between 2-alkynylphenol derivatives and different diselenides species to generate a wide variety of substituted-benzo[*b*]furans. Driven by the galvanostatic electrolysis assembled in an undivided cell, it provided efficient transformation into oxidant-, base-, and metal-free conditions in an open system at room temperature. With satisfactory functional group compatibility, the products were obtained in good to excellent yields.

## Introduction

**FIGURE 1:**
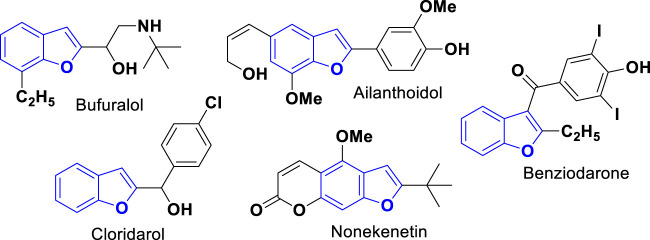
Benzofuran containing some drug molecules.

The benzo[*b*]furan core is present in several derivatives of natural products, containing various types of biological activities (Heravi et al., 2017). Many drugs and candidates for clinical drugs have this nucleus (Miao et al., 2019; Radadiya et al., 2015), such as bufuralol, ailanthoidol, benziodarone, nonekenetin, and cloridarol, as shown in Figure 1 (Asif 2016; Tang et al., 2021). The reported therapeutic activities include antitumor (Romagnoli et al., 2015; Xu et al., 2017), antidepressant (Boukharsa et al., 2016), anti-inflammatory (Xie et al., 2014), antioxidant (Chand et al., 2017), and fungicide (Liang et al., 2016) activities and may also inhibit the formation of amyloid plaques that are characteristic of Alzheimer’s disease (Hiremathad et al., 2018).

Similarly, the construction of the C–Se bond is among the important transformation in organic synthesis (Rafique et al., 2016b; Rafique et al., 2021), mainly due to their properties such as synthetic intermediates in organic transformations (Shao et al., 2019; Arora et al., 2021) and material sciences (Li et al., 2020) as well as in the medicinal chemistry (Nogueria et al., 2021). In the past few decades, these compounds have gained increasing interest, mainly due to their antioxidant (Mugesh and Singh 2000; Botteselle et al., 2021), anti-Alzheimer (Rodrigues et al., 2018; Scheide et al., 2020b; Kumawat et al., 2021), anti-inflammatory (He et al., 2021), antitumor (Spengler et al., 2019; Chen et al., 2020; Dos Santos et al., 2021; Santos et al., 2022), antiviral (Ali et al., 2021), and other biological activities (Wang et al., 2016; Frizon et al., 2020; Rafique et al., 2020; Galant et al., 2021; Martín-Escolano et al., 2021; Veloso et al., 2021).

Considering the biological relevance of benzo[*b*]furans and the wide spectrum of therapeutic properties of organoselenides, there are few synthetic methods that are available to access organo-selenylbenzo[*b*]furans. The most frequent approaches are cyclization reactions using 2-alkynylphenols or 2-alkynyl-alkoxybenzenes. In 2005, Larock and co-workers reported the synthesis of disubstituted benzo[*b*]furans through the cyclization of 2-alkynylanisols in the presence of an electrophilic species of chalcogen (Yue et al., 2005). Zeni and co-workers had developed another approach, which involved cyclization of 2-chalcogenealkynyl anisoles by I_2_, Br_2_, and PhSeBr as electrophilic mediators (Manarin et al., 2009). In the same year, Li and co-workers proposed a palladium-promoted annulation reaction of 2-alkynylphenol derivatives with diselenides or disulfides and iodides (Du et al., 2009). In 2010, Zeni and co-workers also reported a FeCl_3_-diorganyl dichalcogenide-promoted cyclization of 2-alkynylanisoles (Gay et al., 2010). Liu and co-workers proposed the synthesis of 3-selenylbenzo[*b*]furans *via* AgNO_2_-catalyzed radical cyclization of 2-alkynylanisoles or 2-alkynylthioanisoles, elemental Se, and arylboronic acids (An et al., 2019). Zhong and co-workers reported the synthesis of 3-chalcogen-benzo[*b*]furans *via* the I_2_-mediated annulation reaction of 2-alkynylanisoles (Han et al., 2013). Recently, Silva and co-workers reported the synthesis of 3-selenylbenzo[*b*]furans mediated by the Selectfluor^®^ (Xavier et al., 2020). Arsenyan and co-workers developed the synthesis of benzo[*b*]furans and indoles bearing short selenocysteine-containing peptides (Lapcinska et al., 2020), and Xu and co-workers described an electrochemical oxidative cyclization of oximes with diselenides (Gao et al., 2021).

In recent years, organic electrochemistry has emerged as an attractive and suitable approach (Martins et al., 2019a; Martins et al., 2020; Scheide et al., 2021; Huang et al., 2022). Such reactions are economically attractive, requiring only an electric current as a redox medium (Cembellín and Cembellín 2021). In this regard, with the use of electrochemistry, alkyne functionalization in single-stage mode and cyclization have been showing high efficiency, being carried out under milder conditions (Martins et al., 2019b).

Thus, in connection with our continuing interest in designing and developing eco-friendly processes (Godoi et al., 2013; Matzkeit et al., 2018; Peterle et al., 2018; Scheide et al., 2020a; Neto et al., 2020; Saba et al., 2020; Franco et al., 2021) and electrochemical selenylation reactions (Meirinho et al., 2019; Lazzaris et al., 2021; Scheide et al., 2021), we report the synthesis of selenylbenzo[*b*]furan derivatives through an electrochemical oxidative intramolecular cyclization reaction between 2-alkynylphenol derivatives and different diorganyl diselenides. This sustainable approach operates in shorter reaction time, providing the selenylated products in good to excellent yields.

## Results and Discussion

Initially, reaction optimization was performed to determine the optimum reaction conditions; the results are presented in Table 1 (see ESI, S1 for complete optimization table). In entries 1–5, different supporting electrolytes (TBAI, TBAPF_6_, TBABF_4_, LiClO_4_, and TBAClO_4_) were evaluated, in which the most appropriate was tetrabutylammonium perchlorate (TBAClO_4_). The amount of electrolyte was analyzed, varying from 0.4 equiv. to 0.3 equiv., and a slight decrease in yield was observed (entry 6). In entry 7, the equivalence of diphenyl diselenide (2a) has been reduced by 1 equiv. to 0.75 equiv., and a decrease in yield was obtained.

**TABLE 1:** Optimization of the reaction conditions.^a^

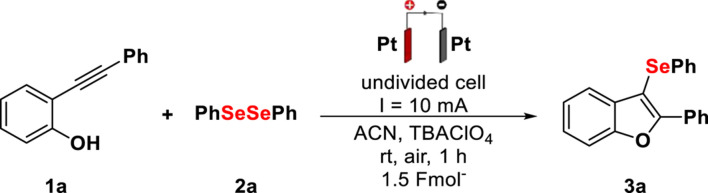		
Entry	Variation from the standard conditions	Yield (%)^b^
1	None	95
2	TBAI instead of TBAClO_4_	-^c^
3	TBAPF_6_ instead of TBAClO_4_	56
4	TBABF_4_ instead of TBAClO_4_	50
5	LiClO_4_ instead of TBAClO_4_	41
6	0.3 equiv. of TBAClO_4_	90
7	0.75 equiv. of **2a**	88
8	C (+) | Pt (-)	82
9	Pt (+) | C (-)	-^c^
10	C (+) | C (-)	-^c^
11	5 mA instead of 10 mA	88
12	15 mA instead of 10 mA	62
13	MeOH as the solvent	-^c^
14	DMSO as the solvent	-^c^

a Reaction conditions: Pt anode, Pt cathode, undivided cell, constant current = 10 mA, **1a** (0.25 mmol), **2a** (0.25 mmol—1.0 equiv.), TBAClO_4_ (0.1 mmol—0.4 equiv.), and ACN (3 ml) at room temperature and under air conditions for 1 h.

b Isolated by column chromatography.

c No reaction.

We emphasize that by applying graphite electrodes, the transformation was not efficient (entries 8–10), obtaining lower yields or no reaction progress. Considering different electrical currents, with 5 mA, a slight reduction in performance was observed (entry 11). Additionally, when the current was increased to 15 mA, a substantial decrease in efficiency was observed (entry 12). Finally, evaluating different solvents, with the application of methanol or dimethyl sulfoxide (entries 13 and 14), in both cases, the reaction did not proceed, and the starting material was completely recovered.

**Scheme 1:**
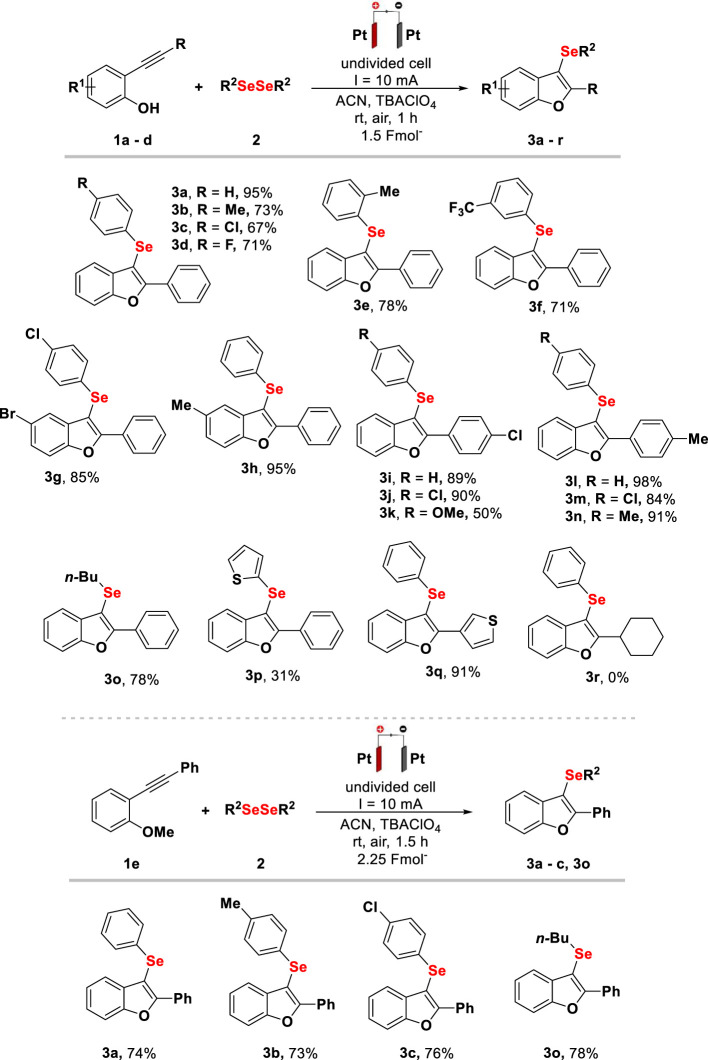
Scope and limitations for electrochemical synthesis of selenylbenzo[*b*]furans (**3**). Reaction conditions: platinum electrodes, constant current (10 mA), **1** (0.25 mmol), **2** (0.25 mmol), TBAClO_4_ (0.1 mmol), ACN (3 ml), rt, and air. Isolated by column chromatography.

Under the optimal reaction conditions in hand, the substrate scope of intramolecular cyclization between 2-alkynylphenol derivatives and different diselenides was evaluated (Scheme 1). Initially, diphenyl diselenides bearing electron-donor and electron-withdrawing groups as well as aliphatic and thiophene diselenides were subjected to transformation, providing the corresponding product yields up to 98%. The reaction proceeded smoothly for diselenides containing the methoxy group, and derivative 3k was obtained with 50% yield. The method showed great compatibility with the electron-withdrawing groups, being suitable for F, Cl, and CF_3_ substituents. Substituent groups in the phenolic ring did not affect the reaction progress, delivering the cyclized products in yields of up to 95% (3 g and 3 h). Aliphatic diselenide was used successfully, providing the product 3o with 78% yield. However, with the thiophene diselenide, the yield decreased, affording the selenylated product 3p with 31% yield. However, for the synthesis of product 3q, with the thiophene heterocycle, the reaction proved to be efficient, delivering the product with 91% yield. It was observed that the transformation is not limited only to cyclizations from 2-alkynylphenols (1a–d), and the use of methoxy-2-(phenylethynyl)benzene (1e) was appropriate, providing products 3a–c and 3o with yields of up to 78%, under the same reaction conditions.

**Scheme 2:**
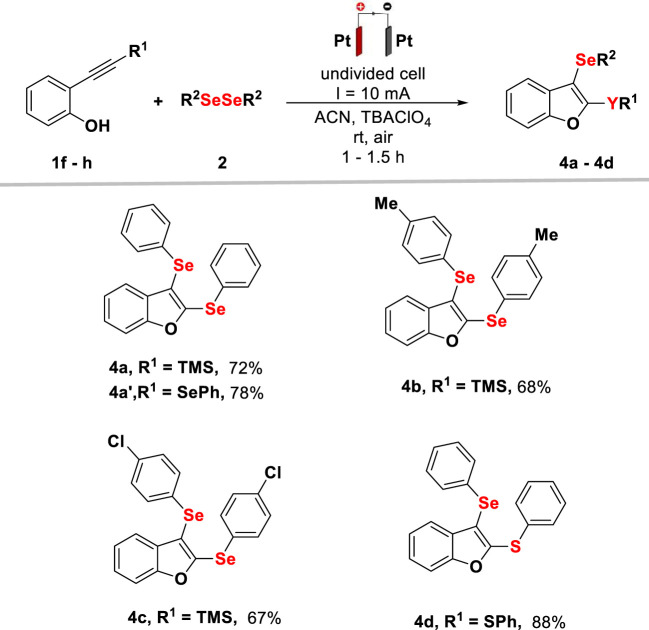
Scope and limitations for electrochemical synthesis of selenylbenzo[*b*]furans (**4**). Reaction conditions: platinum electrodes, constant current (10 mA), **1** (0.25 mmol), **2** (0.35 mmol for R^1^ = TMS, 0.25 mmol for R^1^ = SePh and R^1^ = SPh), TBAClO_4_ (0.1 mmol), ACN (3 ml), rt, and air. Isolated by column chromatography.

In order to expand the reaction scope, the use of 2-[(trimethylsilyl)ethynyl]phenol (**1f**) was evaluated, and to our delight, the bis-selenylation product was observed, as shown in Scheme 2, **4a**. The need of 1.4 equivalent of diselenide for the complete conversion of **1f** into **4a** was observed. For a better understanding, the method was extended to the synthesis of different 2,3-bis-organochalcogenyl-benzo [*b*]chalcogenophenes (**4a**, **4b,** and **4c**), varying the diselenides, reaching yields of up to 72% in 1.5 h. Additionally, the use of 2-[(phenylselanyl)ethynyl]phenol (**1g**) and 2-[(phenylthio)ethynyl]phenol (**1h**) was evaluated, which provided 2,3-bis-organochalcogenyl-benzo [*b*]chalcogenophenes (**4a** ^**’**^ and 4 days) with yields up to 88%, using 1.0 equivalent of diselenides, in 1 h of reaction time.

**Scheme 3:**
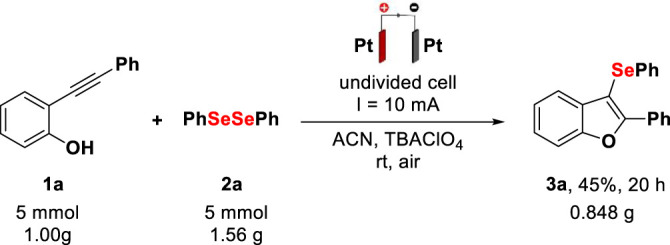
Gram-scale synthesis. Reaction conditions: platinum electrodes, constant current (10 mA), **1a** (5 mmol), **2a** (5 mmol), TBAClO_4_ (2 mmol), ACN (60 ml), rt, and air. Isolated by column chromatography.

To evaluate the applicability of the present method, the electrochemical intramolecular cyclization of 2-(phenylethynyl)phenol **1a** with diphenyl diselenide **2a** was carried out in gram-scale synthesis (5 mmol), affording product **3a** with 45% yield after 20 h; Scheme 3. The cyclic voltammetry of **3a** (ESI S5†) shows an oxidation peak at Epa 1.68 V (vs. NHE), which may be associated with a process of degradation of the selenylated product, resulting in a lower yield for the gram-scale procedure. This suggests that to increase the reaction scale efficiently, it is recommended to enlarge the area of the electrodes, reducing the reaction time.

**Scheme 4:**
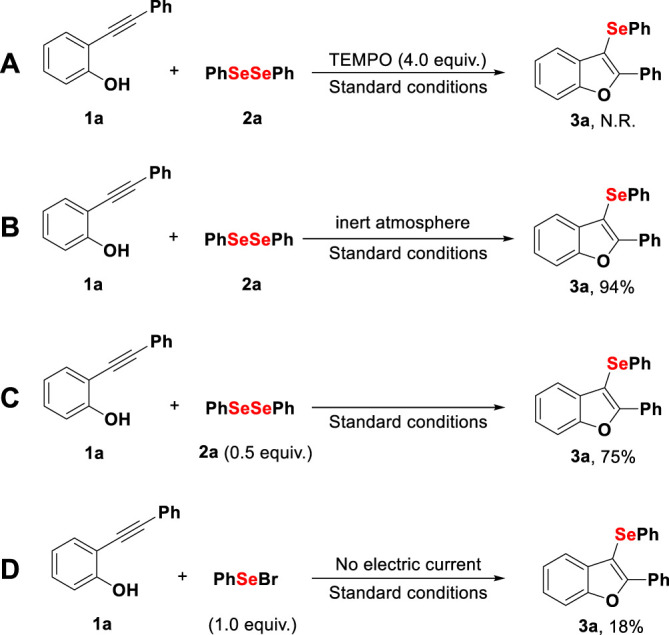
Control experiments. Standard conditions: platinum electrodes, constant current (10 mA), **1a** (0.25 mmol), **2a** (0.25 mmol), TBAClO_4_ (0.1 mmol), ACN (3 ml), 1 h, rt, and air. Isolated by column chromatography. N.R.: no reaction.

For a better understanding of the reaction mechanism, a series of control experiments were performed; Scheme 4. When the radical scavenger TEMPO was used under standard conditions, the reaction was completely inhibited, and no product was observed (A). This observation suggests that a radical is formed in at least one step of the reaction mechanism. The use of an inert atmosphere had no impact on the yield, which implies that atmospheric oxygen does not participate in the reaction mechanism (B). The use of 0.5 equivalent of diphenyl diselenide (**2a**) proved to be inefficient, delivering product **3a** with 75% yield (C). Finally, we applied PhSeBr as a previously synthesized electrophilic source, with the formation of product **3a** with only 18% yield after 1 h, without electric current, which suggests that an electrophilic form of organoselenium may be involved in the mechanism (D).

Normalized cyclic voltammograms of selected compounds are shown in ESI S5†, and they allowed us to obtain more information regarding the redox potentials involved in the catalytic process studied. Diphenyl diselenide **2a** presented an irreversible anodic peak potential (Epa) at 1.55 V in ACN solution that is in line with the study previously reported by Kunai et al (1983), which suggests the formation of radical stages, explaining that the control experiment was carried out, as shown in Scheme 4—A. Recently, Wilken et al (2018) reported that the RSe^+^ species is not the main catalytically active intermediate in redox reactions using aryl diselenides. This statement is in accordance with a control experiment (D), which under standard conditions without the use of electrical energy, delivered product **3a** in a low reaction yield. Additionally, **1a** showed an irreversible Epa at 1.60 V, attributed to the deprotonation of phenol or radical formation in oxygen, suggesting the reaction pathway *via* radical, as previously proposed (Enache et al., 2011).

**Scheme 5:**
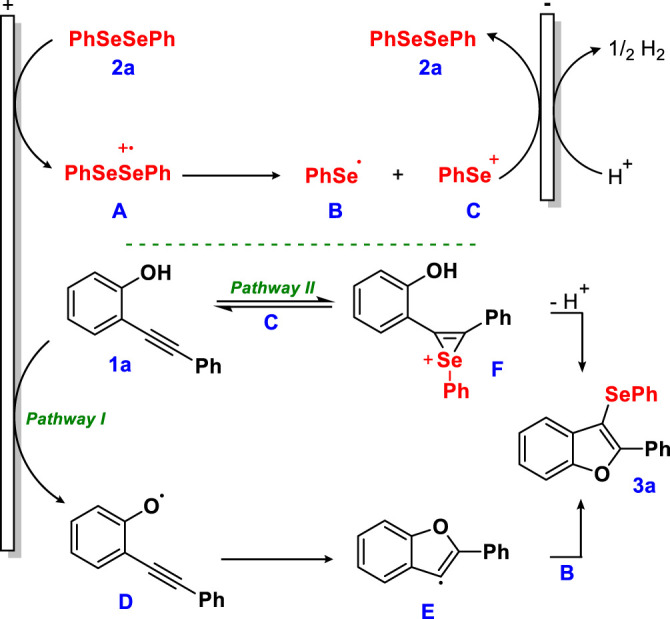
Proposed mechanism for the electrochemical intramolecular cyclization of 2-alkynylphenols with diselenides.

Although the fine details of the reaction mechanism remain unknown, several aspects observed during the control experiments (Scheme 4), normalized cyclic voltammograms (ESI S5†), and previous reports (Azeredo et al., 2014; Manarin et al., 2009; Nascimento et al., 2012; Rafique et al., 2016a; Saba et al., 2015; Saba et al., 2016; Silveira et al., 2012; Xavier et al., 2020) guided us to propose a plausible mechanism (Scheme 5). Considering this, two reactional pathways can be proposed. *Pathway I:* it is known that diphenyl diselenide (**2a**) may be involved in oxidation and reduction processes in the electrocatalytic cycle, suggesting the possibility of the reaction starting with the formation of an intermediate cationic radical **A** *via* anodic oxidation. In parallel, the anodic oxidation of **1a** would promote the radical species **D**, which after addition at the sp carbon forms the intermediate **E**, followed by an addition of **B**, which delivers the desired product **3a**. Moreover, the diselenide can be involved in both processes (oxidation and reduction) under electrochemical conditions, as evidenced in CV (ESI S5†) and in the literature. So, we do not rule out the possibility of the formation of the radical species **B** *via* cathodic reduction, as suggested by Guan et al (2019) and Gao et al (2021). Considering the control reactions, it was observed that the medium was completely inhibited by the addition of 4.0 equiv. of the TEMPO radical scavenger (**entry A**, Scheme 4), indicating that this process possibly occurs *via* a radical pathway. *Pathway II:* however, we cannot rule out the pathway through the phenyl selenium cation **C**. Through the formation of a reversible seleniranium intermediate **F**, followed by nucleophilic intramolecular attack, product **3a** is formed. This pathway was elucidated through control experiments (**entry D**, Scheme 4).

**Scheme 6:**
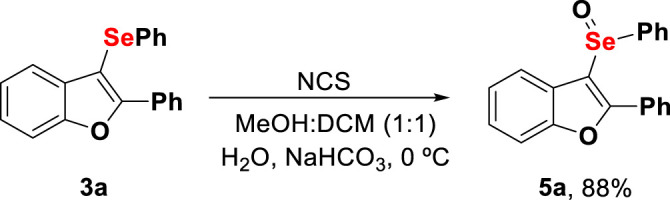
Functionalization of 3-selenylbenzo[*b*]furan derivatives. Reaction conditions: **3a** (0.5 mmol, 1.0 equiv), NCS (1.05 equiv.), 0.07 M in MeOH/CH_2_Cl_2_ (1:1), 0°C, 30 min, NaHCO_3_ (10 ml/1.0 mmol), 0°C, 15 min, and H_2_O (15 ml/1.0 mmol), 0°C, 15 min.

Considering the importance of selenoxide derivatives, we propose the synthesis of 2-phenyl-3-(phenylseleninyl)benzofuran (**5a**) starting from **3a**, as shown in Scheme 6. NCS was applied as an oxidizer (Weilbeer et al., 2016), and the desired selenoxide product **5a** was obtained with 88% yield.

## Conclusion

In summary, we have developed an efficient regioselective electrochemical synthesis of selenylbenzo[*b*]furan derivatives through the cyclization of 2-alkynylphenols. This procedure, driven by the galvanostatic electrolysis using platinum electrodes assembled in an undivided cell, provided efficient transformation into oxidant-free, base-free, and transition metal-free conditions in an open system at room temperature. The method was proved to be robust and can be applied at gram-scale. Additionally, a wide applicability of the present method was observed, being able to be applied in the synthesis of 2,3-bis-organochalcogenyl-benzo[*b*]chalcogenophenes.

## Materials and Methods

### General Information

^1^H and ^13^C NMR spectra were recorded on Bruker 400 and Bruker AC 200 spectrometers, respectively, with the samples dissolved in CDCl_3_. Chemical shifts are reported in ppm downfield from the signal of TMS, used as the internal standard, and the coupling constants (*J*) are expressed in Hertz (Hz). The following abbreviations were reported for multiplicity of signal: s (singlet), d (doublet), t (triplet), q (quartet), quint (quintet), sext (sextet), and m (multiplet). High-resolution mass spectroscopy was record on Xevo G2-S QTOF (Waters) on ESI^+^ and ESI^−^ modes. The reactions were monitored by thin layer chromatography (TLC), and Macherey-Nagel silica gel 818333 of 0.20 mm thickness was used. For visualization, UV fluorescence, an iodine chamber, and acidic methanolic vanillin solution (5% in 10% H_2_SO_4_) were used. An Aldrich technical grade silica gel (pore size 60 Å, 230–400 mesh) was used for flash chromatography. The instruments used for electrochemical studies are BK Precision 1739 V/1A DC power supply with 0.1 mA settable resolution. The anode and cathode platinum plate electrodes (1.0 × 1.0 cm^2^) were used.

### General Procedure of the Electrochemical Setup

To a test tube were added 2-(phenylethynyl)phenol (**1a**, 0.25 mmol), diaryl or dialkyl diselenide (**2**, 0.25 mmol), TBAClO_4_ (0.1 mmol), and 3.0 ml CH_3_CN at room temperature under stirring. The flask was equipped with platinum electrodes (1.0 × 1.0 cm^2^) as the anode and cathode. The reaction mixture was electrolyzed under a constant current mode (10.0 mA). The reaction progress was monitored by TLC. After the total consumption of starting materials, the solvent was removed under reduced pressure to yield a crude mixture from which the final product was isolated through flash column chromatography with a silica gel as the stationary phase and eluated with a mixture of hexane and ethyl acetate.

2-Phenyl-3-(phenylselanyl)benzofuran (**3a**) (Xavier et al., 2020)

White solid (84.1 mg, 95% yield): ^1^H NMR (200 MHz, CDCl_3_) *δ* 8.17 (d, *J* = 7.5 Hz, 2H), 7.46 (d, *J* = 8.0 Hz, 2H), and 7.40–6.94 (m, 10H). ^13^C NMR (50 MHz, CDCl_3_) *δ* 157.4, 154.3, 132.1, 131.6, 130.3, 129.5, 129.4, 128.6, 128.0, 126.4, 125.4, 123.6, 121.5, 111.4, and 100.0.

2-Phenyl-3-(*p*-tolylselanyl)benzofuran (**3b**) (Xavier et al., 2020)

Yellow solid (66.4 mg, 73% yield): ^1^H NMR (400 MHz, CDCl_3_) *δ* 8.32–8.25 (m, 2H), 7.66–7.34 (m, 5H), 7.33–7.22 (m, 3H), 7.04 (d, *J* = 8.0 Hz, 2H), and 2.30 (s, 3H). ^13^C NMR (101 MHz, CDCl_3_) *δ* 157.0, 154.1, 136.2, 132.0, 130.2, 130.1, 129.6, 129.2, 128.5, 127.8, 127.5, 125.2, 123.4, 121.3, 111.1, 100.2, and 21.0.

3-[(4-Chlorophenyl)selanyl]-2-phenylbenzofuran (**3c**) (Xavier et al., 2020). White solid (64.3 mg, 67% yield): ^1^H NMR (400 MHz, CDCl_3_) *δ* 8.26–8.18 (m, 2H), 7.60 (d, *J* = 8.0 Hz, 1H), 7.55–7.35 (m, 5H), and 7.31–7.14 (m, 5H). ^13^C NMR (100 MHz, CDCl_3_) *δ* 157.4, 154.1, 132.3, 131.6, 130.4, 129.9, 129.6, 129.4, 129.4, 128.5, 127.8, 125.4, 123.5, 121.0, 111.3, and 99.4.

3-[(4-Fluorophenyl)selanyl]-2-phenylbenzofuran (**3d**) (An et al., 2019). White solid (65.3 mg, 71% yield): ^1^H NMR (400 MHz, CDCl_3_) *δ* 8.31–8.15 (m, 2H), 7.59 (d, *J* = 8.0 Hz, 1H), 7.55–7.21 (m, 8H), and 6.97–6.83 (m, 2H). *δ* ^13^C NMR (100 MHz, CDCl_3_) δ 161.5 (d, *J*_C-F_ = 246.0 Hz), 157.1, 154.1, 131.7, and 131.4 (d, *J*_C-F_ = 7.5 Hz), 130.1, 129.4, 128.5, 127.8, and 125.6 (d, *J*_C-F_ = 3.0 Hz), 125.3, 123.5, 121.1, and 116.5 (d, *J*_C-F_ = 2.0 Hz), 111.3, and 100.1.

2-Phenyl-3-(*o*-tolylselanyl)benzofuran (**3e**). Yellow solid (70.9 mg, 78% yield): ^1^H NMR (400 MHz, CDCl_3_) *δ* 8.29–8.18 (m, 2H), 7.62 (d, *J* = 8.0 Hz, 1H), 7.56–7.36 (m, 5H), 7.27 (dd, *J* = 8.0, 6.5 Hz, 1H), 7.21 (d, *J* = 7.5 Hz, 1H), 7.10 (td, *J* = 7.5, 1.5 Hz, 1H), 7.04–6.89 (m, 2H), and 2.53 (s, 3H). ^13^C NMR (100 MHz, CDCl_3_) *δ* 157.6, 154.2, 136.7, 132.0, 131.9, 130.2, 130.1, 129.3, 128.5 128.4, 127.8, 126.8, 126.0, 125.2, 123.4, 121.2, 111.2, 99.1, and 21.4. HRMS-ESI [M + H]^+^ calcd. for C_21_H_17_OSe: 365.0445, found 365.0446.

2-Phenyl-3-{[3-(trifluoromethyl)phenyl]selanyl}benzofuran (**3f**) (Xavier et al., 2020). Yellow solid (74.2 mg, 71% yield): ^1^H NMR (400 MHz, CDCl_3_) *δ* 8.26–8.13 (m, 2H), 7.67–7.57 (m, 2H), 7.58–7.33 (m, 6H), and 7.27 (dt, *J* = 12.5, 7.5 Hz, 2H). ^13^C NMR (100 MHz, CDCl_3_) *δ* 157.9, 154.3, 135.0, 132.9, 132.2, 131.8, and 131.6 (2xC), 131.5, 131.4, 130.0, 129.8, 129.8, 129.7, 128.7, 127.9, 125.7, 125.7, 125.7, and 125.6 (2x), 125.1, 125.1, 125.0, 125.0, 125.0, 123.8, 123.2, 123.2, 123.2, 123.1, 122.4, 121.0, 111.5, and 98.9.

5-Bromo-3-[(4-chlorophenyl)selanyl]-2-phenylbenzofuran (**3g**). White solid (98.1 mg, 85% yield): ^1^H NMR (200 MHz, CDCl_3_) *δ* 7.97–7.82 (m, 2H), 7.36–7.35 (m, 1H), 7.26–7.08 (m, 5H), and 7.00–6.78 (m, 4H). ^13^C NMR (50 MHz, CDCl_3_) *δ* 158.6, 152.8, 133.8, 132.6, 130.4, 129.8, 129.5, 129.4, 129.1, 128.5, 128.3, 127.8, 123.6, 116.8, 112.7, and 98.7. HRMS-APCI [M]^+^ calcd. for C_20_H_12_BrClOSe: 461.8925, found 461.8902.

5-Methyl-2-phenyl-3-(phenylselanyl)benzofuran (**3h**) (Gay et al., 2010). Yellow solid (86.5 mg, 95% yield): ^1^H NMR (200 MHz, CDCl_3_) *δ* 8.12 (dd, *J* = 7.5, 2.0 Hz, 2H), 7.59–7.44 (m, 2H), 7.37–7.06 (m, 9H), and 2.38 (s, 3H). ^13^C NMR (50 MHz, CDCl_3_) *δ* 157.6, 154.0, 139.4, 132.04, 131.5, 129.2, 129.1, 127.7, 127.3, 126.1, 125.0, 123.37, 121.0, 111.1, 98.9, and 21.4.

2-(4-Chlorophenyl)-3-(phenylselanyl)benzofuran (**3i**) (Xavier et al., 2020). Yellow solid (85.4 mg, 89% yield): ^1^H NMR (200 MHz, CDCl_3_) δ 8.23–8.09 (m, 2H) and 7.60–7.07 (m, 11H). ^13^C NMR (50 MHz, CDCl_3_) *δ* 155.9, 154.0, 135.2, 131.8, 131.1, 129.3, 129.2, 128.9, 128.7, 128.6, 126.4, 125.5, 123.5, 121.2, 111.1, and 100.2.

2-(4-Chlorophenyl)-3-[(4-chlorophenyl)selanyl]benzofuran (**3j**). White solid (94.0 mg, 90% yield): ^1^H NMR (200 MHz, CDCl_3_) δ 7.88 (d, *J* = 9.0, 2.0 Hz, 2H) and 7.34–6.79 (m, 10H). ^13^C NMR (50 MHz, CDCl_3_) δ 156.0, 154.0, 135.4, 132.5, 131.5, 130.5, 129.5, 129.3, 128.8, 128.4, 125.6, 123.7, 121.0, 111.3, and 99.9. HRMS-APCI [M]^+^ calcd. for C_20_H_12_BrClOSe: 461.8925, found 461.8902.

2-(4-Chlorophenyl)-3-[(4-methoxyphenyl)selanyl]benzofuran (**3k**). White solid (51.6 mg, 50% yield): ^1^H NMR (200 MHz, CDCl_3_) δ 8.19 (d, *J* = 9.0 Hz, 2H), 7.56–7.17 (m, 8H), 6.72 (d, *J* = 9.0 Hz, 2H), and 3.71 (s, 3H). ^13^C NMR (50 MHz, CDCl_3_) δ 158.9, 155.2, 153.9, 135.1, 131.9, 128.8, 128.6, 125.3, 123.4, 121.2, 120.7, 115.1, 111.1, 101.5, and 55.2. HRMS-ESI [M + OH]^+^ calcd. for C_21_H_16_ClO_3_Se: 430.9953, found 430.9798.

3-(Phenylselanyl)-2-(*p*-tolyl)benzofuran (**3L**) (Xavier et al., 2020). White solid (87.0 mg, 98%): ^1^H NMR (200 MHz, CDCl_3_) δ 8.10 (d, *J* = 8.2 Hz, 2H), 7.51 (t, *J* = 7.6 Hz, 2H), 7.37–7.00 (m, 9H), and 2.38 (s, 3H). ^13^C NMR (50 MHz, CDCl_3_) δ 157.6, 154.1, 139.5, 132.0, 131.6, 129.2, 129.1, 127.7, 127.3, 126.2, 124.9, 123.4, 121.1, 111.1, 98.8, and 21.4.

3-[(4-Chlorophenyl)selanyl]-2-(*p*-tolyl)benzofuran (**3m**). Yellow solid (84.5 mg, 84%): ^1^H NMR (200 MHz, CDCl_3_) δ 8.10 (d, *J* = 8.0 Hz, 2H), 7.51 (t, *J* = 7.5 Hz, 2H), 7.37–6.95 (m, 8H), and 2.38 (s, 3H). ^13^C NMR (50 MHz, CDCl_3_) δ 157.6, 154.0, 139.5, 132.0, 131.6, 129.2, 127.7, 127.3, 126.2, 124.9, 123.4, 121.1, 111.1, 98.9, and 21.4. HRMS-ESI [M + OH]^+^ calcd. for C_21_H_16_ClO_2_Se: 415.0004, found 414.9989.

2-(p-Tolyl)-3-(*p*-tolylselanyl)benzofuran (**3n**). White solid (85.9 mg, 91%): ^1^H NMR (200 MHz, CDCl_3_) δ 8.10 (d, *J* = 8.0 Hz, 2H), 7.51 (t, *J* = 6.0 Hz, 2H), 7.40–7.09 (m, 6H), 6.96 (d, *J* = 8.0 Hz, 2H), 2.39 (s, 3H), and 2.23 (s, 3H). ^13^C NMR (50 MHz, CDCl_3_) *δ* 157.3, 154.0, 139.4, 136.1, 132.1, 130.1, 129.5, 129.2, 127.7, 127.4, 124.9, 123.3, 121.1, 111.0, 99.4, 21.4, and 20.9. EIMS (*m/z,* rel. int. %) 298 (100), 178 (14), 255 (11), and 378 (28). HRMS not ionized in ESI and APCI.

3-(Butylselanyl)-2-phenylbenzofuran (**3o**) (Xavier et al., 2020). Yellow oil (64.6 mg, 78% yield): ^1^H NMR (400 MHz, CDCl_3_) *δ* 8.39–8.29 (m, 2H), 7.71 (m, 1H), 7.58–7.27 (m, 6H), 2.82 (t, *J* = 7.5 Hz, 2H), 1.66–1.53 (m, 2H), 1.42–1.30 (m, 2H), and 0.82 (t, *J* = 7.5 Hz, 3H). ^13^C NMR (100 MHz, CDCl_3_) δ 155.9, 153.9, 132.7, 130.7, 128.9, 128.4, 127.7, 124.9, 123.1, 121.0, 111.1, 100.4, 32.4, 28.3, 22.7, and 13.5.

2-Phenyl-3-(thiophen-2-ylselanyl)benzofuran (**3p**). Yellow solid (27.6 mg, 31% yield): ^1^H NMR (400 MHz, CDCl_3_) *δ* 8.39–8.19 (m, 2H), 7.77–7.66 (m, 1H), 7.60–7.20 (m, 8H), and 6.98–6.85 (m, 1H). ^13^C NMR (100 MHz, CDCl_3_) *δ* 156.2, 153.9, 133.6, 131.7, 130.2, 129.9, 129.3, 128.5, 128.0, 127.8, 125.2, 123.4, 121.0, 111.2, and 102.1. EIMS (*m/z,* rel. int. %) 276 (100), 44 (10), 165 (22), and 356 (21). HRMS not ionized in ESI and APCI.

3-(Phenylselanyl)-2-(thiophen-3-yl)benzofuran (**3q**). Pale yellow solid (80.8 mg, 91% yield): ^1^H NMR (200 MHz, CDCl_3_) *δ* 8.23–8.06 (m, 1H), 7.95 (d, *J* = 5.0 Hz, 1H), 7.50 (d, *J* = 8.0 Hz, 2H), and 7.41–6.92 (m, 9H). ^13^C NMR (50 MHz, CDCl_3_) *δ* 154.7, 154.0, 131.9, and 131.4 (2xC), 129.4, 129.3, 126.8, 126.4, 126.0, 125.4, 125.2, 123.6, 121.1, 111.2, and 98.9. HRMS-ESI [M + OH]^+^ calcd. for C_18_H_13_O_2_SSe: 372.9801, found 372.9798.

2,3-Bis(phenylselanyl)benzofuran (**4a, 4a’**) (Perin et al., 2019). Yellow solid (77.2 mg, 72% yield): ^1^H NMR (400 MHz, CDCl_3_) *δ* 7.58–7.52 (m, 2H), 7.49 (d, *J* = 8.0 Hz, 1H), 7.44 (dd, *J* = 8.0, 1.4 Hz, 1H), and 7.37–7.16 (m, 10H). ^13^C NMR (100 MHz, CDCl_3_) *δ* 157.3, 150.8, 132.8, 130.7, 130.5, 129.4, 129.2, 128.9, 128.9, 127.9, 126.7, 125.3, 123.5, 121.0, 113.7, and 111.4.

2,3-Bis(*p*-tolylselanyl)benzofuran (**4b**). Yellow solid (77.6 mg, 68% yield): ^1^H NMR (400 MHz, CDCl_3_) δ 7.50–7.40 (m, 4H), 7.32–7.18 (m, 4H), 7.09 (d, *J* = 8.0 Hz, 2H), 7.00 (d, *J* = 8.0 Hz, 2H), 2.34 (s, 3H), and 2.29 (s, 3H). ^13^C NMR (100 MHz, CDCl_3_) *δ* 157.1, 138.1, 136.7, 133.3, 130.9, 130.5, 130.2, 130.0, 126.8, 125.1, 123.4, 120.9, 113.4, 111.3, 21.2, and 21.0. HRMS-APCI [M + H]^+^ calcd. for C_22_H_19_OSe_2_: 458,9766, found 458.9756.

2,3-Bis[(4-chlorophenyl)selanyl]benzofuran (**4c**). Yellow solid (83.1 mg, 67% yield): ^1^H NMR (400 MHz, CDCl_3_) *δ* 7.50 (d, *J* = 8.3 Hz, 1H), 7.47–7.41 (m, 2H), 7.34 (td, *J* = 8.3, 7.2, 1.4 Hz, 1H), 7.29–7.18 (m, 4H), and 7.17–7.10 (m, 1H). ^13^C NMR (100 MHz, CDCl_3_) *δ* 157.3, 134.6, 134.4, 133.1, 131.8, 131.2, 130.2, 129.7, 129.5, 128.9, 126.9, 125.8, 123.9, 120.9, 113.5, and 111.6. HRMS-APCI [M + OH]^+^ calcd. for C_20_H_13_Cl_2_O_2_Se_2_:514.8602, found 514.8602.

3-(Phenylselanyl)-2-(phenylthio)benzofuran (**4d**). Yellow solid (83.7 mg, 88% yield): ^1^H NMR (200 MHz, CDCl_3_) *δ* 7.45–7.37 (m, 2H) and 7.36–7.11 (m, 12H). ^13^C NMR (50 MHz, CDCl_3_) *δ* 156.4, 152.9, 133.4, 131.0, 130.5, 130.3, 129.3, 127.6, 126.90, 125.9, 123.6, 121.3, 112.8, and 111.6.

2-[(Phenylselanyl)ethynyl]phenol (**1g**). Brown solid (90.6 mg, 33% yield): ^1^H NMR (400 MHz, CDCl_3_) *δ* 7.60–7.54 (m, 1H), 7.40 (dd, *J* = 7.5, 1.5 Hz, 1H), 7.36–7.24 (m, 4H), 7.01–6.93 (m, 1H), 6.89 (td, *J* = 7.5, 1.0 Hz, 1H), and 5.91 (s, 1H). ^13^C NMR (100 MHz, CDCl_3_) *δ* 157.7, 132.5, 131.3, 129.9, 129.4, 128.6, 127.6, 120.5, 115.0, 109.7, 96.7, and 77.2. HRMS-ESI [M + H]^+^ calcd. for C_14_H_11_OSe: 274.9975, found 274.9988.

2-((Phenylthio)ethynyl)phenol (**1h**). Yellow solid (236.2 mg, 54% yield): ^1^H NMR (200 MHz, CDCl_3_) *δ* 7.58–7.13 (m, 7H), 6.92 (dd, *J* = 18.5, 8.0 Hz, 2H), and 5.90 (s, 1H). ^13^C NMR (50 MHz, CDCl_3_) *δ* 157.8, 132.9, 132.5, 131.5, 129.6, 127.0, 126.6, 120.6, 115.1, 109.4, 91.6, and 83.4. HRMS-ESI [M-H]^-^ calcd. for C_14_H_9_OS: 225.0374, found 225.0370.

2-Phenyl-3-(phenylseleninyl)benzofuran (**5a**). White solid (160.7 mg, 88% yield): ^1^H NMR (400 MHz, CDCl_3_) *δ* 7.97–7.91 (m, 2H), 7.82 (dd, *J* = 7.5, 2.0 Hz, 2H), 7.59–7.42 (m, 8H), 7.28 (d, *J* = 14.5 Hz, 1H), and 7.14–7.06 (m, 1H). ^13^C NMR (100 MHz, CDCl_3_) *δ* 158.6, 154.4, 140.1, 131.3, 130.8, 129.8, 129.3, 128.8, 128.4, 126.8, 126.5, 125.7, 124.0, 121.4, 115.1, and 111.6. HRMS-ESI [M + H]^+^ calcd. for C_20_H_15_O_2_Se: 367.0237, found 367.0235.

### General Procedure for Cyclic Voltammetry

Cyclic voltammograms were obtained using a BAS Epsilon potentiostat/galvanostat. All electrochemical measurements were obtained in acetonitrile solution containing 0.1 molL^-1^ of TBAClO_4_ as the supporting electrolyte under an argon atmosphere. The electrochemical cell employed had a three-electrode configuration: platinum (working), platinum wire (counter), and Ag/Ag^+^ (reference). The Fc^+^/Fc couple was used as an internal standard (E_1/2_ = 400 mV vs. NHE).

## Data Availability

The original contributions presented in the study are included in the article/Supplementary Material,, further inquiries can be directed to the corresponding authors.
