# Supplementary material for: Versatile Electrochemical Synthesis of Selenylbenzo[b]Furan Derivatives Through the Cyclization of 2-Alkynylphenols
Source: Front Chem. 2022 May 17;10:880099. doi: 10.3389/fchem.2022.880099 (PMC9152116; doi:10.3389/fchem.2022.880099)
Supplement: Supplementary file 1 [file DataSheet1.docx]

## Supplementary Material

Versatile Electrochemical Synthesis of Selenylbenzo[*b*]furans Derivatives Through the Cyclization of 2-alkynylphenols

Carlos V. Doerner^1^, Marcos R. Scheide^1^, Celso R. Nicoleti^1^, Daniele C. Durigon^1^, Vinícius D. Idiarte^1^, Martinho J. A. Sousa^2^, Samuel R. Mendes^3^, Sumbal Saba^4^, José S. S. Neto^1^, Guilherme M. Martins^1*^, Jamal Rafique^2,4*^, Antonio L. Braga^1,5*^

^1^Departamento de Química, Universidade Federal de Santa Catarina – UFSC, Florianópolis, 88040-900 SC, Brazil

^2^Instituto de Química, Universidade Federal do Mato Grosso do Sul – UFMS, Campo Grande, 79074-460 MS, Brazil

^3^Departamento de Química, Universidade do Estado de Santa Catarina, Joinville, 89219-719 SC, Brazil

^4^Instituto de Química, Universidade Federal de Goiás – UFG, Goiânia, GO 74690-900, Brazil.

^5^Department of Chemical Sciences, Faculty of Science, University of Johannesburg, Doornfontein, 2028 South Africa

*** Correspondence:**Guilherme M. Martins

[guilherme.m.martins85@gmail.com](mailto:guilherme.m.martins85@gmail.com)

Jamal Rafique
[jamal.chm@gmail.com](mailto:email@uni.edu) ; [jamal.rafique@ufms.br](mailto:jamal.rafique@ufms.br)

Antonio L. Braga
[braga.antonio@ufsc.br](mailto:braga.antonio@ufsc.br)

**Table of Contents**

**1. Complete Optimization Table S2**

**2. General information S3**

**3. General Procedures S3**

**4. Gram-Scale Synthesis Procedure S4**

**5. General Procedure for Cyclic Voltammetry S5**

**6 Products Characterization Data S6**

**7. NMR Spectra S12**

**8. References S36Table S1. Complete Optimization Table.**

|  | | |
| --- | --- | --- |
| **Entry** | **Variation from the standard conditions** | **Yield (%)^b^** |
| 1 | None^a^ | 95 |
| 2 | KI instead of TBAClO_4_ | -^c^ |
| 3 | TBAI instead of TBAClO_4_ | -^c^ |
| 4 | TBABr instead of TBAClO_4_ | -^c^ |
| 5 | TBAPF_6_ instead of TBAClO_4_ | 56 |
| 6 | TBABF_4_ instead of TBAClO_4_ | 50 |
| 7 | LiClO_4_ instead of TBAClO_4_ | 41 |
| 8 | 0.1 equiv. of TBAClO4 | 84 |
| 9 | 0.2 equiv. of TBAClO4 | 88 |
| 10 | 0.3 equiv. of TBAClO_4_ | 90 |
| 11 | 0.5 equiv. of **2a** | 75 |
| 12 | 0.75 equiv. of **2a** | 88 |
| 13 | C (+) \| Pt (-) | 82 |
| 14 | Pt (+) \| C (-) | -^c^ |
| 15 | C (+) \| C (-) | -^c^ |
| 16 | 5 mA instead 10 mA | 88 |
| 17 | 15 mA instead 10 mA | 62 |
| 18 | MeOH as the solvent | -^c^ |
| 19 | DMSO as the solvent | -^c^ |
| 20 | H_2_O as the solvent | -^c^ |

^a^ Reaction conditions: Pt anode, Pt cathode, undivided cell, constant current = 10 mA, **1a** (0.25 mmol), **2a** (0.25 mmol - 1.0 equiv.) and TBAClO_4_ (0.1 mmol - 0.4 equiv.), ACN (3 mL) at room temperature and under air conditions for 1 h. ^b^ Isolated by column chromatography. ^c^ No reaction.

**1. General information**

^1^H and ^13^C NMR spectra were recorded on Bruker 400 and Bruker AC 200 spectrometer, with the samples dissolved in CDCl_3_. Chemical shifts are reported in in ppm downfield from the signal of TMS, used as internal standard, and the coupling constants (*J*) are expressed in Hertz (Hz). Following abbreviations were reported for multiplicity of signal: s (singlet), d (doublet), t (triplet), q (quartet), quint (quintet), sext (sextet) and m (multiplet). Low resolution mass spectra were obtained from a Shimadzu Nexis GC-2030 gas chromatograph with SH-Rxi-1ms (100% polysiloxane), 30 m, 0.25 mmID, 0.25 um df, coupled to CGMS-QP2020 NX mass spectrometer. High resolution mass spectroscopy was record on Xevo G2-S QTOF (Waters) on ESI^+^ and ESI^-^ mode. The reactions were monitored by thin layer chromatography (TLC), Macherey-Nagel Silica Gel 818333, 0.20 mm thickness were used. For visualization, UV fluorescence, iodine chamber and acidic methanolic vanillin solution (5% in 10% H_2_SO_4_). Aldrich technical grade silica gel (pore size 60 Å, 230–400 mesh) was used for flash chromatography. The instruments for electrochemical studies are BK Precision 1739 V/ 1A DC Power supply with 0.1 mA settable resolution. The anode and cathode platinum plate electrode (1.0×1.0 cm^2^).

**2. General Procedures**

**2.1. Synthesis of starting materials**

The 2-alkynylphenols, methoxy-2-(phenylethynyl)benzene 2-[(trimethylsilyl)ethynyl]phenol and 2-ethynylphenol were synthesized following the literature procedures^1,2^ The 2-((phenylselanyl)ethynyl)phenol (**1g**) and 2-((phenylthio)ethynyl)phenol (**1h**) were synthesized following literature procedures with small modifications.^3,4^

**2.1.1. Synthesis of 2-((phenylselanyl)ethynyl)phenol (1g).**

In a Schlenk tube, flame-dried, argon atmosphere under stirring were added the 2-ethynylphenol (0.118g, 1.0 mmol) in 1.0 mL of dried THF. The reaction vessel was cooled at -78 ºC and *n-*BuLi (1.43 mL of a 1.4 M solution in hexane, 2.0 mmol) were added dropwise. After the addition, it was allowed to react for 30 min. to form the corresponding lithium acetylide. Then PhSeBr (0.235 g, 1.0 mmol) were dissolved in 1.0 mL of dried THF and added dropwise to the reaction mixture. After the completing the addition, the reaction contend was allowed to reach the room temperature, it was left to react for another two hours. Compound **1g** was purified using 1:10 mixture of ethyl acetate/hexane as eluent.

**2.1.2. Synthesis of 2-((phenylselanyl)ethynyl)phenol (1g).**

In a Schlenk tube (A), flame-dried, argon atmosphere under stirring were added the 2-ethynylphenol (0,118 g, 1.0 mmol) in 3.0 mL of dried THF. The reaction vessel was cooled at -78 ºC and *n-*BuLi (1.43 mL of a 1.4M solution in hexane, 2.0 mmol) were added dropwise. After the addition, was allowed to react for 30 min to form the corresponding lithium acetylide. Then PhSSPh (0.120 g, 0.55 equiv.) were dissolved in 5.0 mL of dried THF in Schlenk tube (B), flame-dried under argon atmosphere. In the Schlenk B were added Br_2_ (86.8 mg, 0.55 equiv.) and let react for 30 min. The reaction contend of Schlenk tube B was poured with cannula over the lithium acetylide formed in Schlenk tube A. After the addition was allowed to reach room temperature and react for additional 3 hours. Then, the reaction contend was quenched with 10 mL saturated solution of NH_4_Cl followed of extraction with ethyl acetate, dried over MgSO_4_ and concentrated under reduced pressure. The crude product was purified by column chromatography using 1:10 mixture of ethyl acetate/hexane as eluent, to give **1h**.

**2.2. General procedure of electrochemical setup.**

To a test tube were added the 2-(phenylethynyl)phenol (**1a**, 0.25 mmol), diaryl or dialkyl diselenide (**2**, 0.25 mmol), TBAClO_4_ (0.1 mmol) and 3.0 mL CH_3_CN at room temperature under stirring. The flask was equipped with platinum electrodes (1.0×1.0 cm^2^) as the anode and as the cathode. The reaction mixture was electrolyzed under constant current mode (10.0 mA). The reaction progress was monitored by TLC. After the total consumption of starting materials, the solvent was removed under reduced pressure to yield a crude mixture from which the final product was isolated through flash column chromatography with silica gel as stationary phase and eluate with mixture of hexane and ethyl acetate.

**3.** **Gram-Scale Synthesis Procedure**

In a becker (100 mL) were added the 2-(phenylethynyl)phenol (**1a**, 5.0 mmol), diphenyl diselenide (**2a**, 5.0 mmol), TBAClO_4_ (0.2 mmol) and 60 mL CH_3_CN under stirring at room temperature. The becker was equipped with platinum electrodes (1.0×1.0 cm^2^) as the anode and as the cathode. The reaction mixture was electrolyzed under constant current mode (10.0 mA). The reaction time was monitored by the disappearance of the starting material by TLC, taking 20 hours to complete. Later, the solvent was removed under reduced pressure and **3a** was isolated by column chromatography in silica gel and a mixture of hexane and ethyl acetate as eluent.

**5.** **General Procedure for Cyclic Voltammetry**

Cyclic voltammograms were obtained using a BAS Epsilon potentiostat/galvanostat. All electrochemical measurements were obtained in acetonitrile solution containing 0.1 mol L^-1^ of TBAPF_6_ as the supporting electrolyte, under argon atmosphere. The electrochemical cell employed had three-electrode configuration: platinum (working), platinum wire (counter) and Ag/Ag^+^ (reference). The Fc^+^/Fc couple was used as an internal standard ( E_1/2_= 400 mV vs NHE).


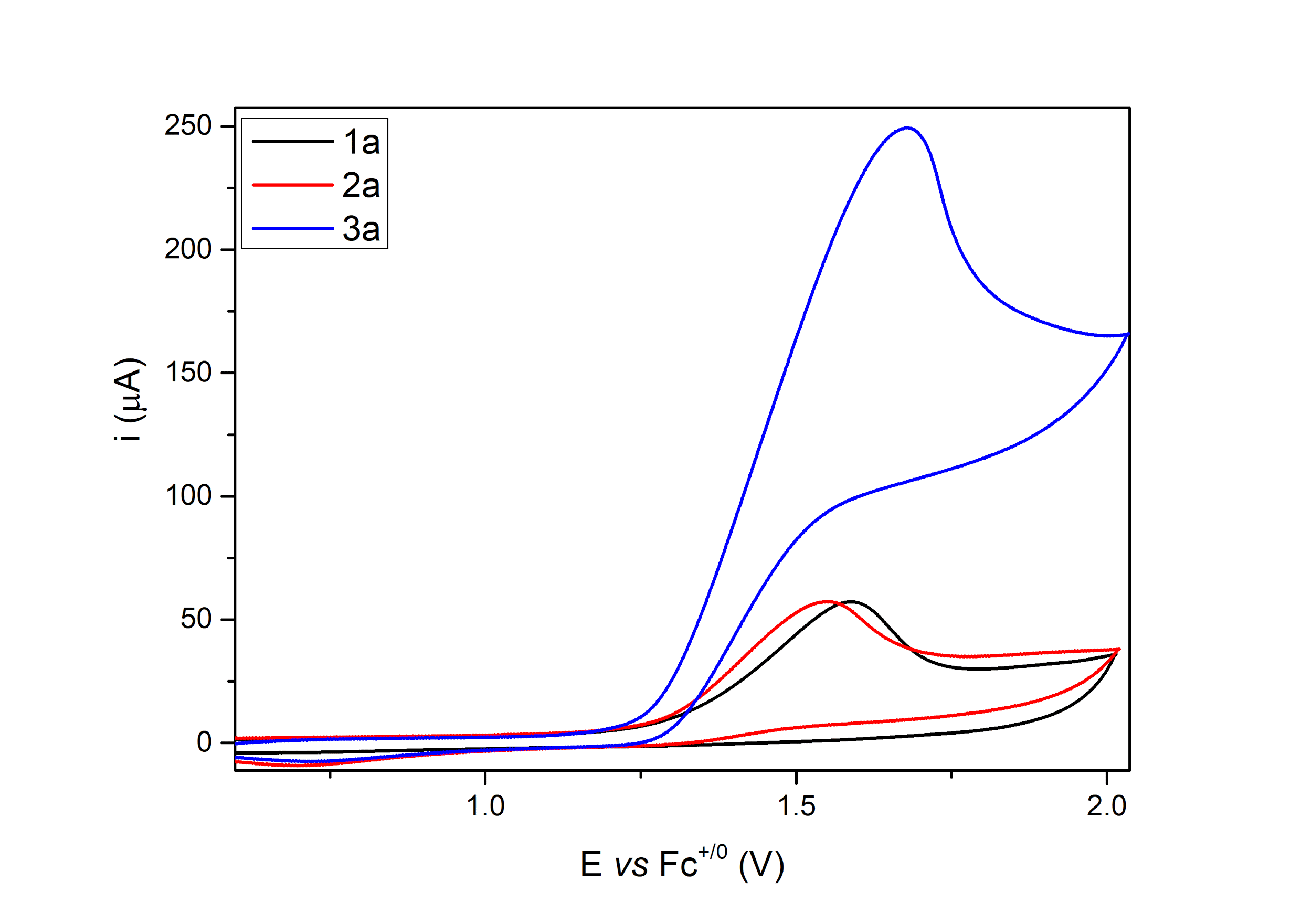


**Figure S1.** Cyclic voltammetry of **1a**, diphenyl diselenide **2a** and **3a** in ACN. Working electrode: platinum; counter electrode: platinum; reference electrode: Ag/Ag^+^; electrolyte: TBAPF_6_ (0.1 molL^-1^); scan rate: 100 mV s^-1^.


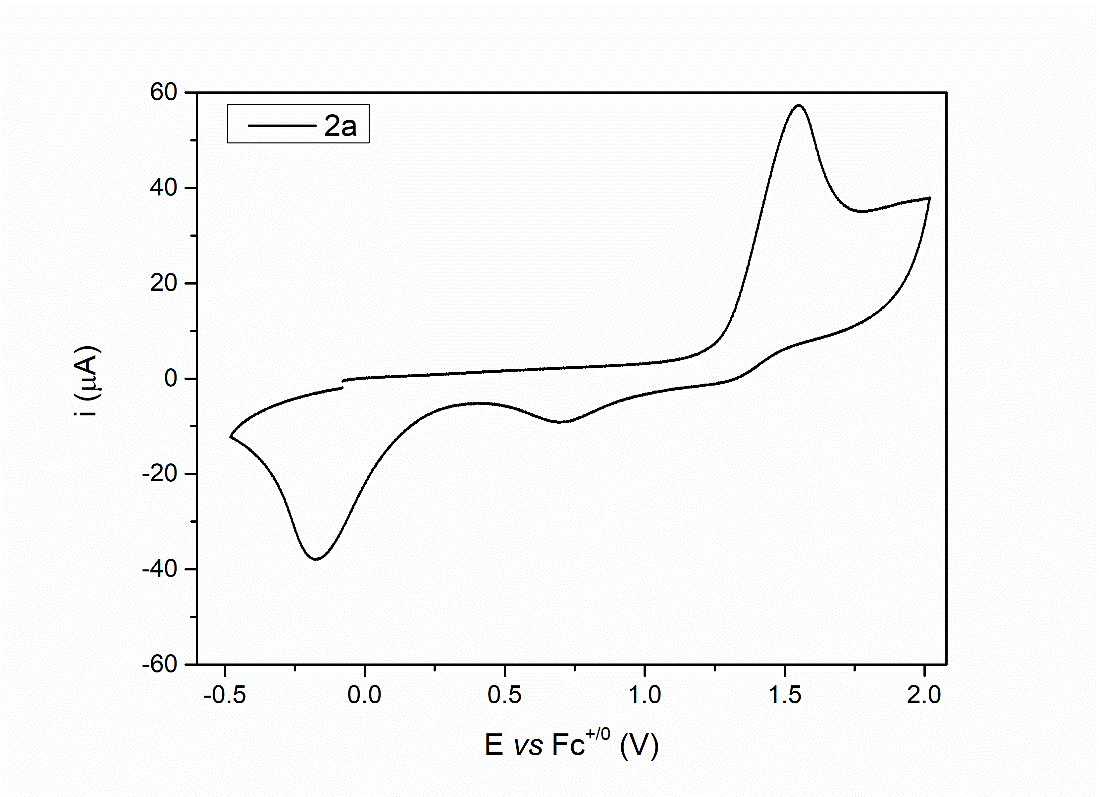


**Figure S2.** Cyclic voltammetry of diphenyl diselenide **2a** in CH_3_CN. Working electrode: platinum; counter electrode: platinum; reference electrode: Ag/Ag^+^; electrolyte: TBAClO_4_ (0.1 mol L^-1^); scan rate: 100 mV s^-1^.

**6. Products Characterization Data**


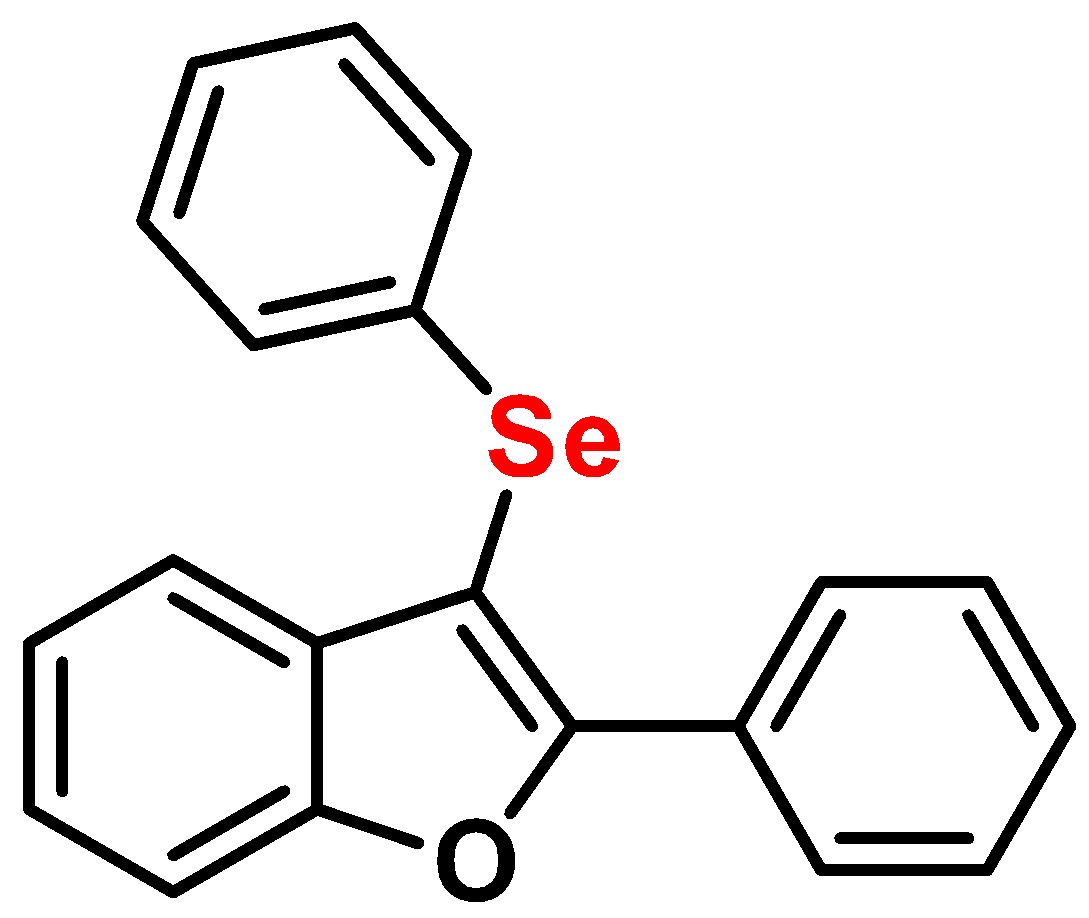
2-phenyl-3-(phenylselanyl)benzofuran (**3a**)^5^

White Solid (84.1 mg, 95% yield): ^1^H NMR (200 MHz, CDCl_3_) *δ* 8.17 (d, *J* = 7.5 Hz, 2H), 7.46 (d, *J* = 8.0 Hz, 2H), 7.40 – 6.94 (m, 10H). ^13^C NMR (50 MHz, CDCl_3_) *δ* 157.4, 154.3, 132.1, 131.6, 130.3, 129.5, 129.4, 128.6, 128.0, 126.4, 125.4, 123.6, 121.5, 111.4, 100.0.


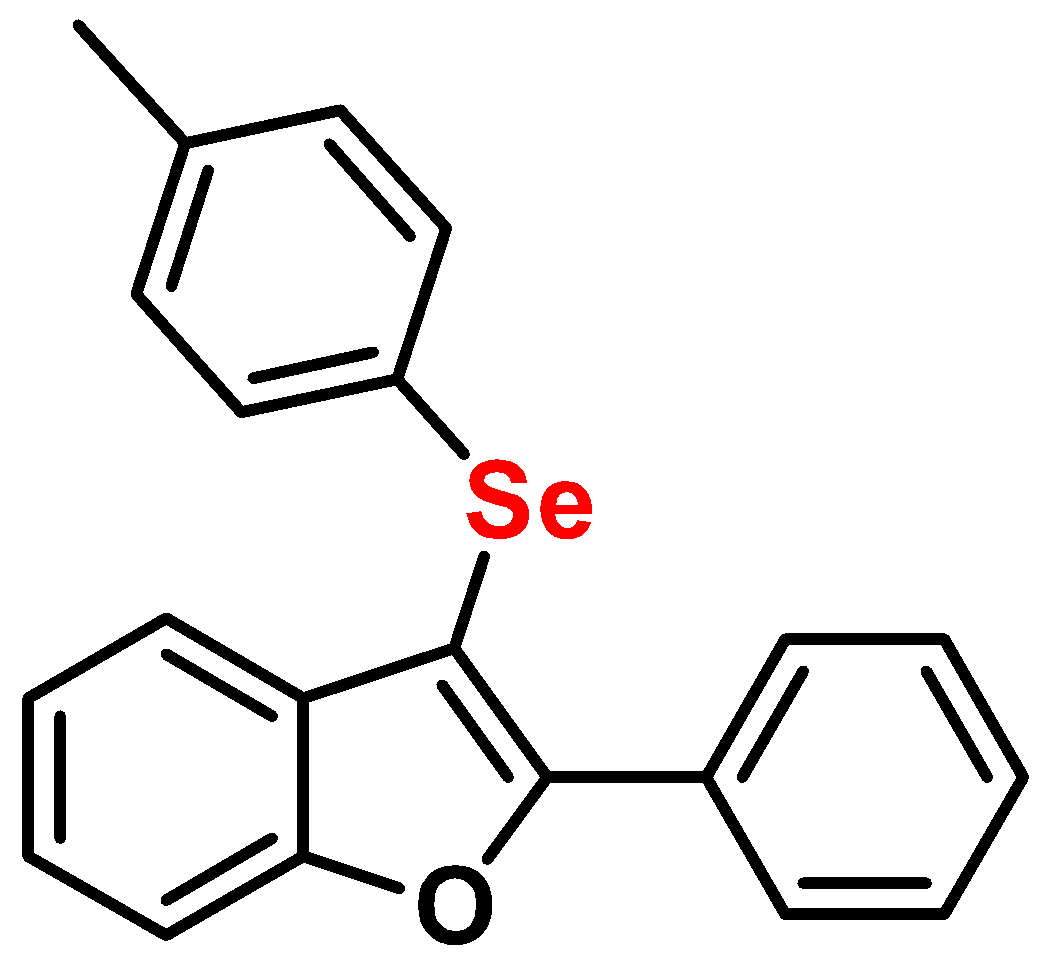
2-phenyl-3-(p-tolylselanyl)benzofuran (**3b**)^5^

Yellow Solid (66.4 mg, 73% yield): ^1^H NMR (400 MHz, CDCl_3_) *δ* 8.32 – 8.25 (m, 2H), 7.66 – 7.34 (m, 5H), 7.33 – 7.22 (m, 3H), 7.04 (d, *J* = 8.0 Hz, 2H), 2.30 (s, 3H). ^13^C NMR (101 MHz, CDCl_3_) *δ* 157.0, 154.1, 136.2, 132.0, 130.2, 130.1, 129.6, 129.2, 128.5, 127.8, 127.5, 125.2, 123.4, 121.3, 111.1, 100.2, 21.0.


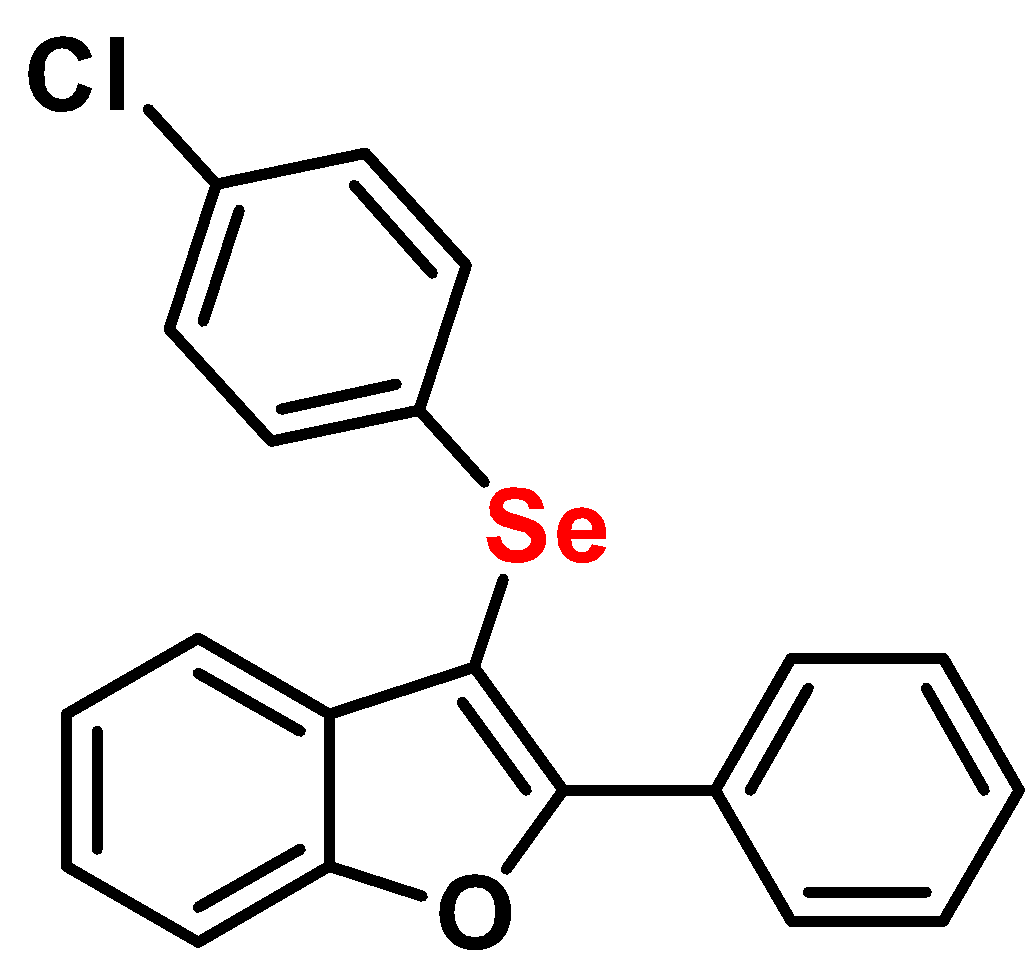
3-((4-chlorophenyl)selanyl)-2-phenylbenzofuran (**3c**)^5^

White Solid (64.3 mg, 67% yield): ^1^H NMR (400 MHz, CDCl_3_) *δ* 8.26 – 8.18 (m, 2H), 7.60 (d, *J* = 8.0 Hz, 1H), 7.55 – 7.35 (m, 5H), 7.31 – 7.14 (m, 5H). ^13^C NMR (100 MHz, CDCl_3_) *δ* 157.4, 154.1, 132.3, 131.6, 130.4, 129.9, 129.6, 129.4, 129.4, 128.5, 127.8, 125.4, 123.5, 121.0, 111.3, 99.4.


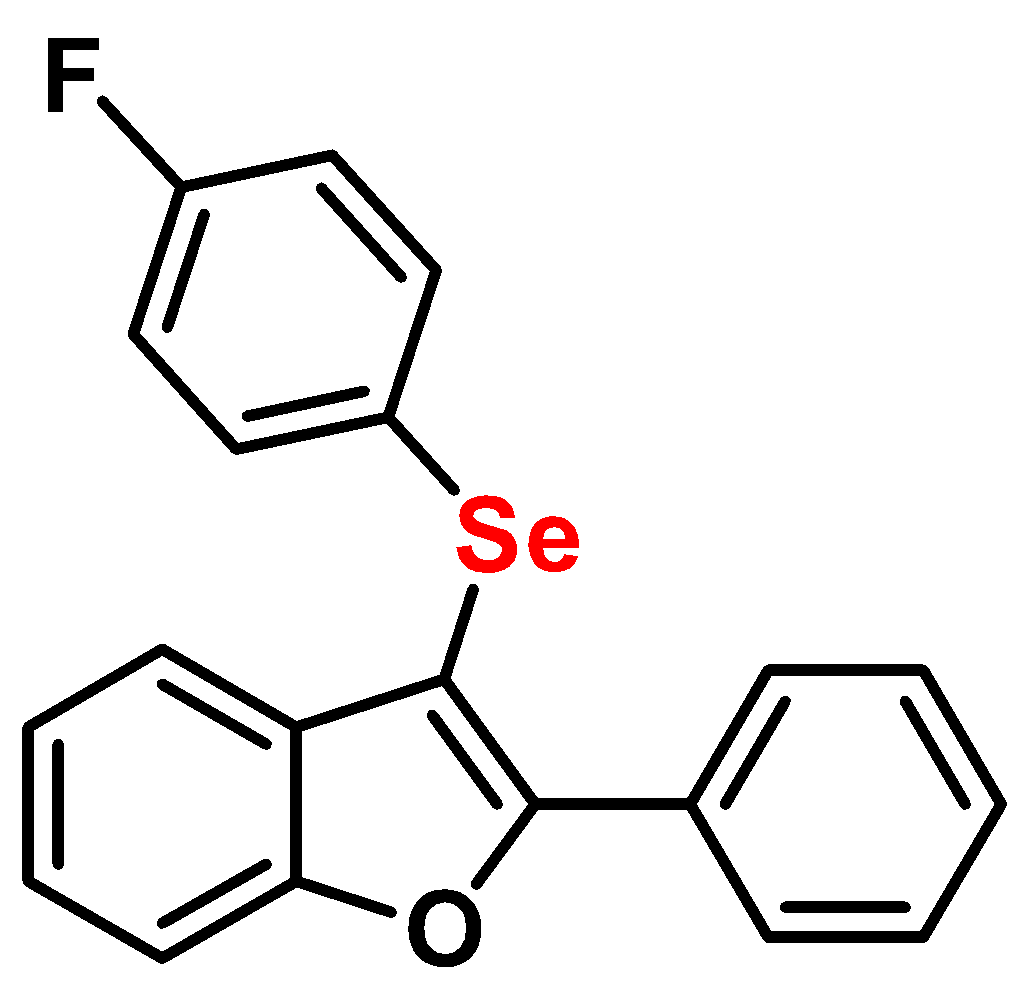
3-((4-fluorophenyl)selanyl)-2-phenylbenzofuran (**3d**)^6^

White Solid (65.3 mg, 71% yield): ^1^H NMR (400 MHz, CDCl_3_) *δ* 8.31 – 8.15 (m, 2H), 7.59 (d, *J* = 8.0 Hz, 1H), 7.55 – 7.21 (m, 8H), 6.97 – 6.83 (m, 2H). *δ* ^13^C NMR (100 MHz, CDCl_3_) δ 161.5 (d, *J*_C-F_ = 246.0 Hz) , 157.1, 154.1, 131.7, 131.4 (d, *J*_C-F_ = 7.5 Hz), 130.1, 129.4, 128.5, 127.8, 125.6 (d, *J*_C-F_ = 3.0 Hz), 125.3, 123.5, 121.1, 116.5 (d, *J*_C-F_ = 2.0 Hz) 111.3, 100.1.


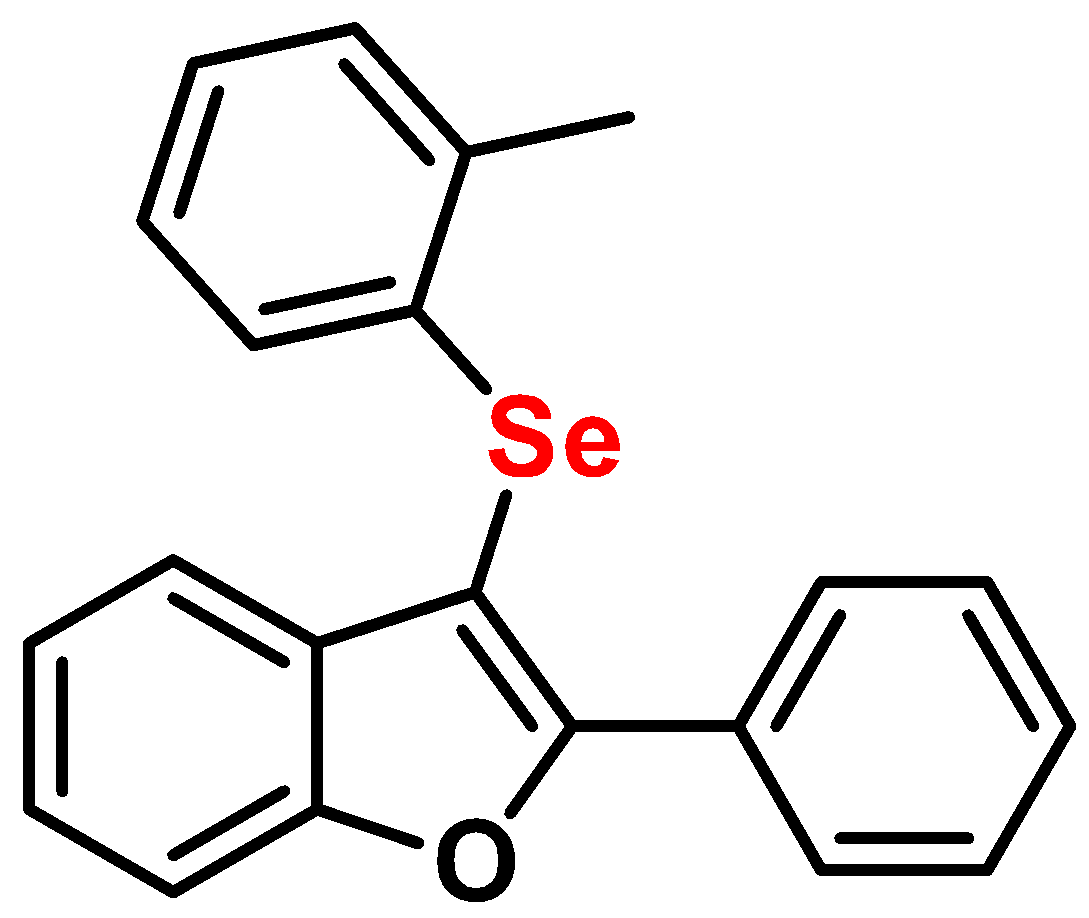
2-phenyl-3-(o-tolylselanyl)benzofuran (**3e**)

Yellow Solid (70.9 mg, 78% yield): ^1^H NMR (400 MHz, CDCl_3_) *δ* 8.29 – 8.18 (m, 2H), 7.62 (d, *J* = 8.0 Hz, 1H), 7.56 – 7.36 (m, 5H), 7.27 (dd, *J* = 8.0, 6.5 Hz, 1H), 7.21 (d, *J* = 7.5 Hz, 1H), 7.10 (td, *J* = 7.5, 1.5 Hz, 1H), 7.04 – 6.89 (m, 2H), 2.53 (s, 3H). ^13^C NMR (100 MHz, CDCl_3_) *δ* 157.6, 154.2, 136.7, 132.0, 131.9, 130.2, 130.1, 129.3, 128.5 128.4, 127.8, 126.8, 126.0, 125.2, 123.4, 121.2, 111.2, 99.1, 21.4. HRMS-ESI: [M+H]^+^ calcd. for C_21_H_17_OSe: 365.0445, found 365.0446.


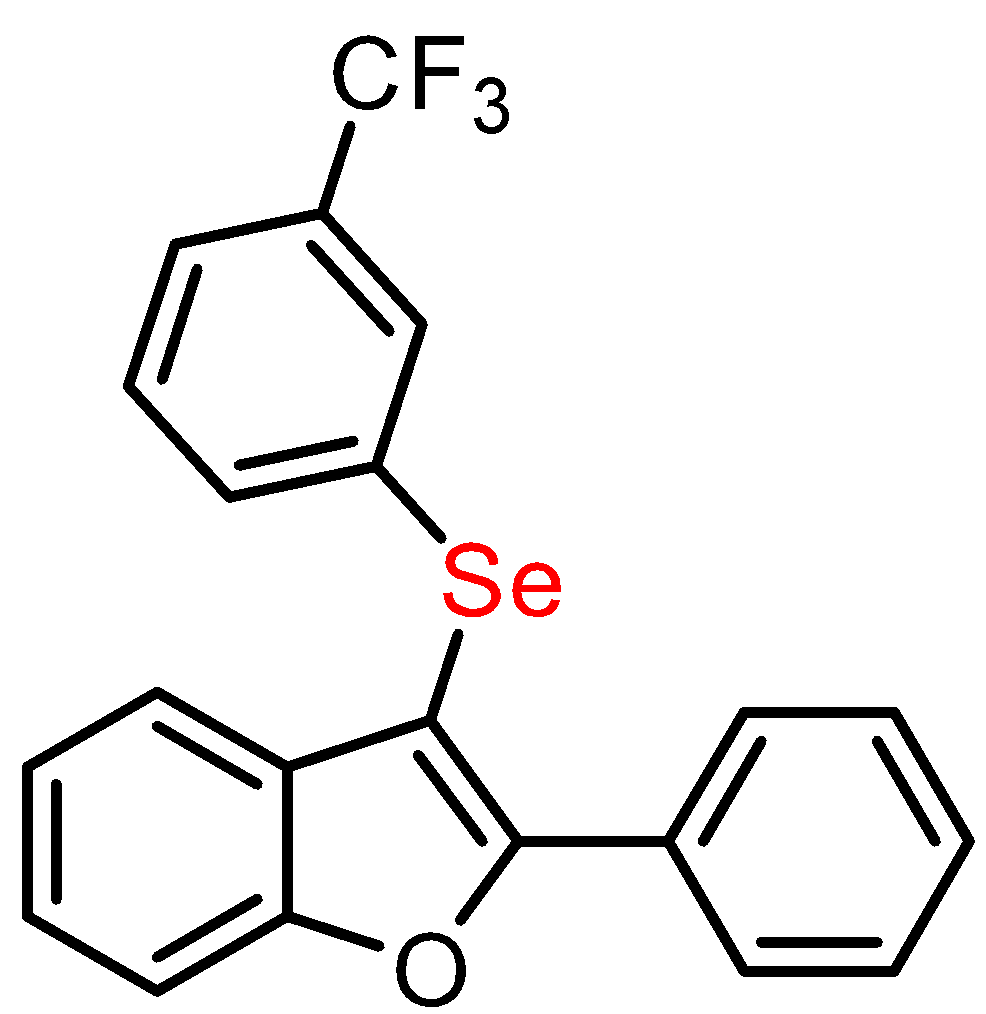
2-phenyl-3-((3-(trifluoromethyl)phenyl)selanyl)benzofuran (**3f**)^5^

Yellow Solid (74.2 mg, 71% yield): ^1^H NMR (400 MHz, CDCl_3_) *δ* 8.26 – 8.13 (m, 2H), 7.67 – 7.57 (m, 2H), 7.58 – 7.33 (m, 6H), 7.27 (dt, *J* = 12.5, 7.5 Hz, 2H). ^13^C NMR (100 MHz, CDCl_3_) *δ* 157.9, 154.3, 135.0, 132.9, 132.2, 131.8, 131.6 (2xC), 131.5, 131.4, 130.0, 129.8, 129.8, 129.7, 128.7, 127.9, 125.7, 125.7, 125.7, 125.6(2x), 125.1, 125.1, 125.0, 125.0, 125.0, 123.8, 123.2, 123.2, 123.2, 123.1, 122.4, 121.0, 111.5, 98.9.


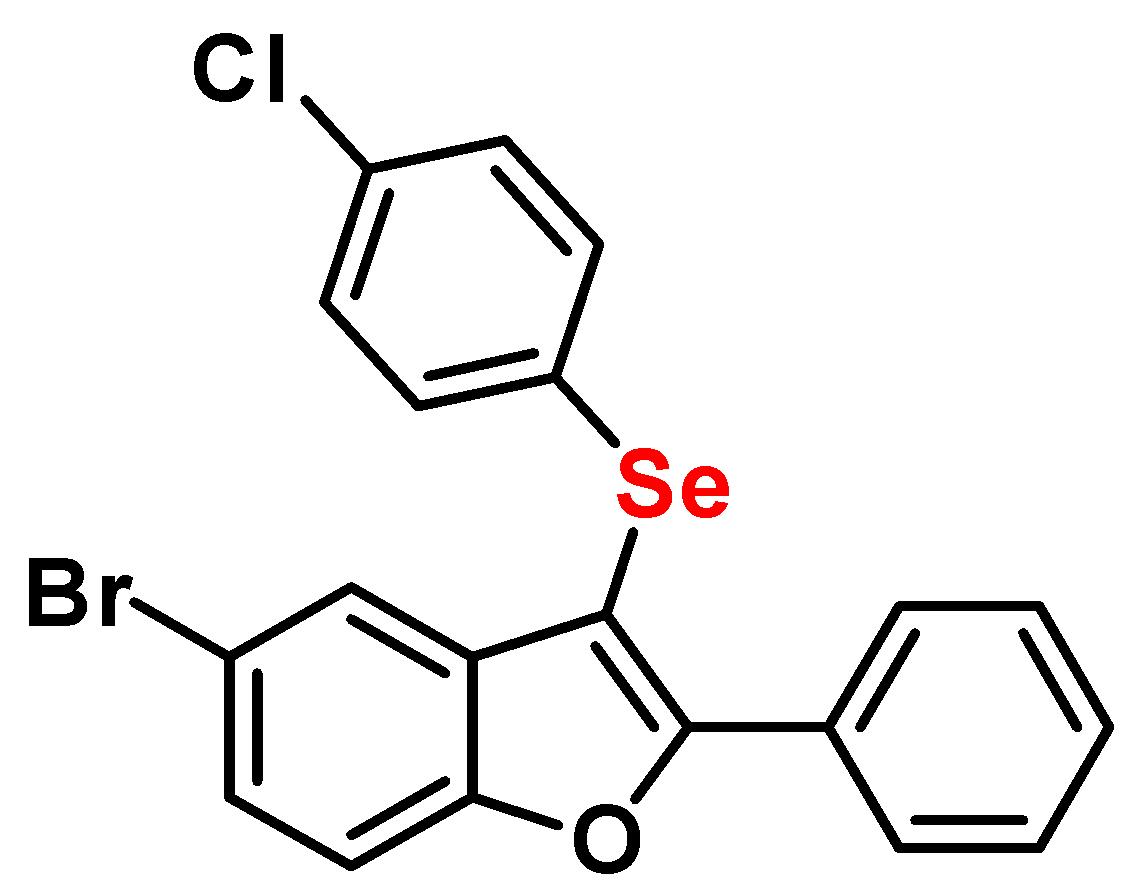
5-bromo-3-((4-chlorophenyl)selanyl)-2-phenylbenzofuran (**3g**)

White Solid (98.1 mg, 85% yield): ^1^H NMR (200 MHz, CDCl_3_) *δ* 7.97 – 7.82 (m, 2H), 7.36 – 7.35 (m, 1H), 7.26 – 7.08 (m, 5H), 7.00 – 6.78 (m, 4H). ^13^C NMR (50 MHz, CDCl_3_) *δ* 158.6, 152.8, 133.8, 132.6, 130.4, 129.8, 129.5, 129.4, 129.1, 128.5, 128.3, 127.8, 123.6, 116.8, 112.7, 98.7. HRMS-APCI: [M]^+^ calcd. for C_20_H_12_BrClOSe: 461.8925, found 461.8902.


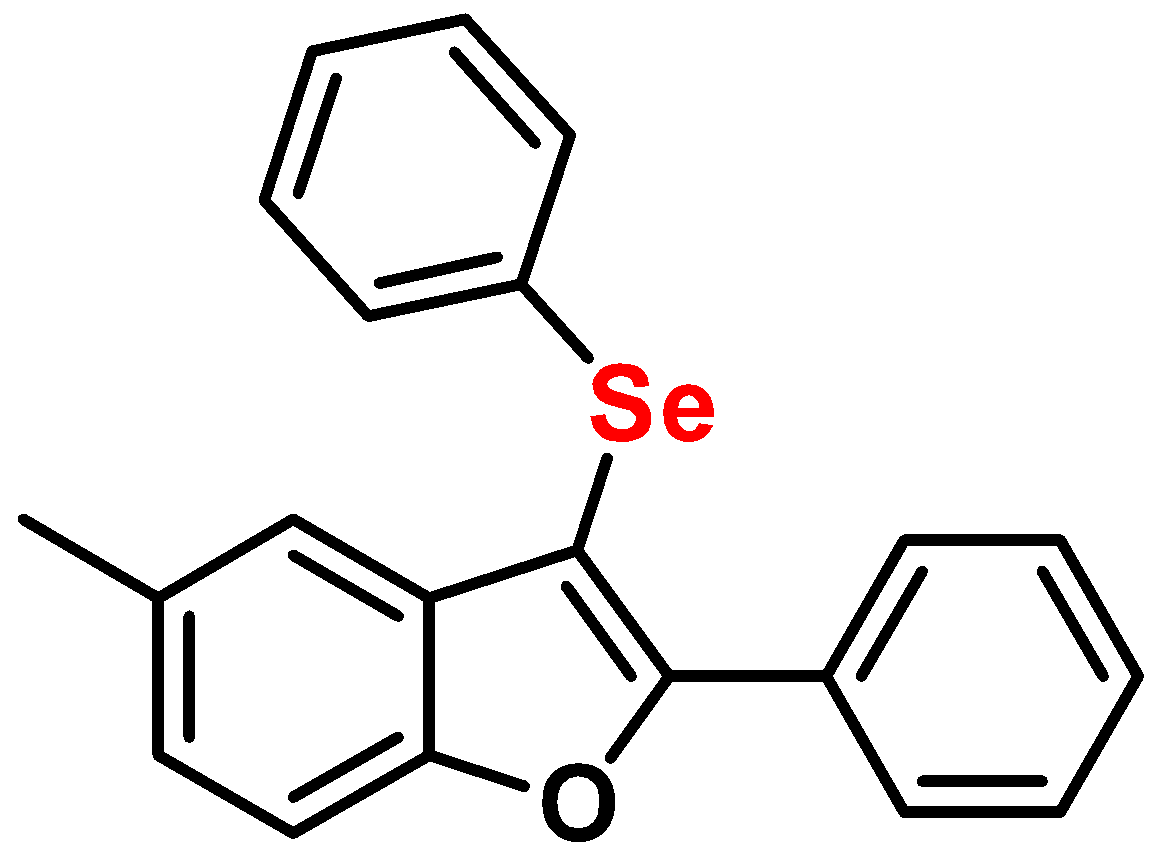
5-methyl-2-phenyl-3-(phenylselanyl)benzofuran (**3h**)^7^

Yellow Solid (86.5 mg, 95% yield): ^1^H NMR (200 MHz, CDCl_3_) *δ* 8.12 (dd, *J* = 7.5, 2.0 Hz, 2H), 7.59 – 7.44 (m, 2H), 7.37 – 7.06 (m, 9H), 2.38 (s, 3H). ^13^C NMR (50 MHz, CDCl_3_) *δ* 157.6, 154.0, 139.4, 132.04, 131.5, 129.2, 129.1, 127.7, 127.3, 126.1, 125.0, 123.37, 121.0, 111.1, 98.9, 21.4.


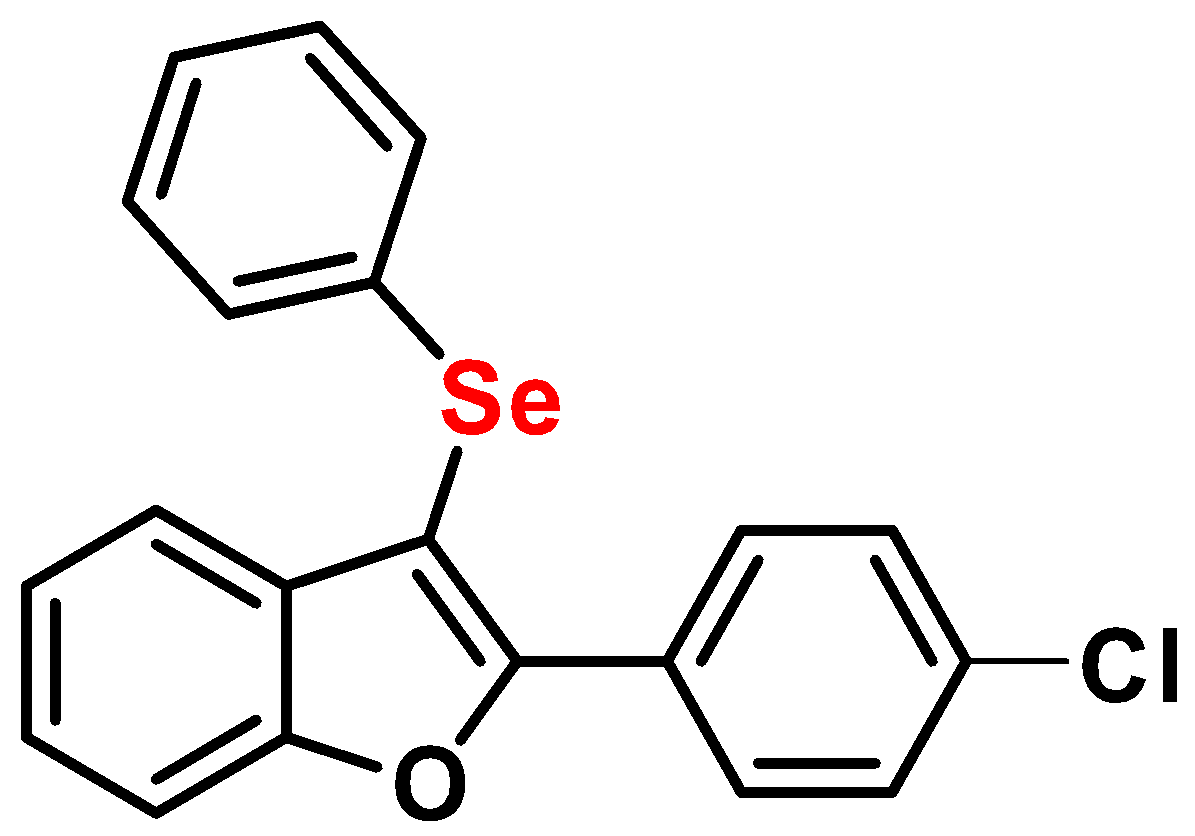
 2-(4-chlorophenyl)-3-(phenylselanyl)benzofuran (**3i**)^5^

Yellow Solid (85.4 mg, 89% yield): ^1^H NMR (200 MHz, CDCl_3_) δ 8.23 – 8.09 (m, 2H), 7.60 – 7.07 (m, 11H). ^13^C NMR (50 MHz, CDCl_3_) *δ* 155.9, 154.0, 135.2, 131.8, 131.1, 129.3, 129.2, 128.9, 128.7, 128.6, 126.4, 125.5, 123.5, 121.2, 111.1, 100.2.


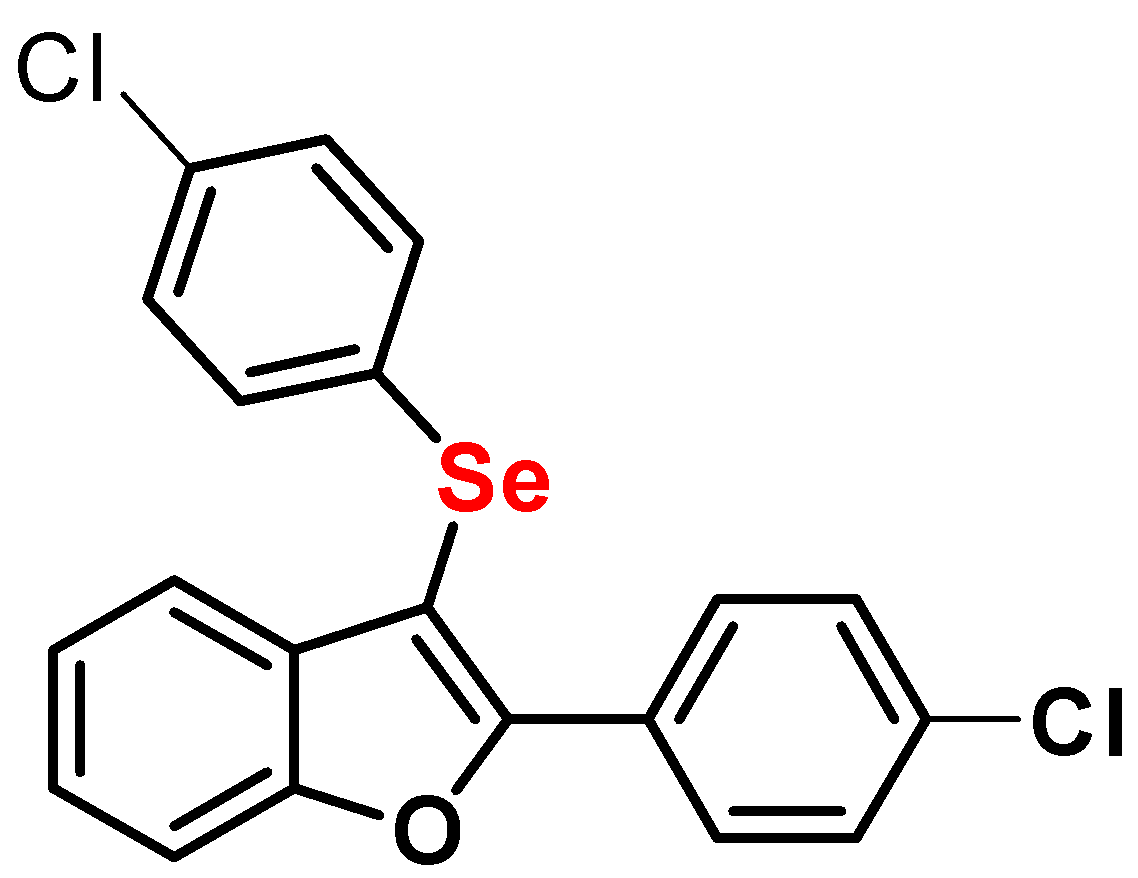
2-(4-chlorophenyl)-3-((4-chlorophenyl)selanyl)benzofuran (**3j**)

White Solid (94.0 mg, 90% yield): ^1^H NMR (200 MHz, CDCl_3_) δ 7.88 (d, *J* = 9.0, 2.0 Hz, 2H), 7.34 – 6.79 (m, 10H). ^13^C NMR (50 MHz, CDCl_3_) δ 156.0, 154.0, 135.4, 132.5, 131.5, 130.5, 129.5, 129.3, 128.8, 128.4, 125.6, 123.7, 121.0, 111.3, 99.9. HRMS-APCI: [M]^+^ calcd. for C_20_H_12_Cl_l2_OSe: 417.9430, found 417.9409.


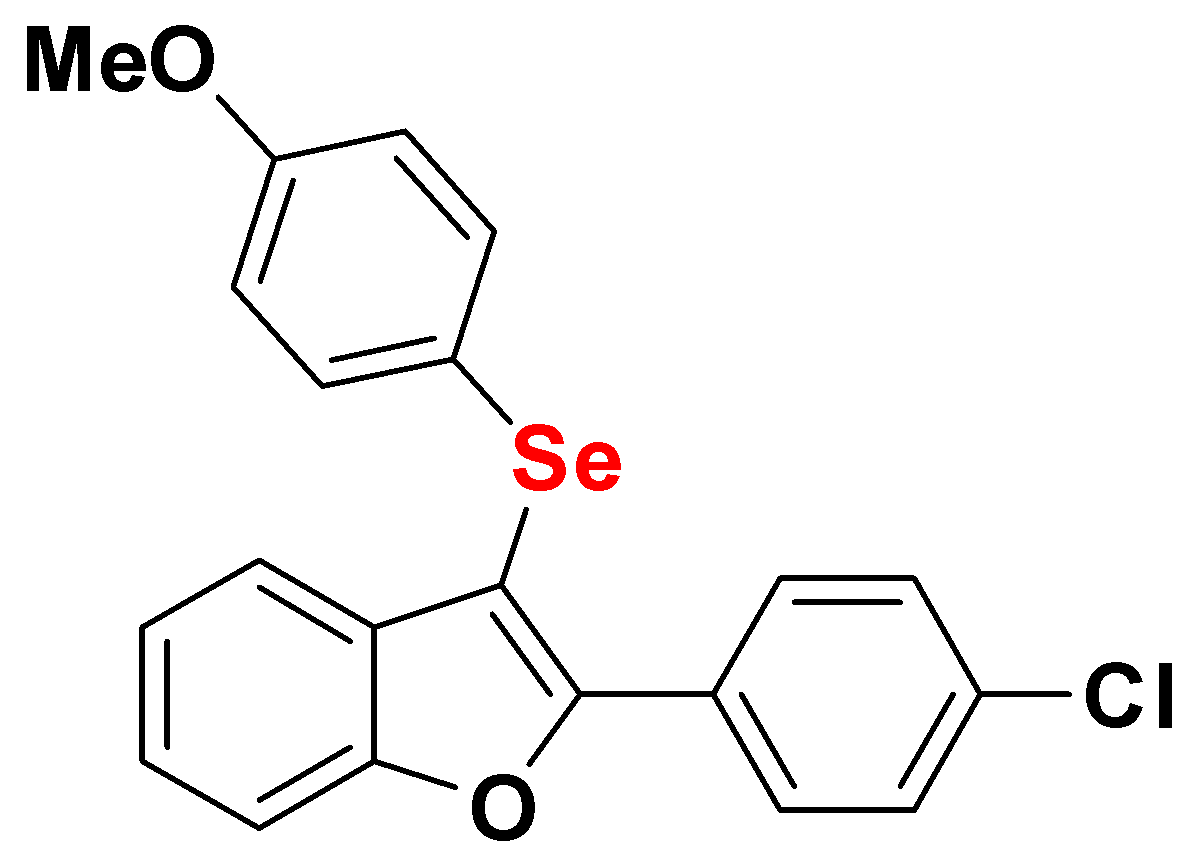
2-(4-chlorophenyl)-3-((4-methoxyphenyl)selanyl)benzofuran (**3k**)

White Solid (51.6 mg, 50% yield): ^1^H NMR (200 MHz, CDCl_3_) δ 8.19 (d, *J* = 9.0 Hz, 2H), 7.56 – 7.17 (m, 8H), 6.72 (d, *J* = 9.0 Hz, 2H), 3.71 (s, 3H). ^13^C NMR (50 MHz, CDCl_3_) δ 158.9, 155.2, 153.9, 135.1, 131.9, 128.8, 128.6, 125.3, 123.4, 121.2, 120.7, 115.1, 111.1, 101.5, 55.2. HRMS-ESI: [M+OH]^+^ calcd. for C_21_H_16_ClO_3_Se: 430.9953, found 430.9798.


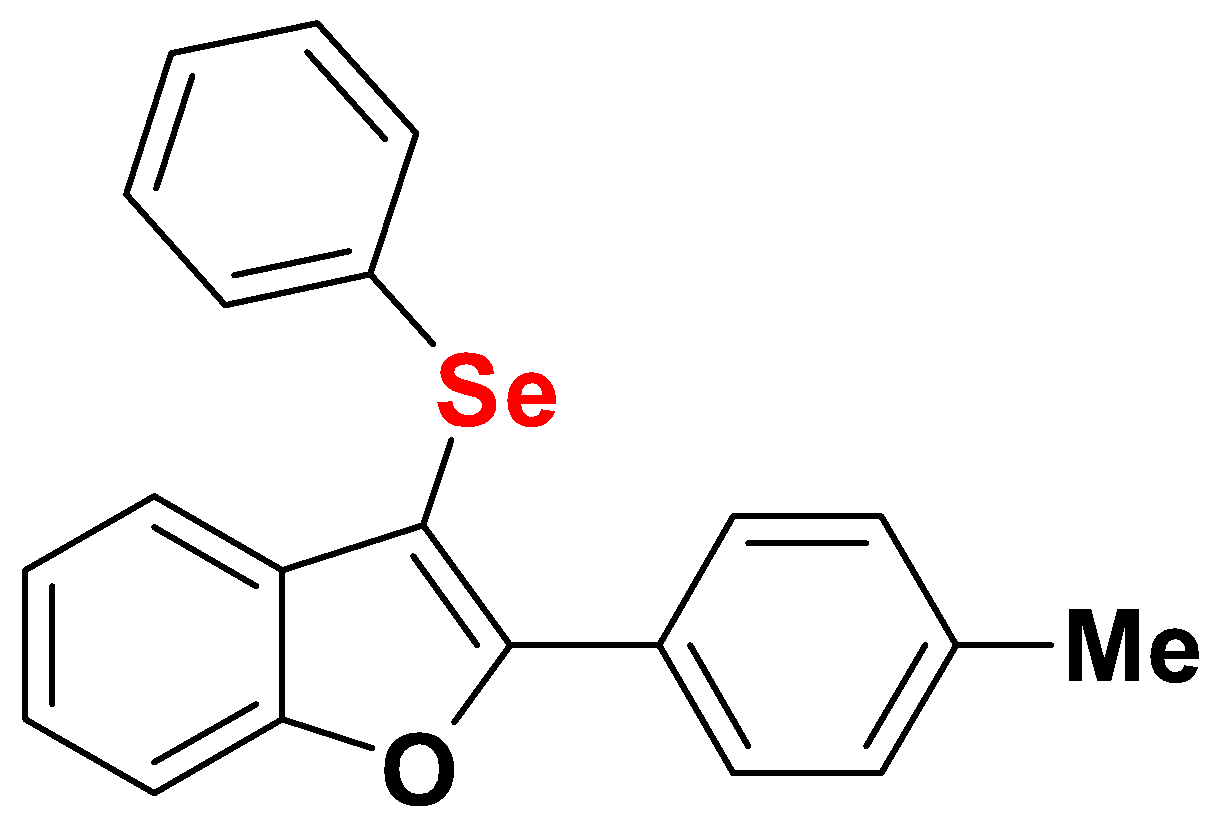
3-(phenylselanyl)-2-(p-tolyl)benzofuran (**3l**)^5^

White Solid (87.0 mg, 98%): ^1^H NMR (200 MHz, CDCl_3_) δ 8.10 (d, *J* = 8.2 Hz, 2H), 7.51 (t, *J* = 7.6 Hz, 2H), 7.37 – 7.00 (m, 9H), 2.38 (s, 3H). ^13^C NMR (50 MHz, CDCl_3_) δ 157.6, 154.1, 139.5, 132.0, 131.6, 129.2, 129.1, 127.7, 127.3, 126.2, 124.9, 123.4, 121.1, 111.1, 98.8, 21.4.


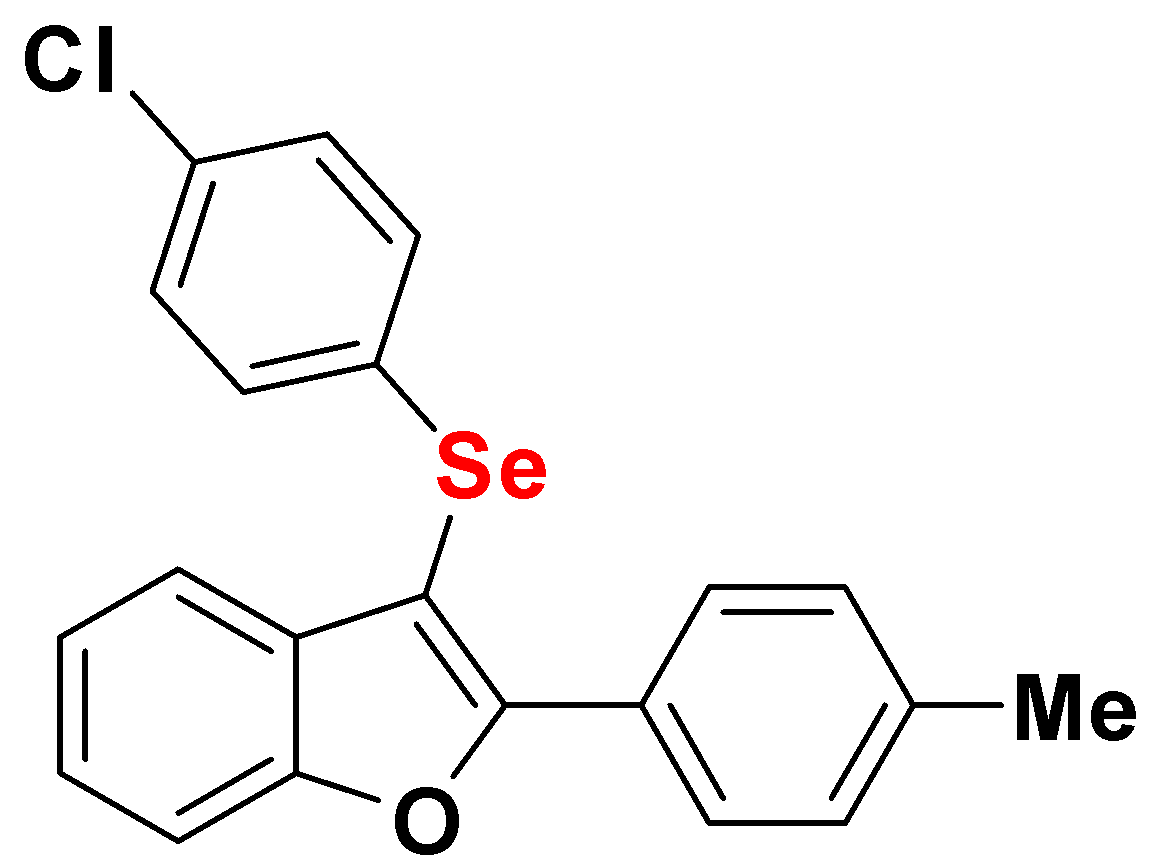
3-((4-chlorophenyl)selanyl)-2-(p-tolyl)benzofuran (**3m**)

Yellow Solid (84.5 mg, 84%): ^1^H NMR (200 MHz, CDCl_3_) δ 8.10 (d, *J* = 8.0 Hz, 2H), 7.51 (t, *J* = 7.5 Hz, 2H), 7.37 – 6.95 (m, 8H), 2.38 (s, 3H). ^13^C NMR (50 MHz, CDCl_3_) δ 157.6, 154.0, 139.5, 132.0, 131.6, 129.2, 127.7, 127.3, 126.2, 124.9, 123.4, 121.1, 111.1, 98.9, 21.4. HRMS-ESI: [M+OH]^+^ calcd. for C_21_H_16_ClO_2_Se: 415.0004, found 414.9989.


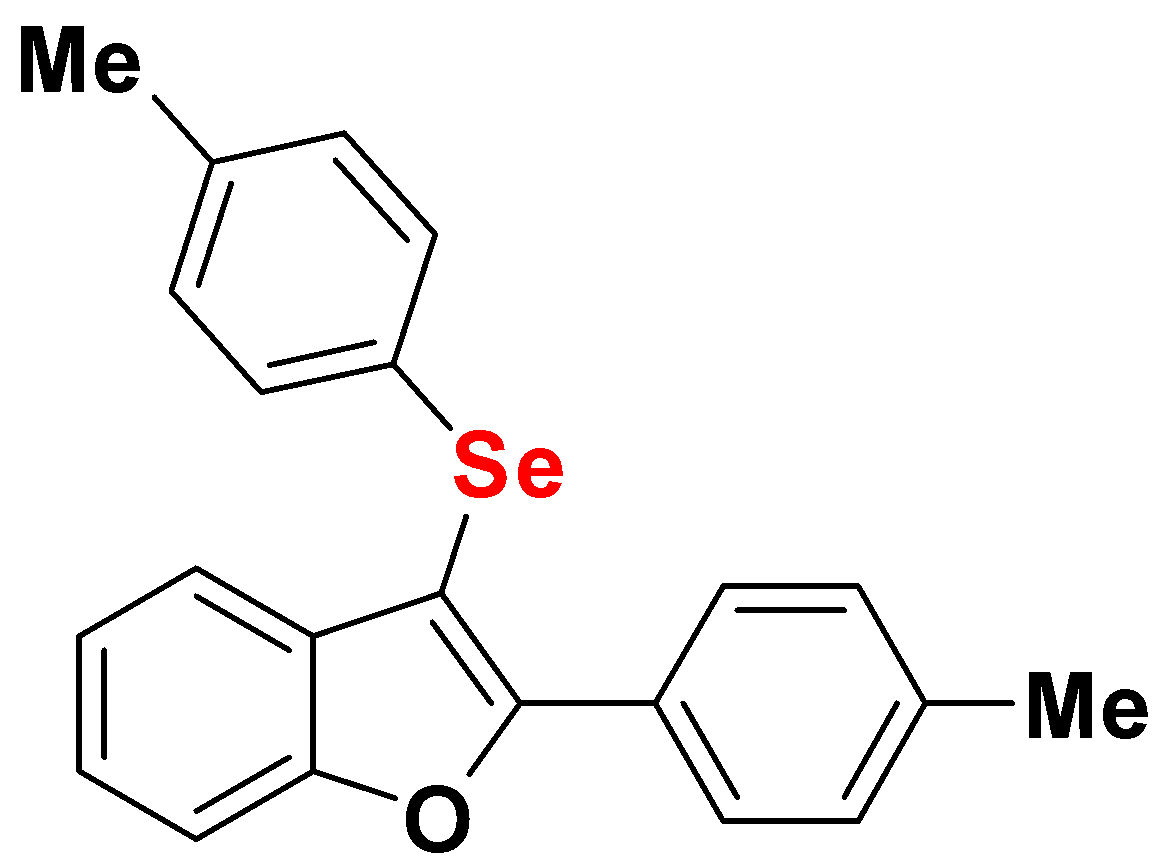


2-(p-tolyl)-3-(p-tolylselanyl)benzofuran (**3n**)

White Solid (85.9 mg, 91%): ^1^H NMR (200 MHz, CDCl_3_) δ 8.10 (d, *J* = 8.0 Hz, 2H), 7.51 (t, *J* = 6.0 Hz, 2H), 7.40 – 7.09 (m, 6H), 6.96 (d, *J* = 8.0 Hz, 2H), 2.39 (s, 3H), 2.23 (s, 3H). ^13^C NMR (50 MHz, CDCl_3_) *δ* 157.3, 154.0, 139.4, 136.1, 132.1, 130.1, 129.5, 129.2, 127.7, 127.4, 124.9, 123.3, 121.1, 111.0, 99.4, 21.4, 20.9. EIMS (*m/z,* rel. int., %) 298 (100), 178 (14), 255 (11), 378 (28). HRMS not ionized in ESI and APCI.


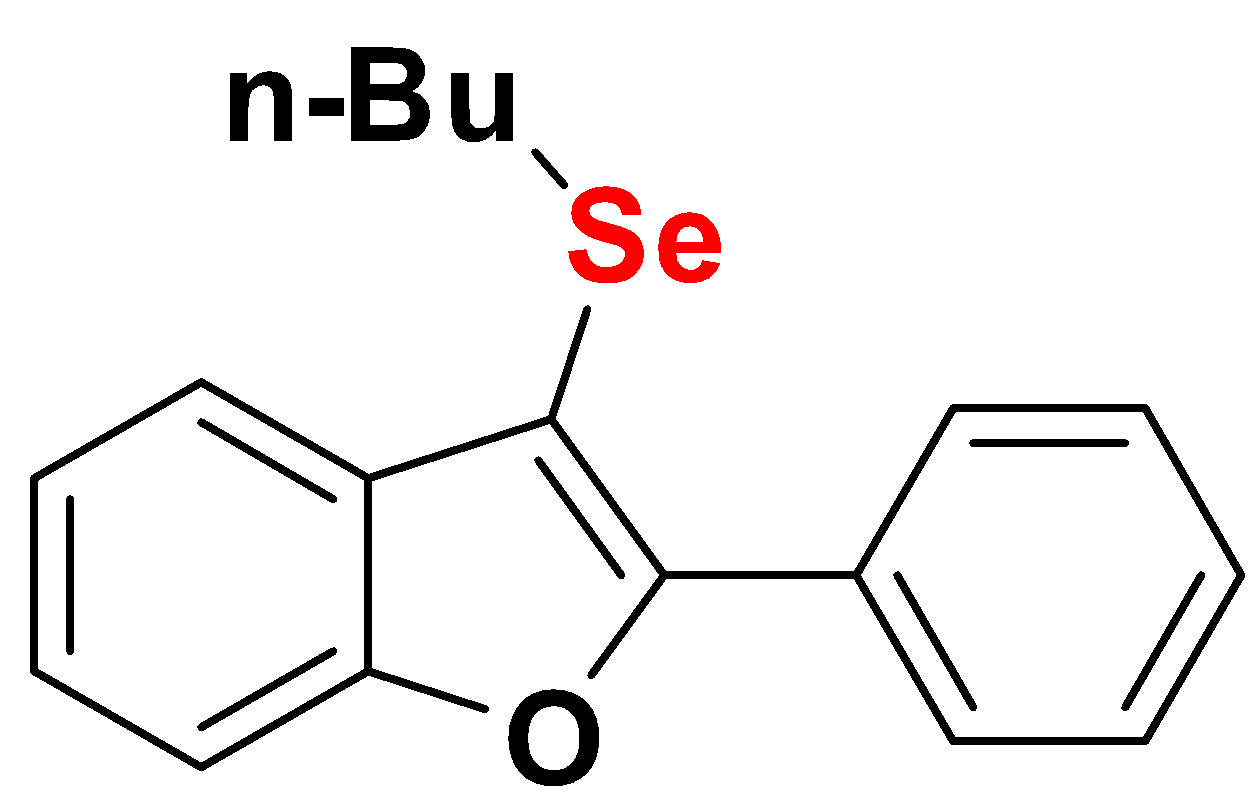
3-(butylselanyl)-2-phenylbenzofuran (**3o**)^5^

Yellow Oil (64.6 mg, 78% yield): ^1^H NMR (400 MHz, CDCl_3_) *δ* 8.39 – 8.29 (m, 2H), 7.71 (m, 1H), 7.58 – 7.27 (m, 6H), 2.82 (t, *J* = 7.5 Hz, 2H), 1.66 – 1.53 (m, 2H), 1.42 – 1.30 (m, 2H), 0.82 (t, *J* = 7.5 Hz, 3H). ^13^C NMR (100 MHz, CDCl_3_) δ 155.9, 153.9, 132.7, 130.7, 128.9, 128.4, 127.7, 124.9, 123.1, 121.0, 111.1, 100.4, 32.4, 28.3, 22.7, 13.5.


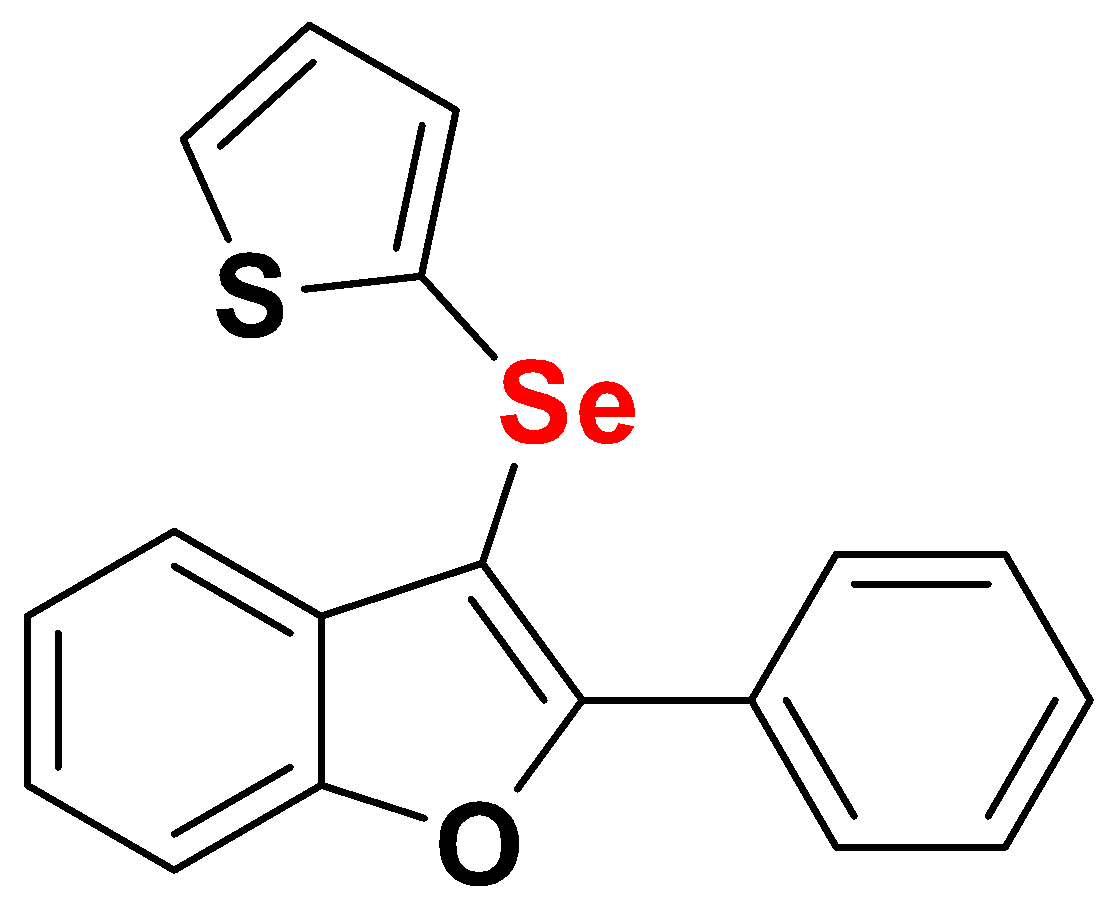
2-phenyl-3-(thiophen-2-ylselanyl)benzofuran (**3p**)

Yellow Solid (27.6 mg, 31% yield): ^1^H NMR (400 MHz, CDCl_3_) *δ* 8.39 – 8.19 (m, 2H), 7.77 – 7.66 (m, 1H), 7.60 – 7.20 (m, 8H), 6.98 – 6.85 (m, 1H). ^13^C NMR (100 MHz, CDCl_3_) *δ* 156.2, 153.9, 133.6, 131.7, 130.2, 129.9, 129.3, 128.5, 128.0, 127.8, 125.2, 123.4, 121.0, 111.2, 102.1. EIMS (*m/z,* rel. int., %) 276 (100), 44 (10), 165 (22), 356 (21). HRMS not ionized in ESI and APCI.

3-(phenylselanyl)-2-(thiophen-3-yl)benzofuran (**3q**)

Pale yellow Solid (80.8 mg, 91% yield): ^1^H NMR (200 MHz, CDCl_3_) *δ* 8.23 – 8.06 (m, 1H), 7.95 (d, *J* = 5.0 Hz, 1H), 7.50 (d, *J* = 8.0 Hz, 2H), 7.41 – 6.92 (m, 9H). ^13^C NMR (50 MHz, CDCl_3_) *δ* 154.7, 154.0, 131.9, 131.4 (2xC), 129.4, 129.3, 126.8, 126.4, 126.0, 125.4, 125.2, 123.6, 121.1, 111.2, 98.9. HRMS-ESI: [M+OH]^+^ calcd. for C_18_H_13_O_2_SSe: 372.9801, found 372.9798.

2,3-bis(phenylselanyl)benzofuran (**4a, 4a’**)^8^

Yellow Solid (77.2 mg, 72% yield): ^1^H NMR (400 MHz, CDCl_3_) *δ* 7.58 – 7.52 (m, 2H), 7.49 (d, *J* = 8.0 Hz, 1H), 7.44 (dd, *J* = 8.0, 1.4 Hz, 1H), 7.37 – 7.16 (m, 10H). ^13^C NMR (100 MHz, CDCl_3_) *δ* 157.3, 150.8, 132.8, 130.7, 130.5, 129.4, 129.2, 128.9, 128.9, 127.9, 126.7, 125.3, 123.5, 121.0, 113.7, 111.4.

2,3-bis(p-tolylselanyl)benzofuran (**4b**)

Yellow Solid (77.6 mg, 68% yield): ^1^H NMR (400 MHz, CDCl_3_) δ 7.50 – 7.40 (m, 4H), 7.32 – 7.18 (m, 4H), 7.09 (d, *J* = 8.0 Hz, 2H), 7.00 (d, *J* = 8.0 Hz, 2H), 2.34 (s, 3H), 2.29 (s, 3H). ^13^C NMR (100 MHz, CDCl_3_) *δ* 157.1, 138.1, 136.7, 133.3, 130.9, 130.5, 130.2, 130.0, 126.8, 125.1, 123.4, 120.9, 113.4, 111.3, 21.2, 21.0. HRMS-APCI: [M+H]^+^ calcd. for C_22_H_19_OSe_2_: 458,9766, found 458.9756.

2,3-bis((4-chlorophenyl)selanyl)benzofuran (**4c**)

Yellow Solid (83.1 mg, 67% yield): ^1^H NMR (400 MHz, CDCl_3_) *δ* 7.50 (d, *J* = 8.3 Hz, 1H), 7.47 – 7.41 (m, 2H), 7.34 (td, *J* = 8.3, 7.2, 1.4 Hz, 1H), 7.29 – 7.18 (m, 4H), 7.17 – 7.10 (m, 1H). ^13^C NMR (100 MHz, CDCl_3_) *δ*  157.3, 134.6, 134.4, 133.1, 131.8, 131.2, 130.2, 129.7, 129.5, 128.9, 126.9, 125.8, 123.9, 120.9, 113.5, 111.6*.* HRMS-APCI: [M+OH]^+^ calcd. for C_20_H_13_Cl_2_O_2_Se_2_:514.8602, found 514.8602.

^^3-(phenylselanyl)-2-(phenylthio)benzofuran (**4d**)^8^

Yellow solid (83.7 mg, 88% yield):  ^1^H NMR (200 MHz, CDCl_3_) *δ* 7.45 – 7.37 (m, 2H), 7.36 – 7.11 (m, 12H). ^13^C NMR (50 MHz, CDCl_3_) *δ* 156.4, 152.9, 133.4, 131.0, 130.5, 130.3, 129.3, 127.6, 126.90, 125.9, 123.6, 121.3, 112.8, 111.6.

2-((phenylselanyl)ethynyl)phenol (**1g**)

Brown solid (90.6 mg, 33% yield): ^1^H NMR (400 MHz, CDCl_3_) *δ* 7.60 – 7.54 (m, 1H), 7.40 (dd, *J* = 7.5, 1.5 Hz, 1H), 7.36 – 7.24 (m, 4H), 7.01 – 6.93 (m, 1H), 6.89 (td, *J* = 7.5, 1.0 Hz, 1H), 5.91 (s, 1H). ^13^C NMR (100 MHz, CDCl_3_) *δ* 157.7, 132.5, 131.3, 129.9, 129.4, 128.6, 127.6, 120.5, 115.0, 109.7, 96.7, 77.2. HRMS-ESI: [M+H]^+^ calcd. for C_14_H_11_OSe: 274.9975, found 274.9988.

2-((phenylthio)ethynyl)phenol (**1h**)

Yellow solid (236.2 mg, 54% yield): ^1^H NMR (200 MHz, CDCl_3_) *δ* 7.58 – 7.13 (m, 7H), 6.92 (dd, *J* = 18.5, 8.0 Hz, 2H), 5.90 (s, 1H). ^13^C NMR (50 MHz, CDCl_3_) *δ* 157.8, 132.9, 132.5, 131.5, 129.6, 127.0, 126.6, 120.6, 115.1, 109.4, 91.6, 83.4. HRMS-ESI: [M-H]^-^ calcd. for C_14_H_9_OS: 225.0374, found 225.0370.

2-phenyl-3-(phenylseleninyl)benzofuran (**5a**)

White Solid (160.7 mg, 88% yield): ^1^H NMR (400 MHz, CDCl_3_) *δ* 7.97 – 7.91 (m, 2H), 7.82 (dd, *J* = 7.5, 2.0 Hz, 2H), 7.59 – 7.42 (m, 8H), 7.28 (d, *J* = 14.5 Hz, 1H), 7.14 – 7.06 (m, 1H). ^13^C NMR (100 MHz, CDCl_3_) *δ* 158.6, 154.4, 140.1, 131.3, 130.8, 129.8, 129.3, 128.8, 128.4, 126.8, 126.5, 125.7, 124.0, 121.4, 115.1, 111.6. HRMS-ESI: [M+H]^+^ calcd. for C_20_H_15_O_2_Se: 367.0237, found 367.0235.

**7. NRM Spectra**


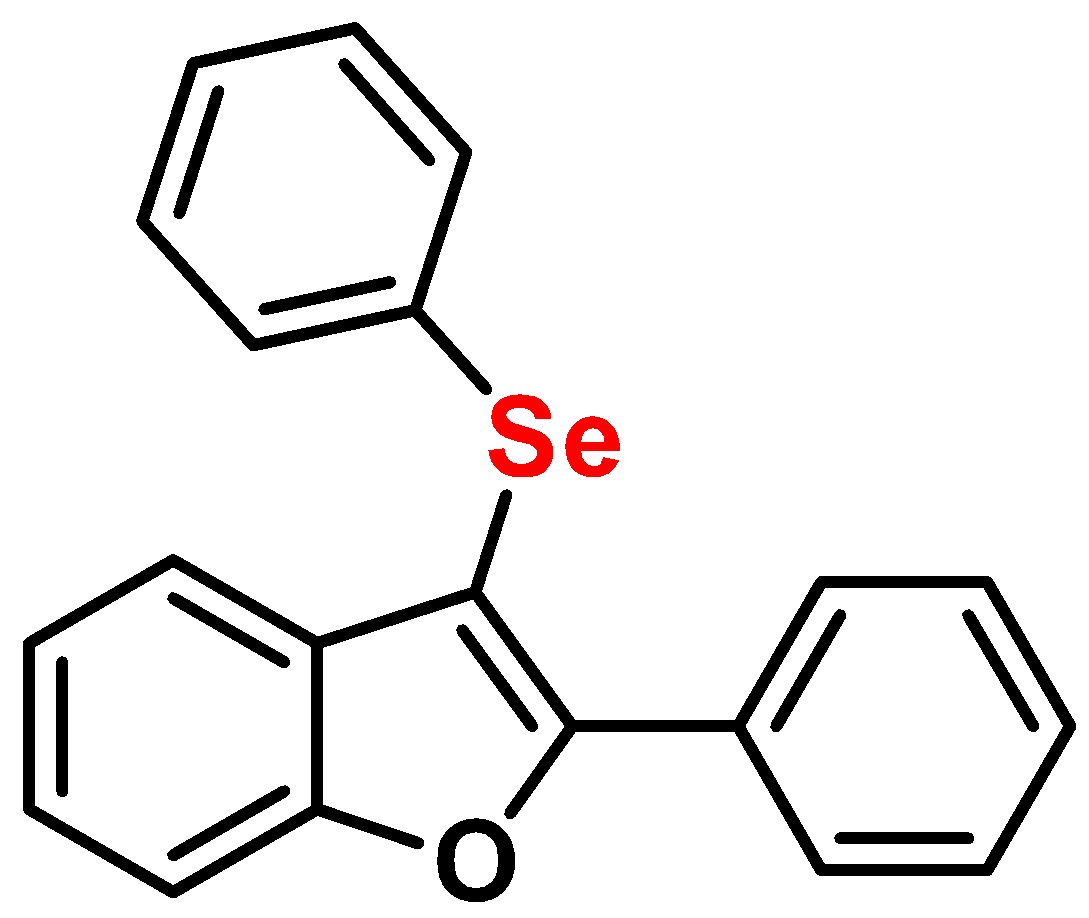

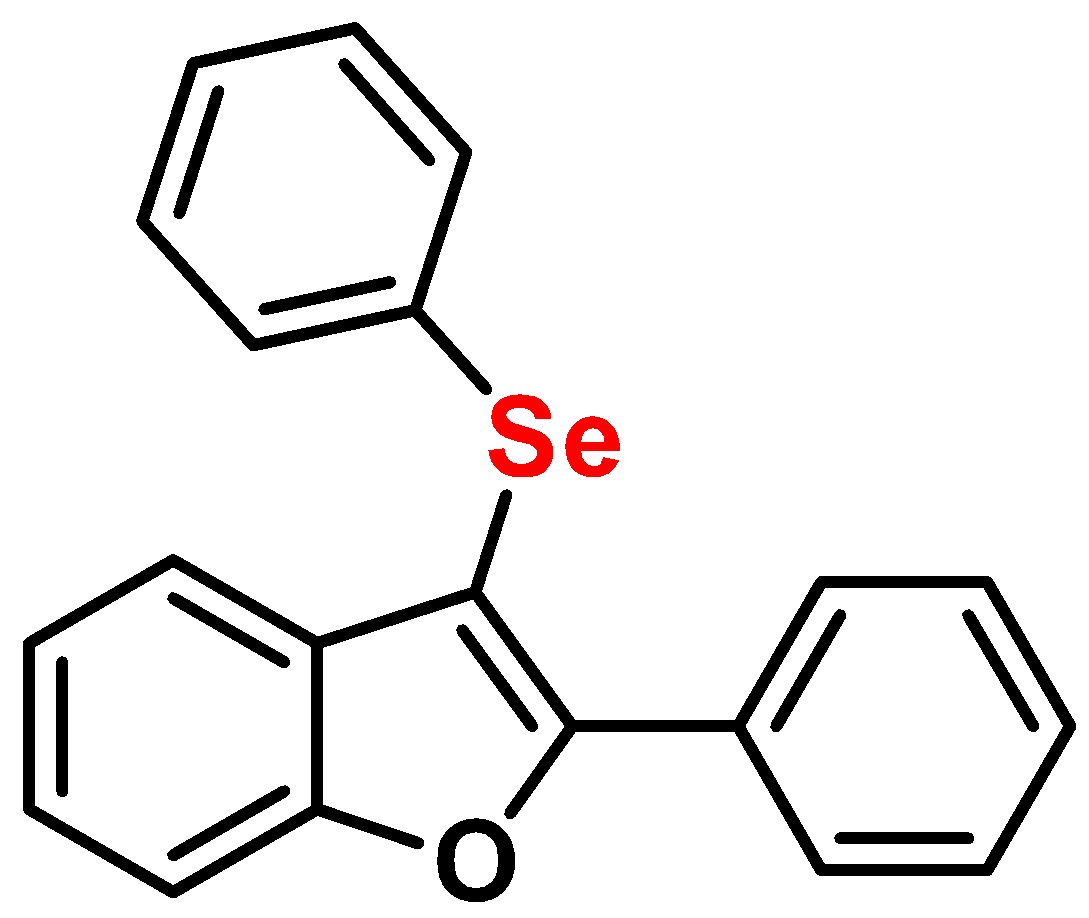


**^1^H NMR (top) and ^13^C NMR (bottom) spectra for 3a in CDCl_3_.**


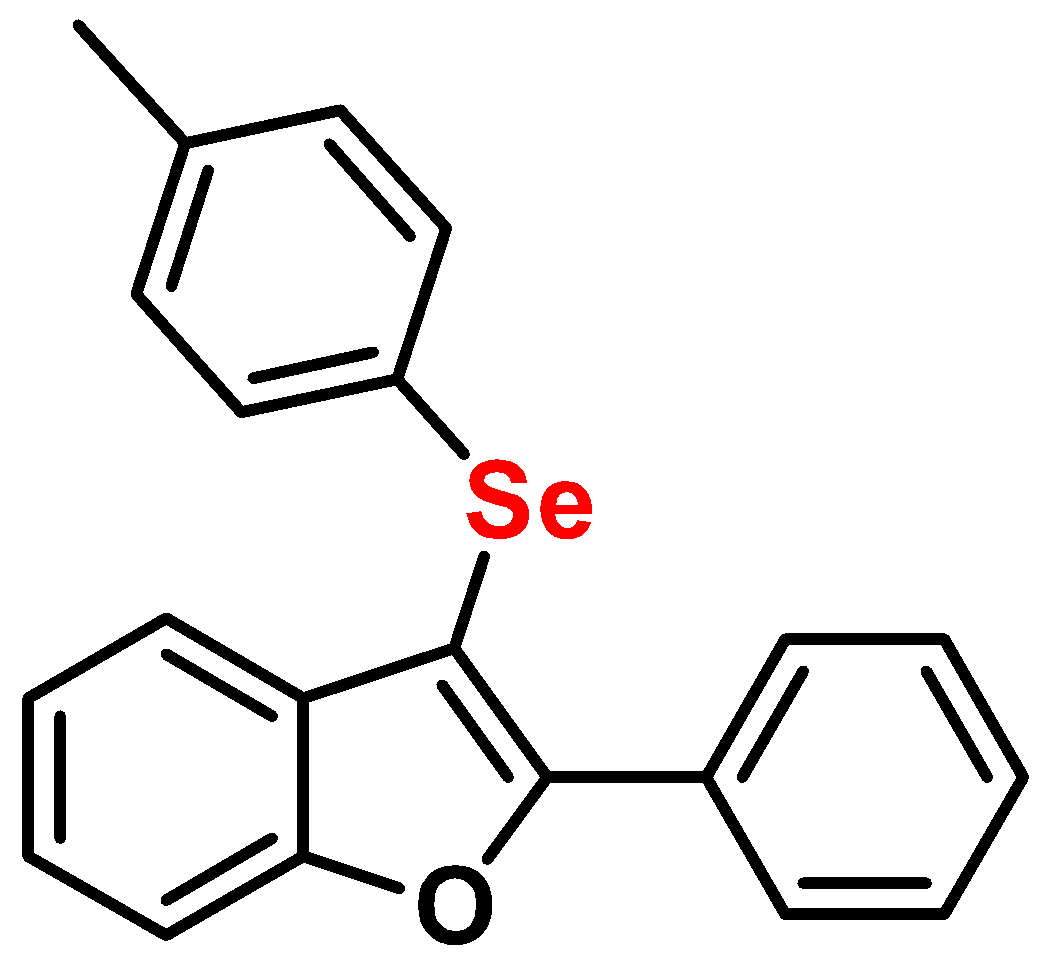


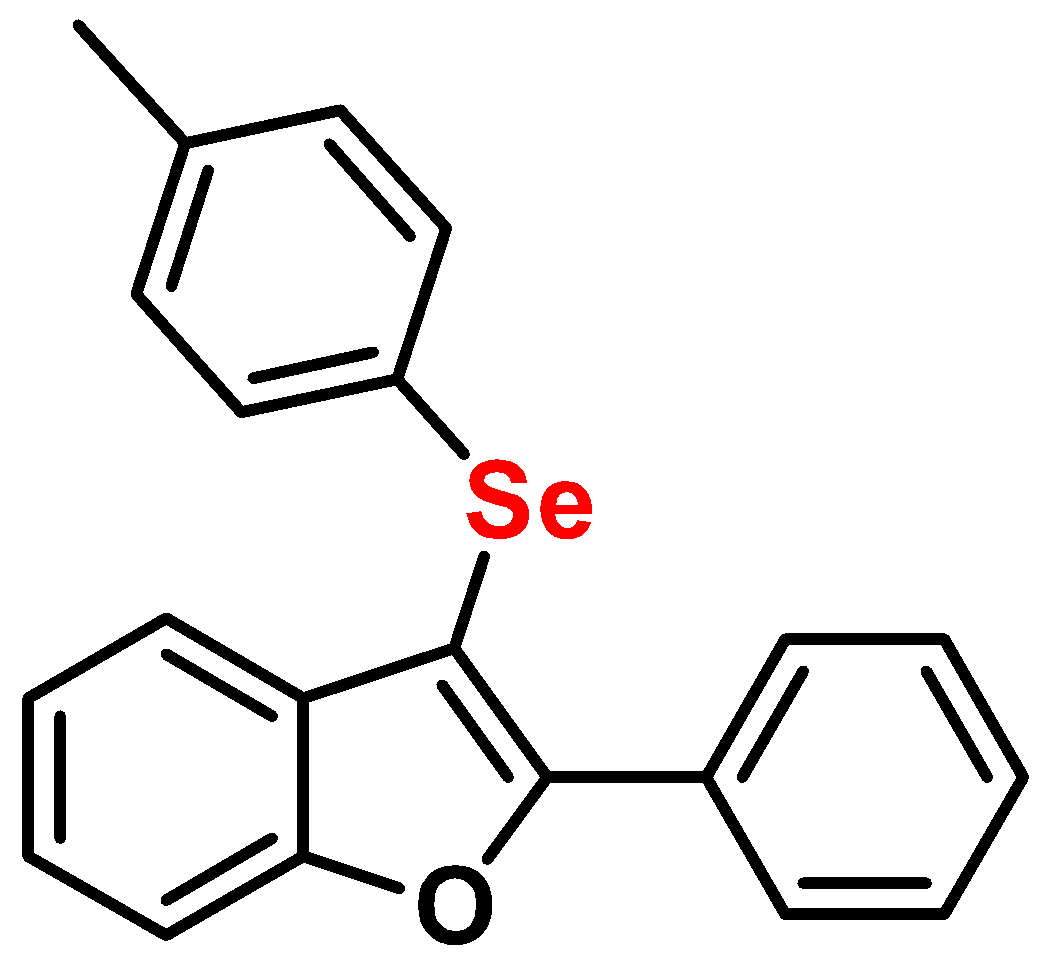


**^1^H NMR (top) and ^13^C NMR (bottom) spectra for 3b in CDCl_3_.**


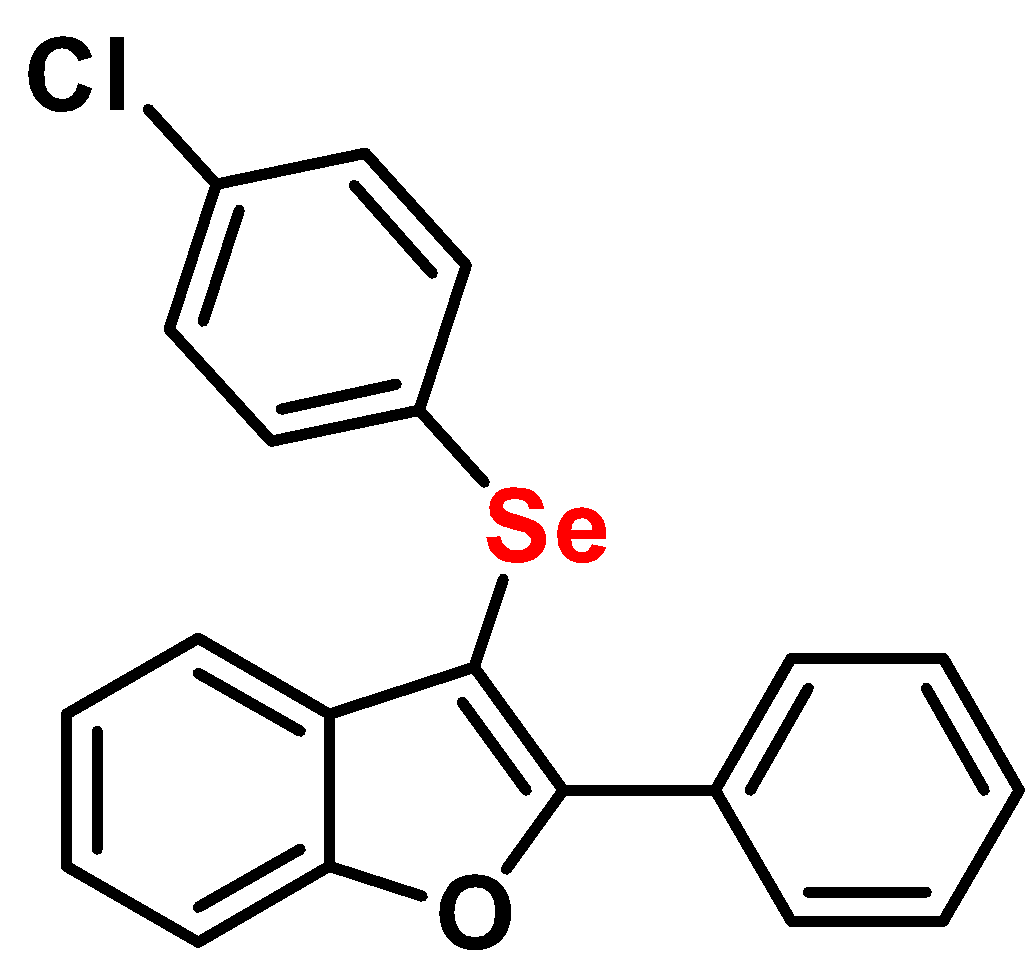


**
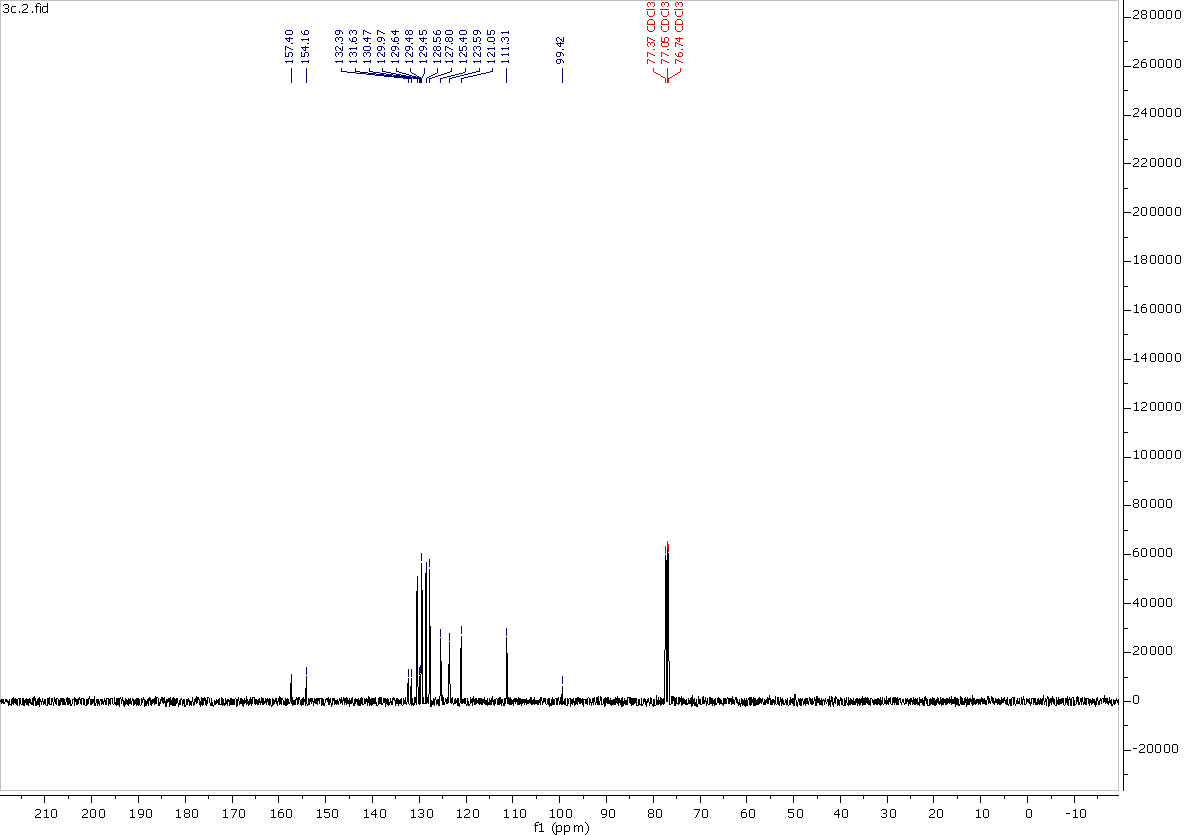
**
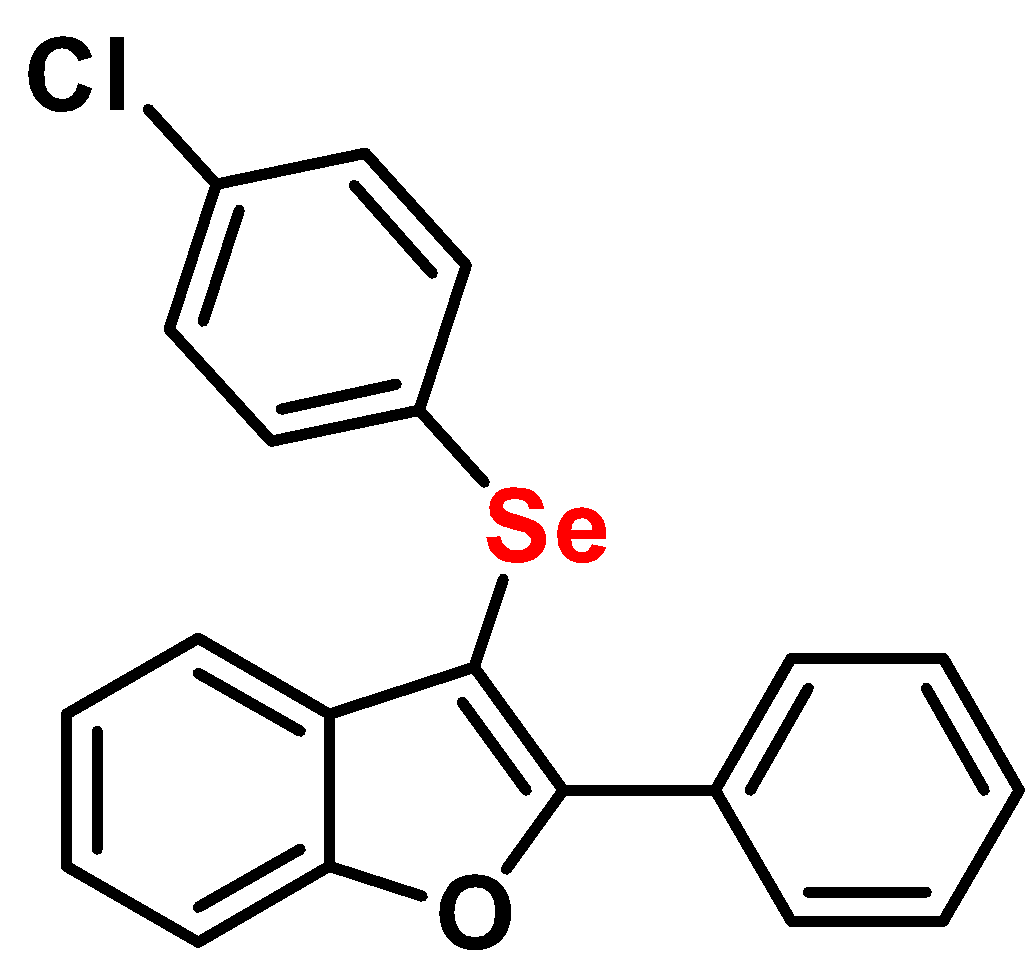


**^1^H NMR (top) and ^13^C NMR (bottom) spectra for Compound 3c in CDCl_3_.**


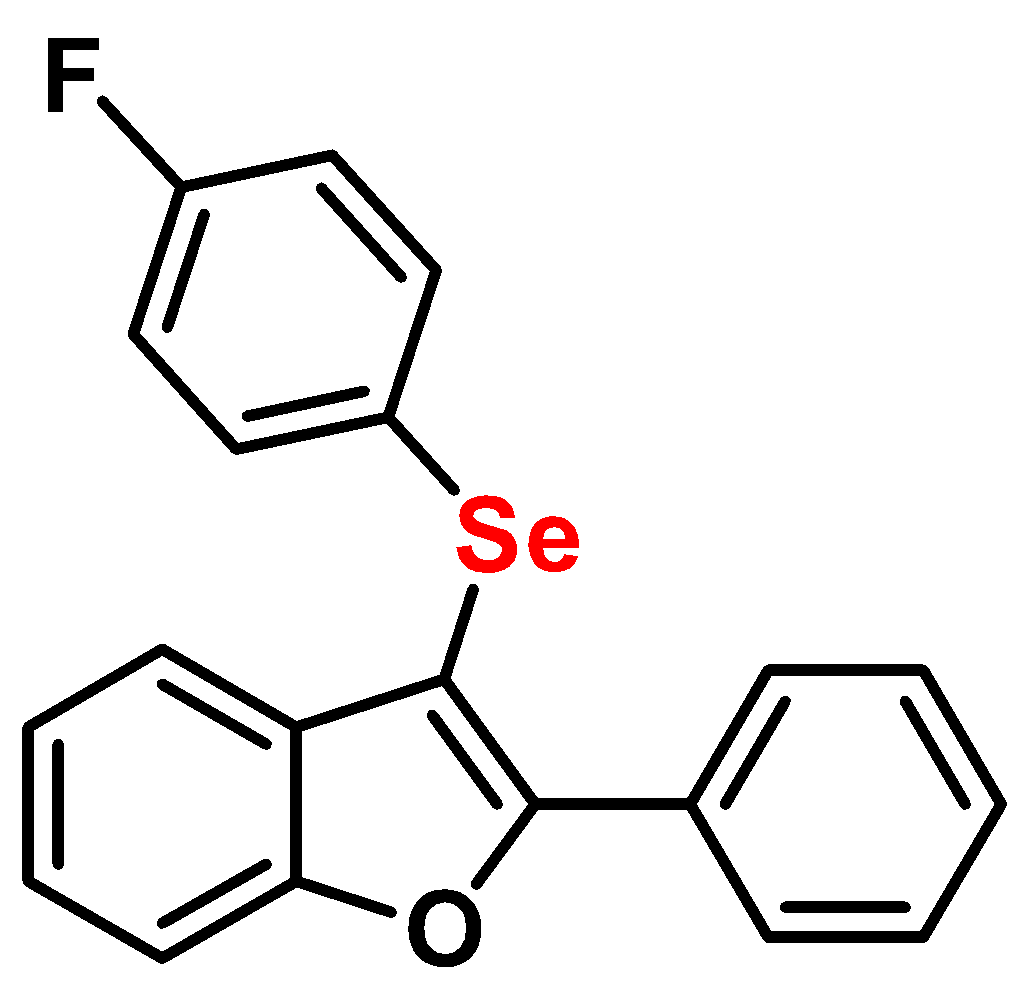


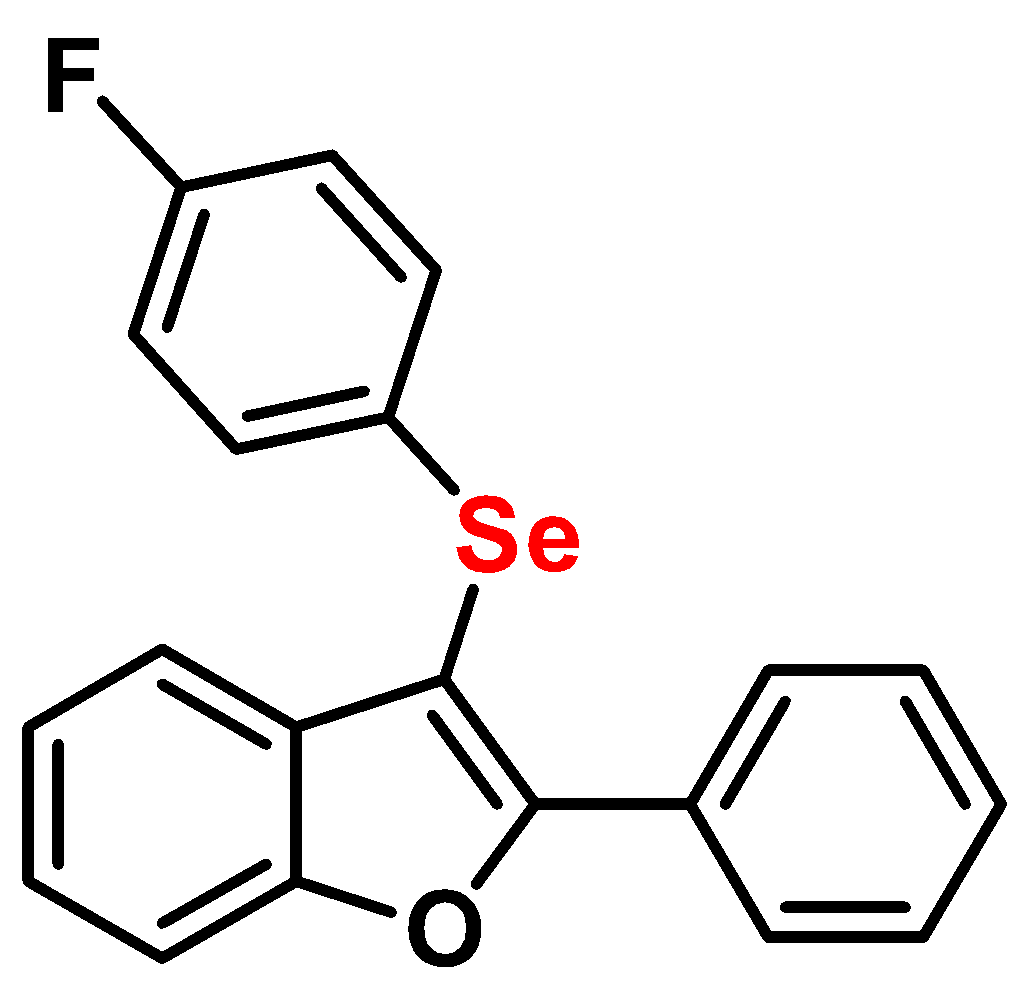


**^1^H NMR (top) and ^13^C NMR (bottom) spectra for Compound 3d in CDCl_3_.**


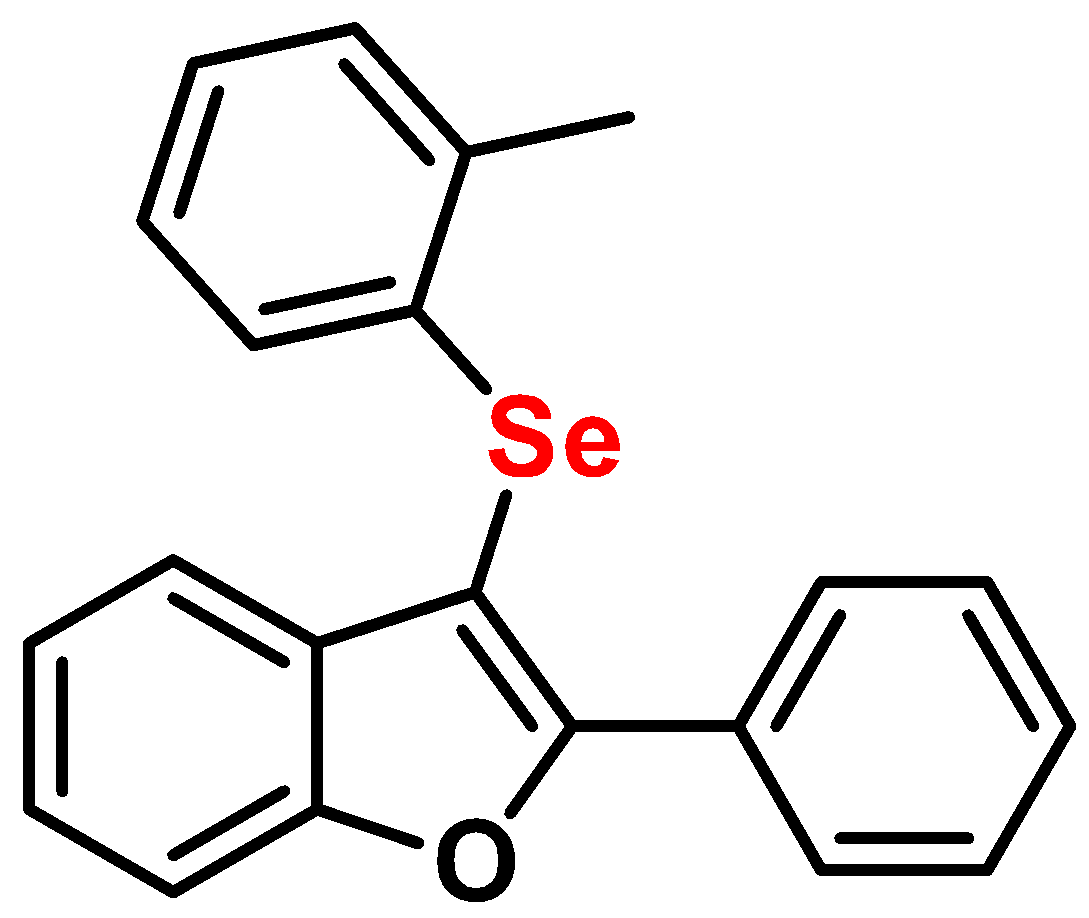

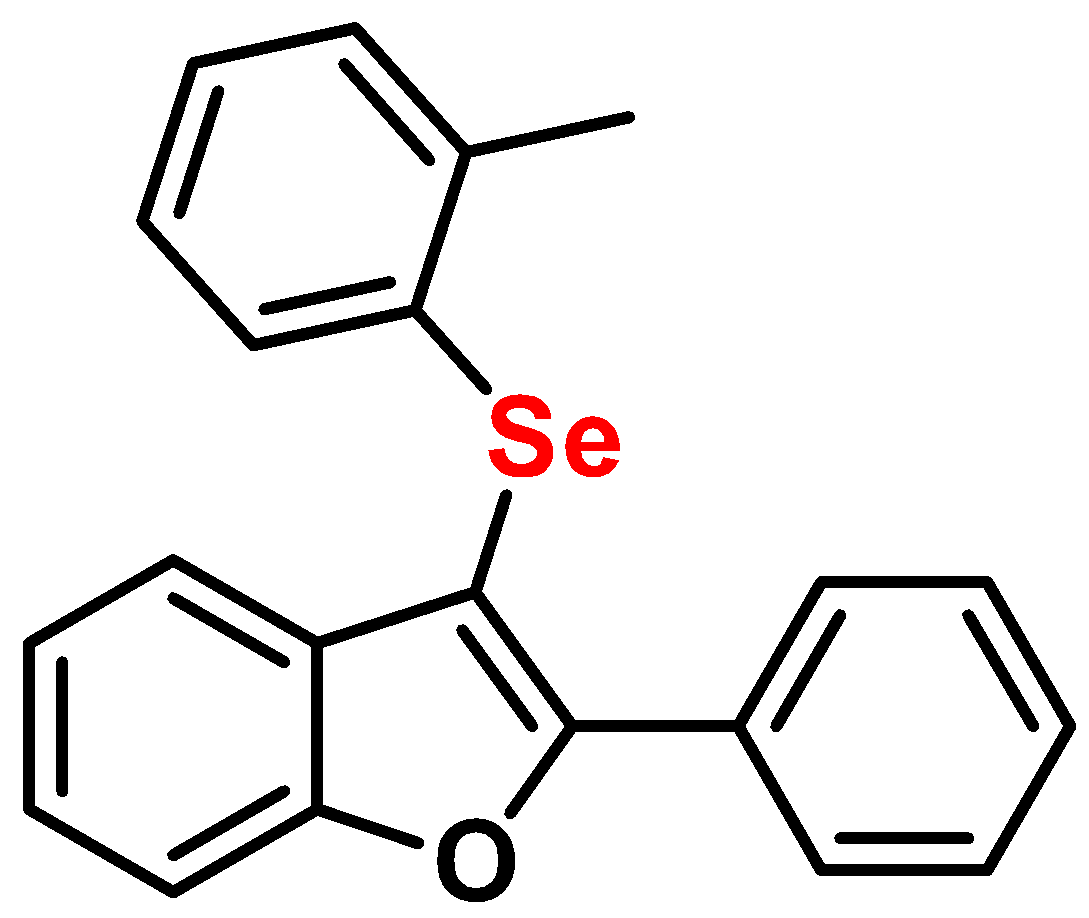


**^1^H NMR (top) and ^13^C NMR (bottom) spectra for Compound 3e in CDCl_3_.**


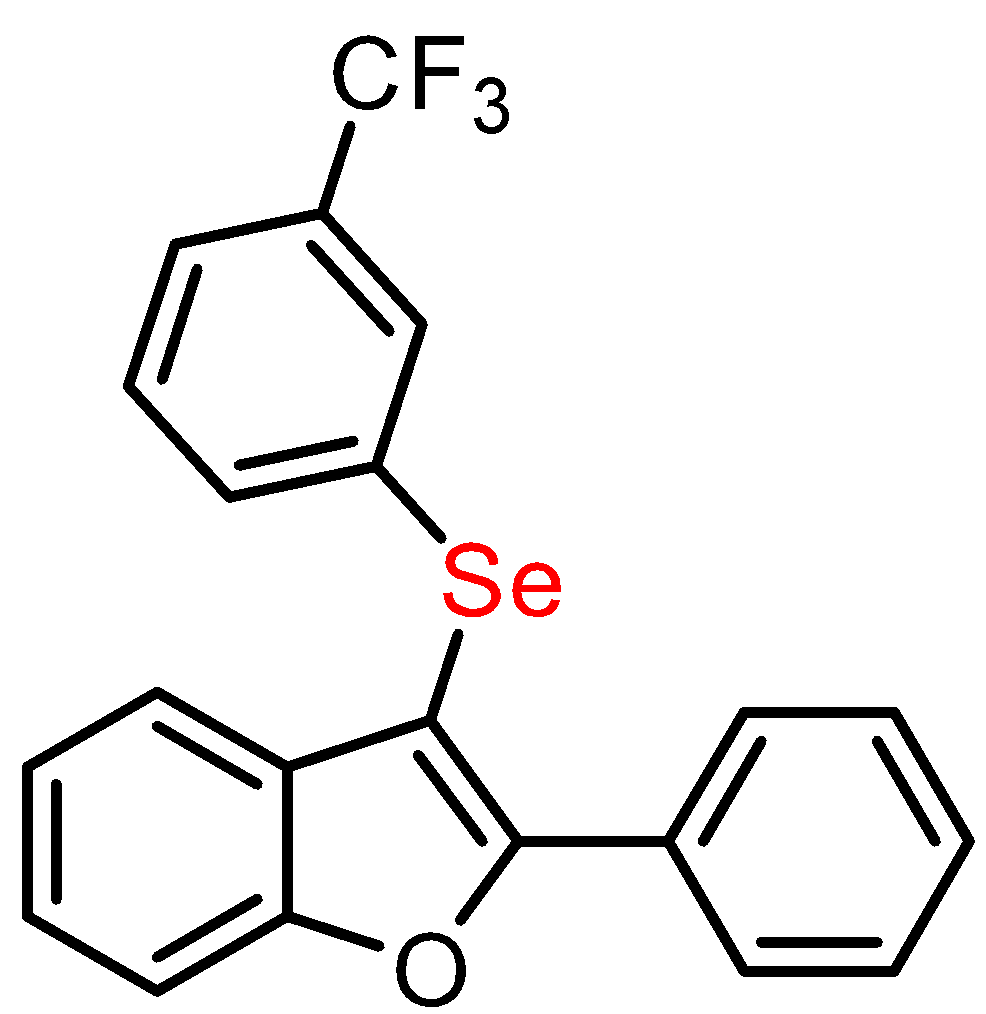


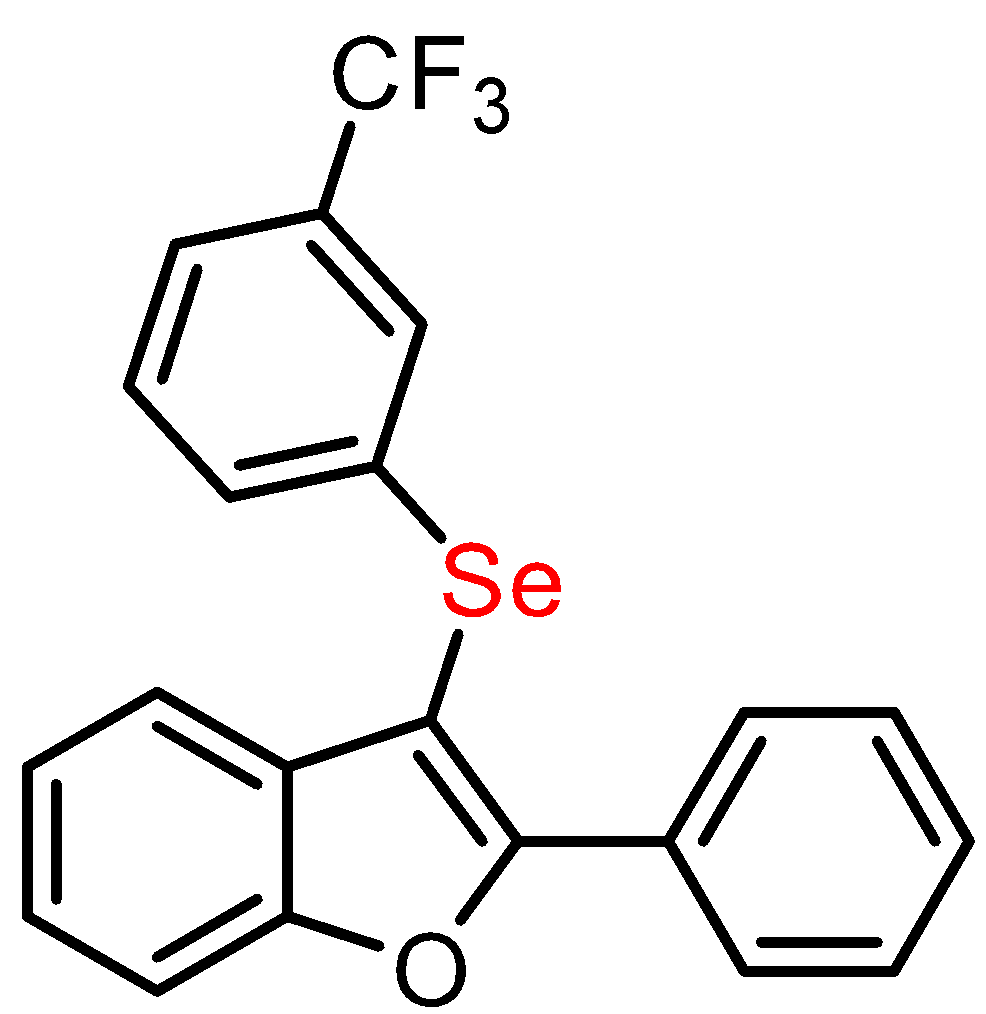


**^1^H NMR (top) and ^13^C NMR (bottom) spectra for 3f in CDCl_3_.**


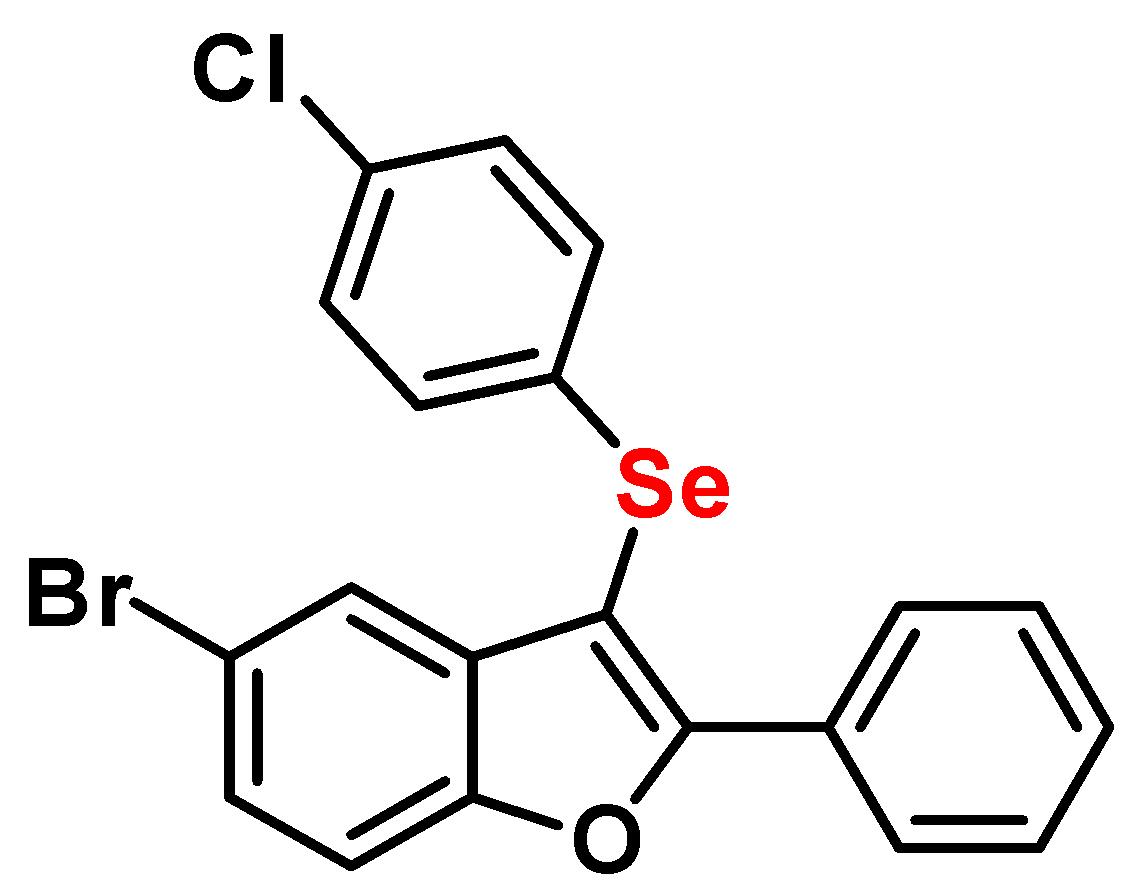


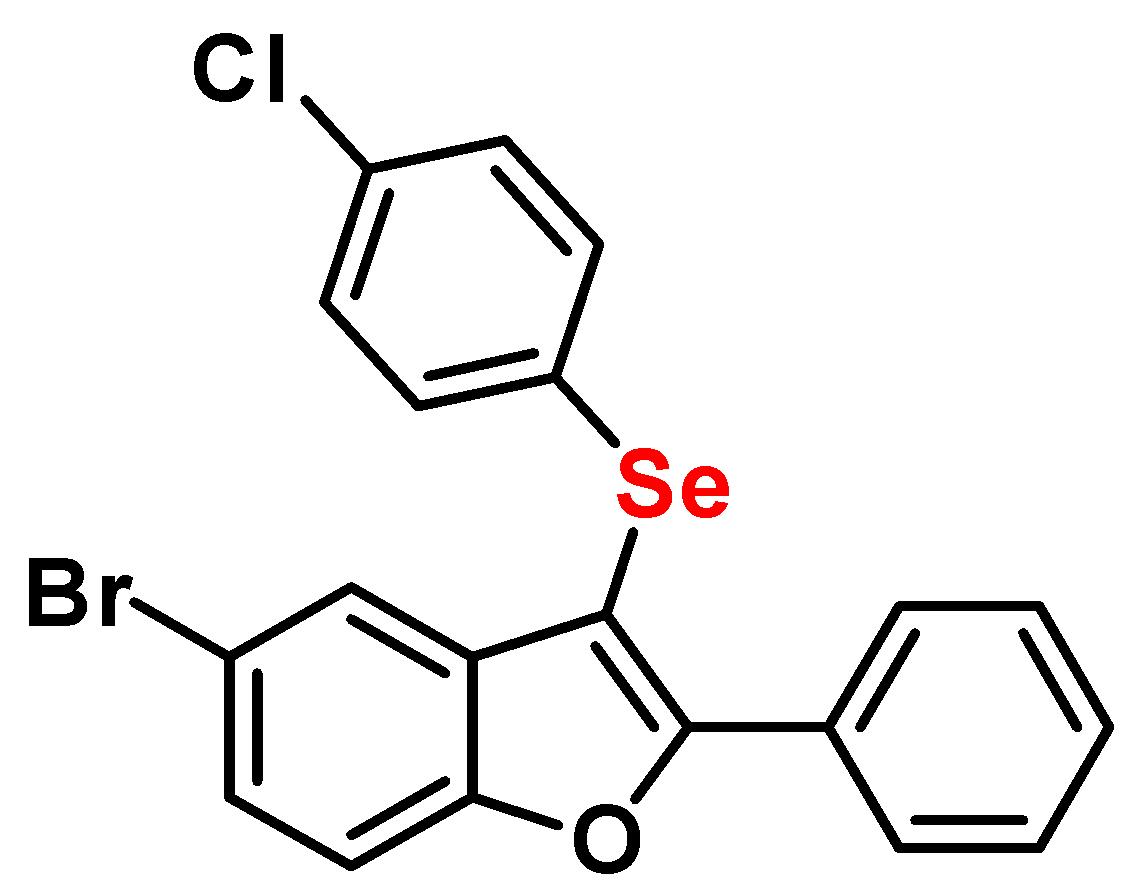


**^1^H NMR (top) and ^13^C NMR (bottom) spectra for Compound 3g in CDCl_3_.**


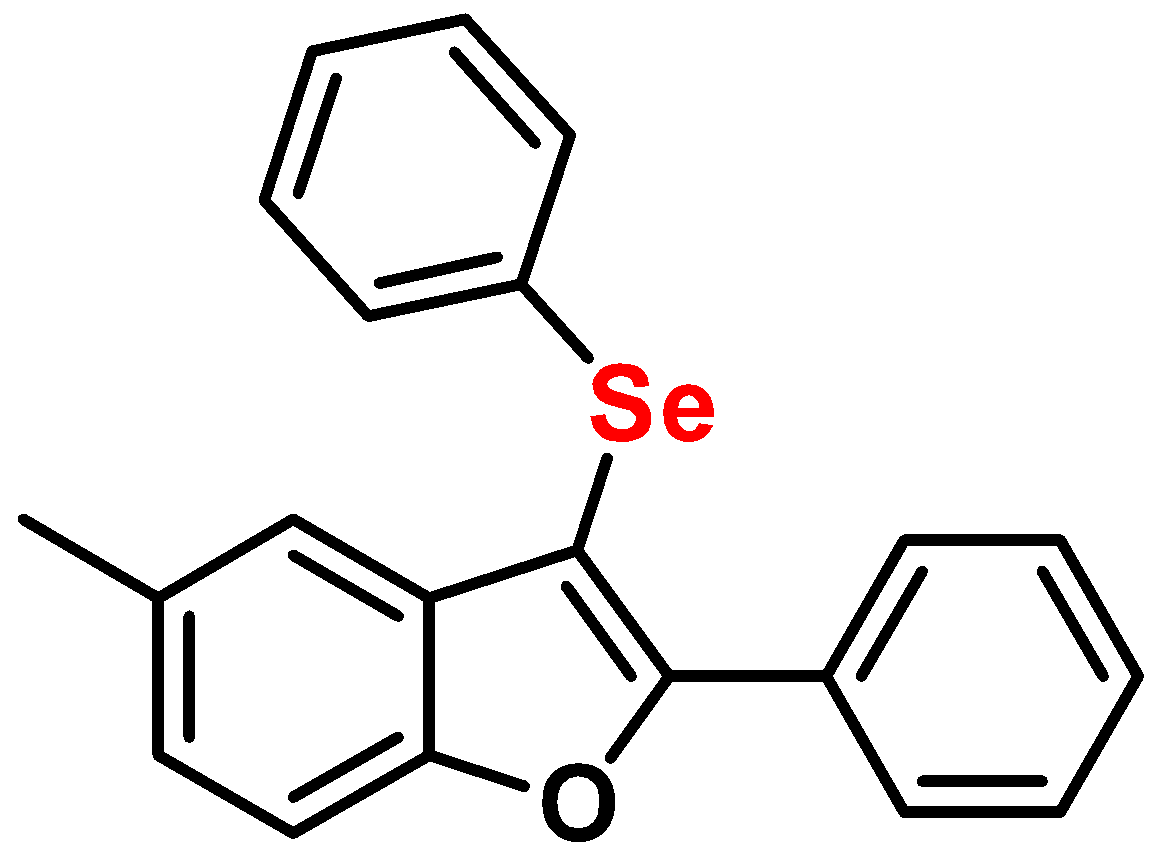


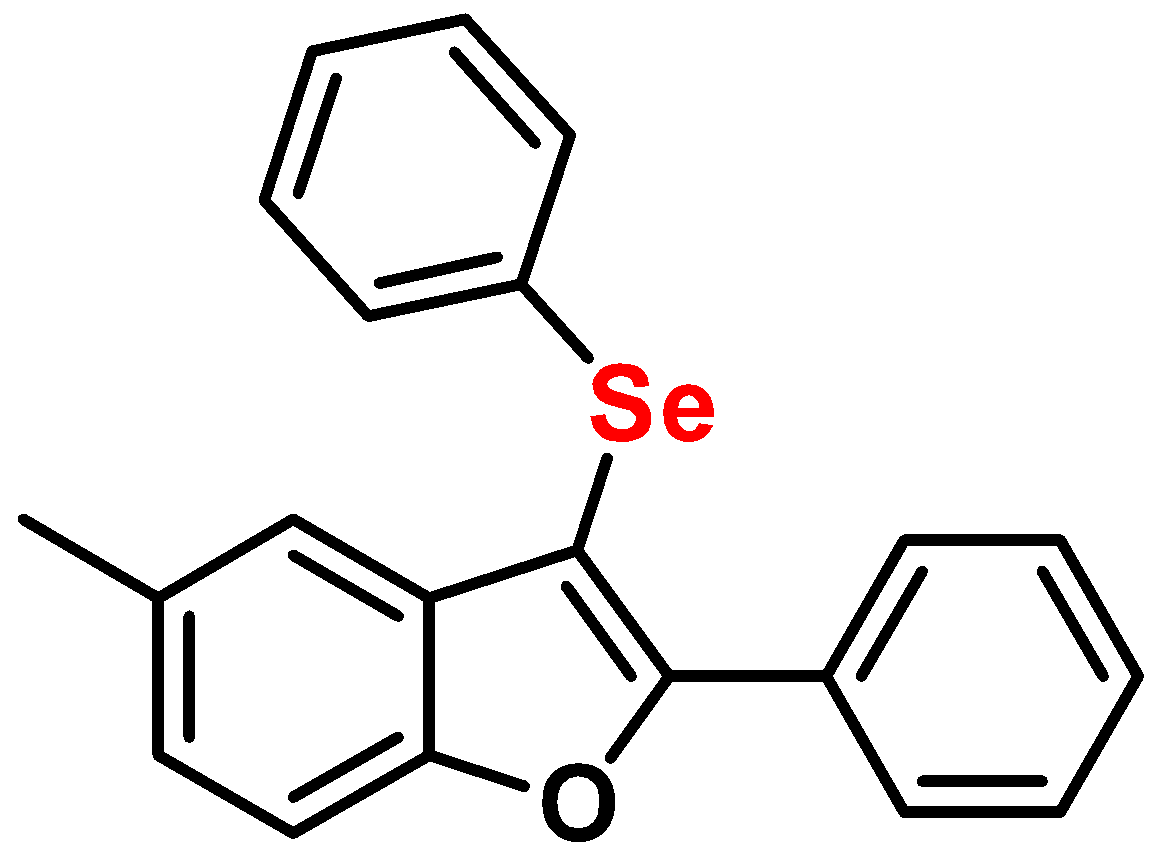


**^1^H NMR (top) and ^13^C NMR (bottom) spectra for Compound 3h in CDCl_3_.**

**^1^H NMR (top) and ^13^C NMR (bottom) spectra for Compound 3i in CDCl_3_.**


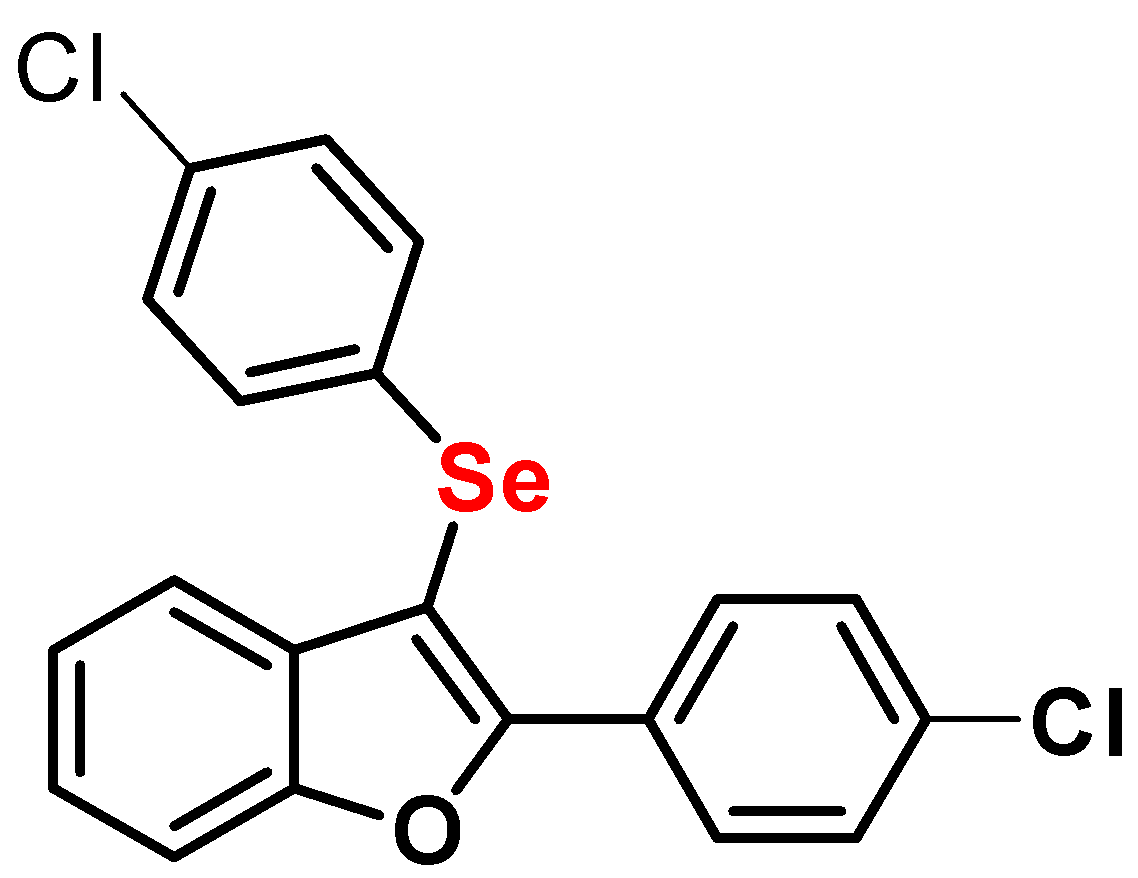

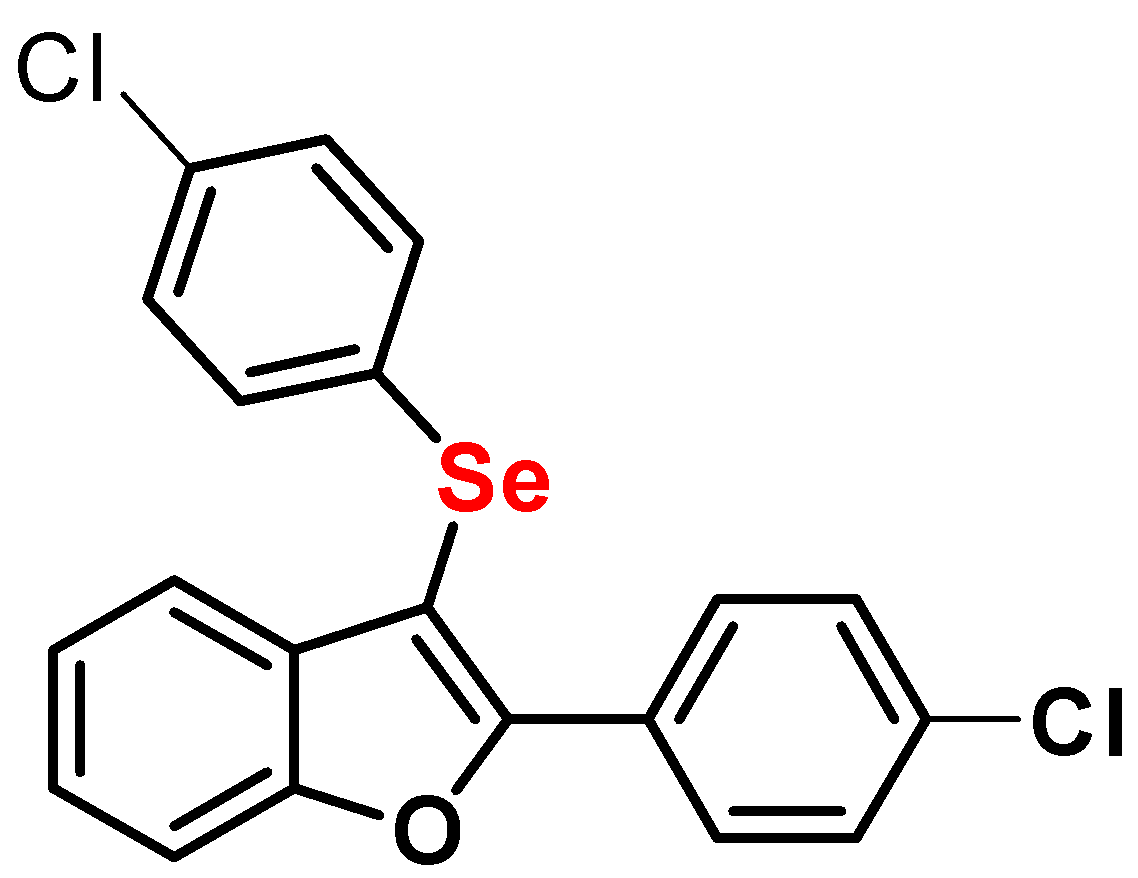

**^1^H NMR (top) and ^13^C NMR (bottom) spectra for Compound 3j in CDCl_3_.**


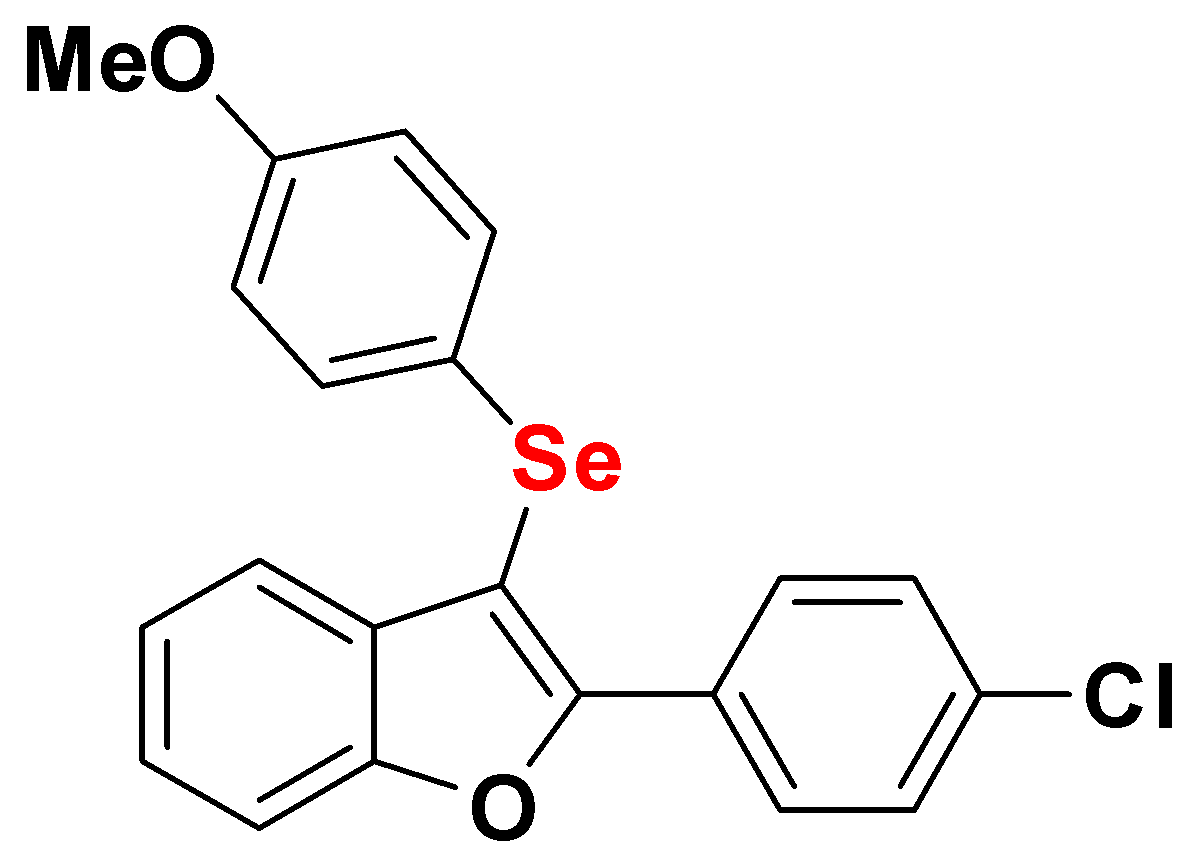

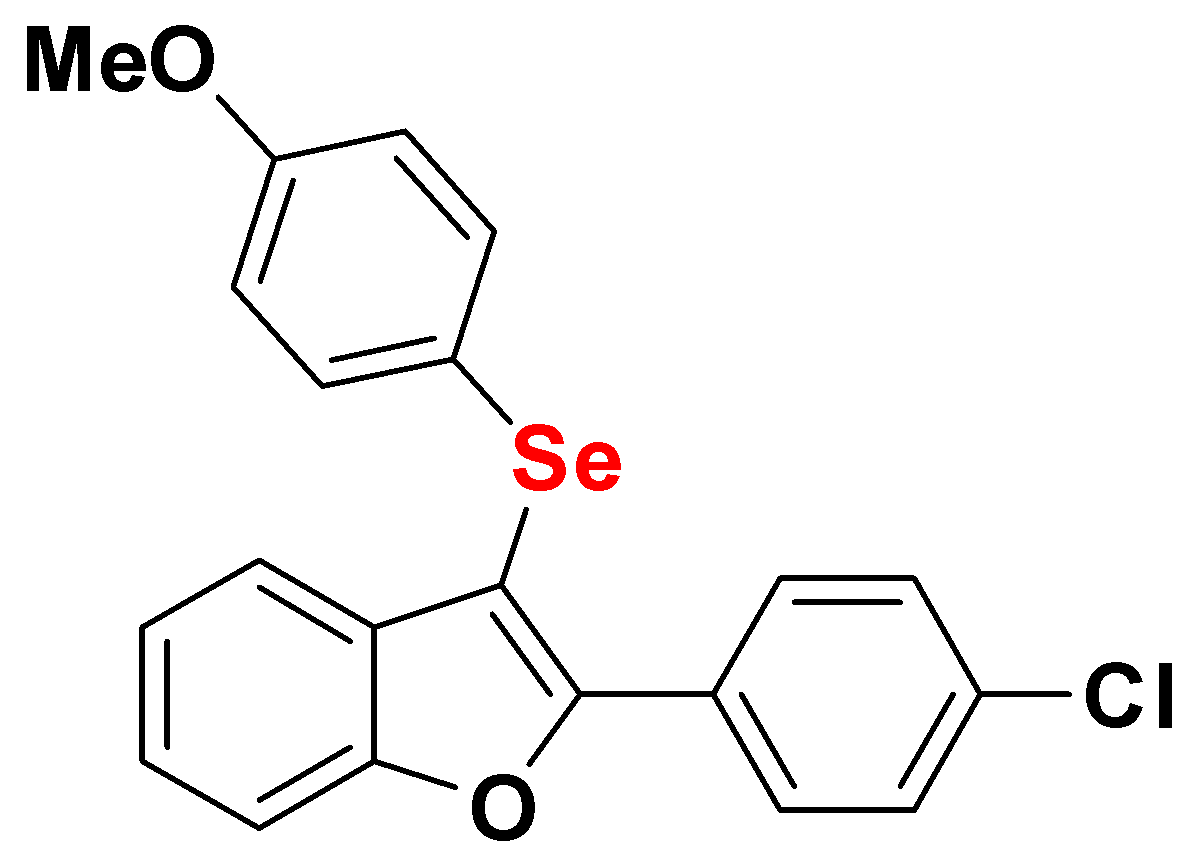

**^1^H NMR (top) and ^13^C NMR (bottom) spectra for Compound 3k in CDCl_3_.**


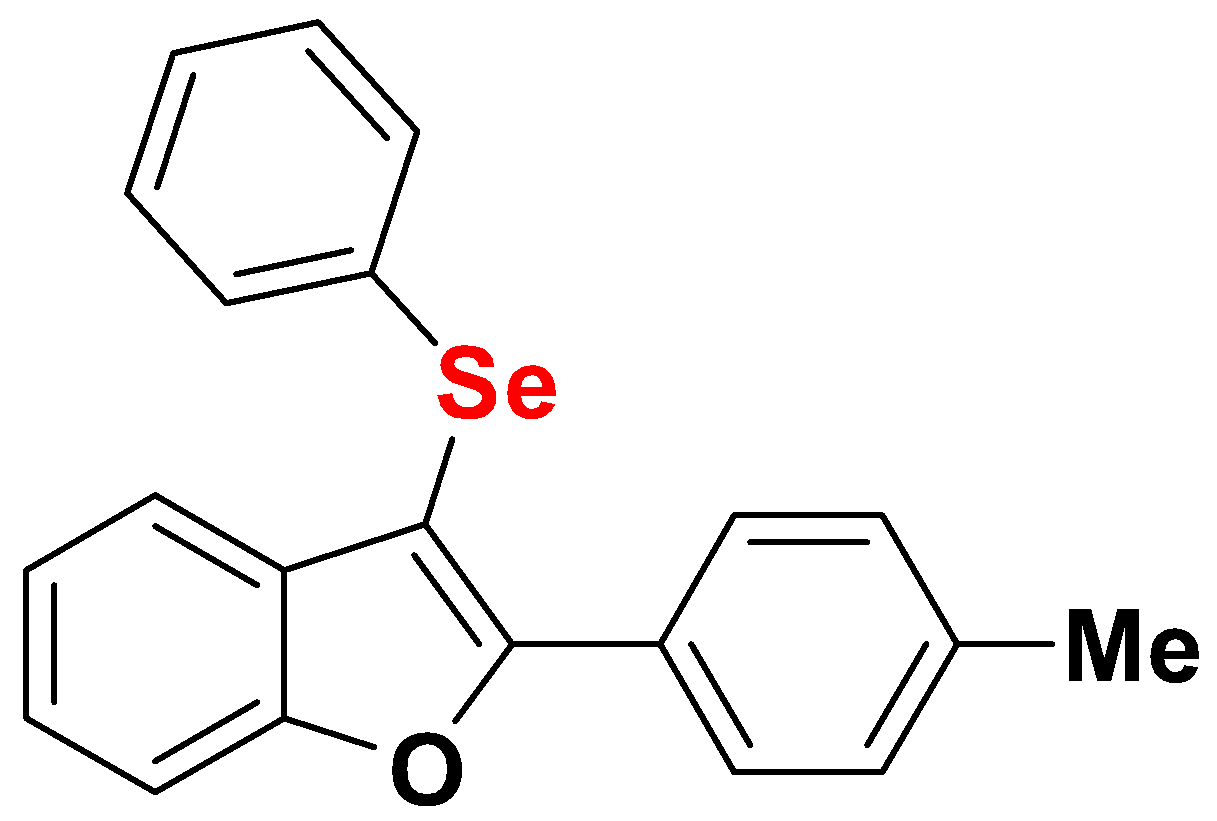

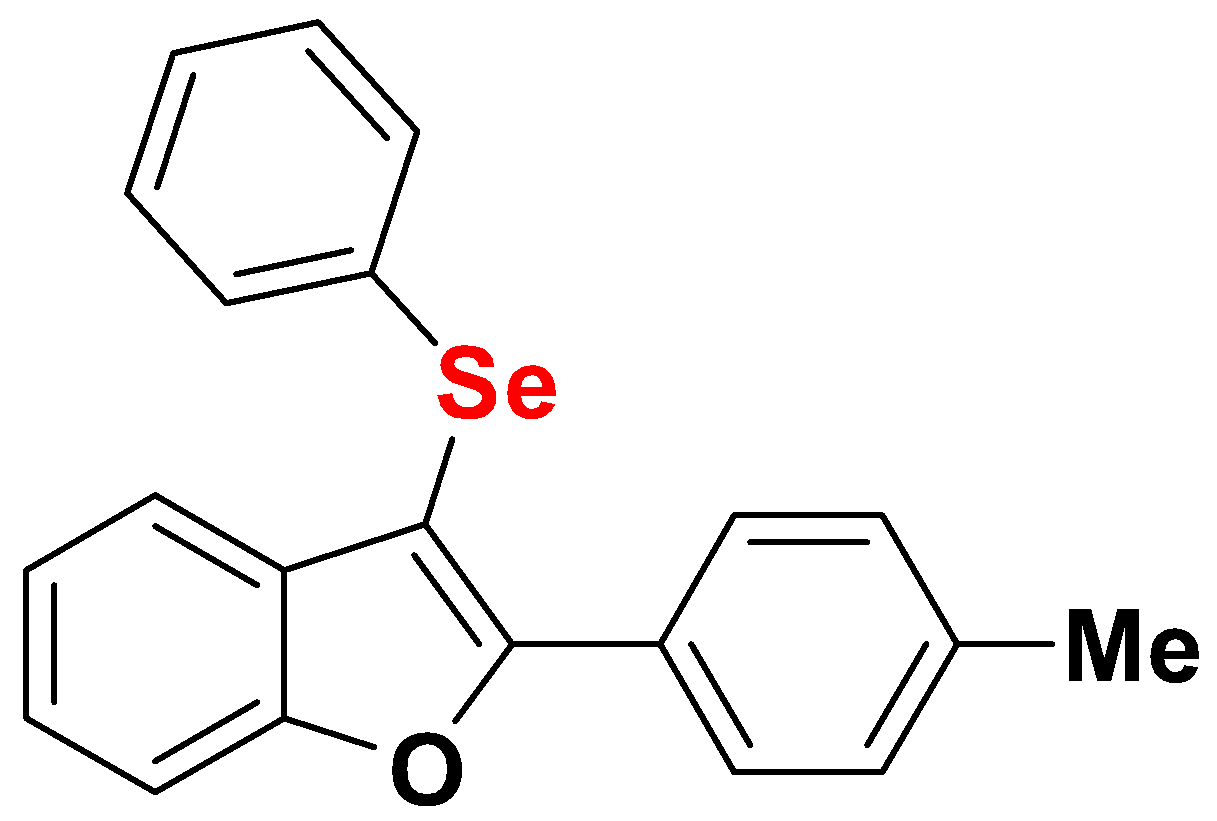

**^1^H NMR (top) and ^13^C NMR (bottom) spectra for Compound 3l in CDCl_3_.**


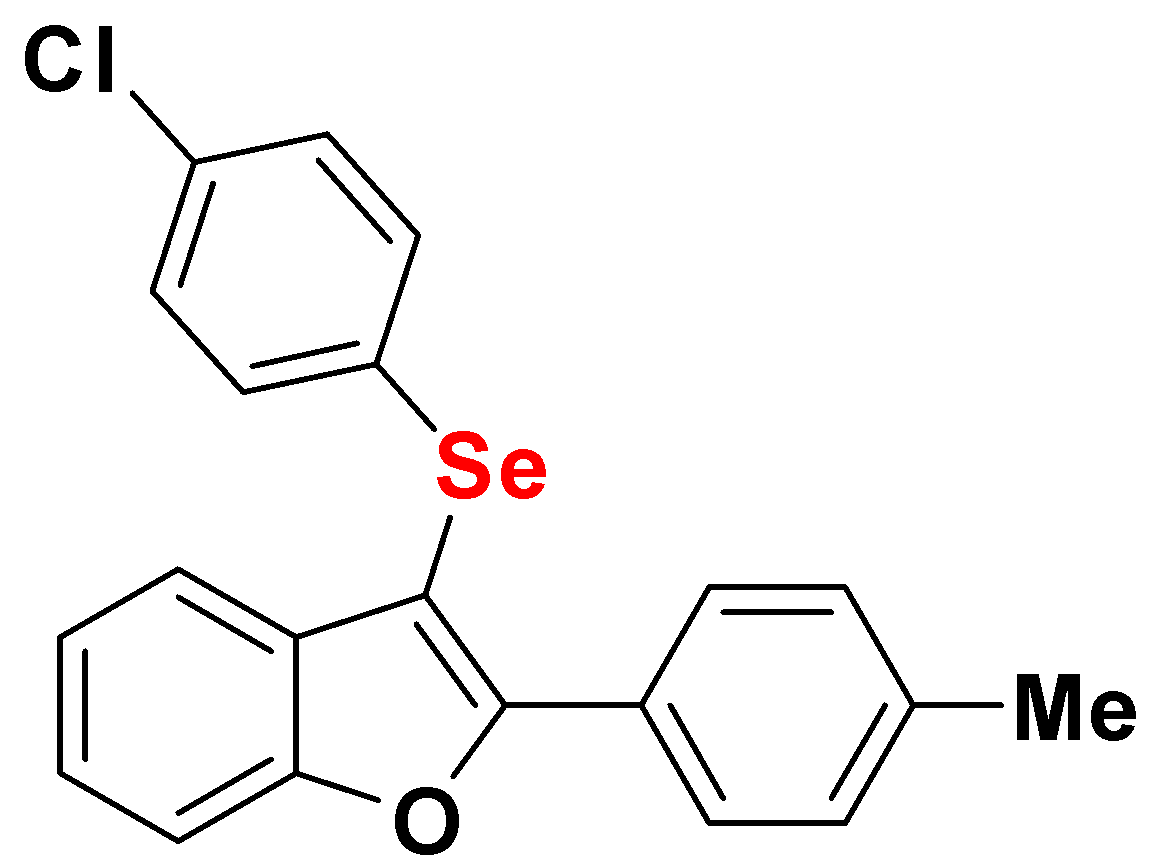


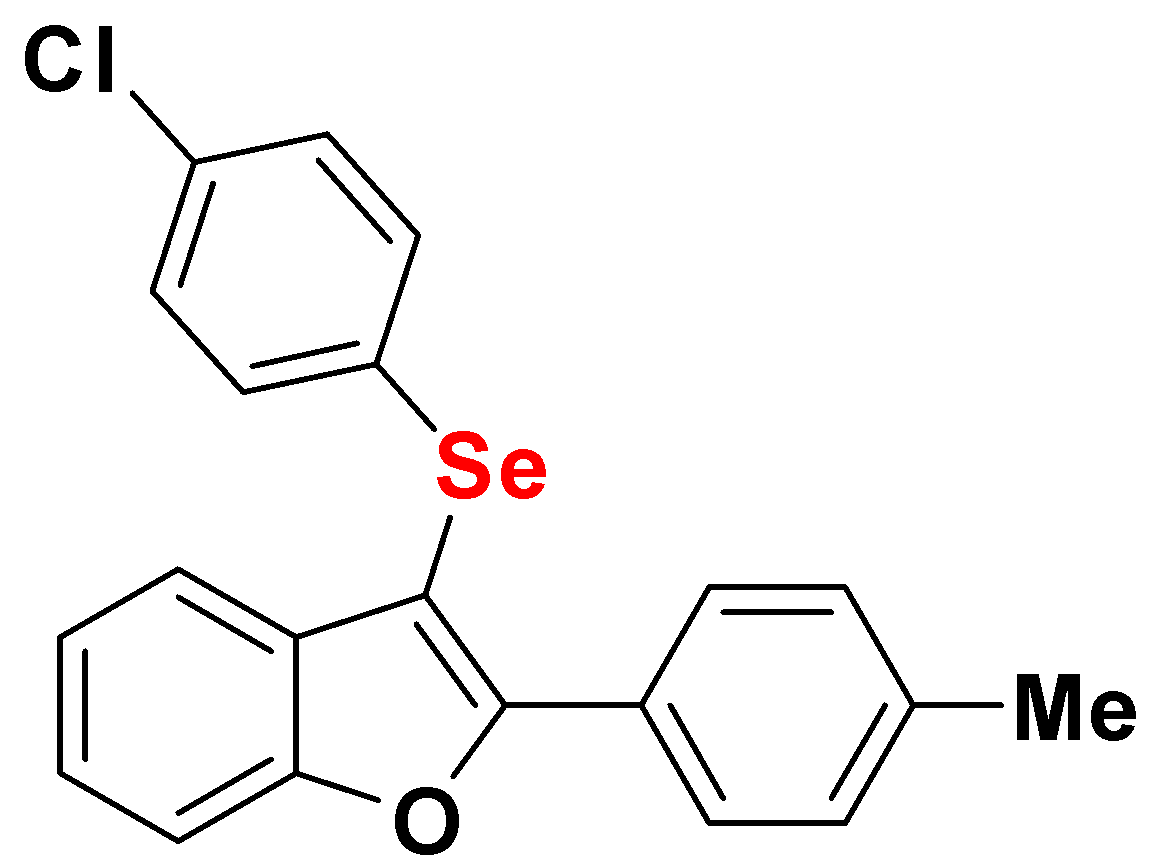


**^1^H NMR (top) and ^13^C NMR (bottom) spectra for Compound 3m in CDCl_3_.**


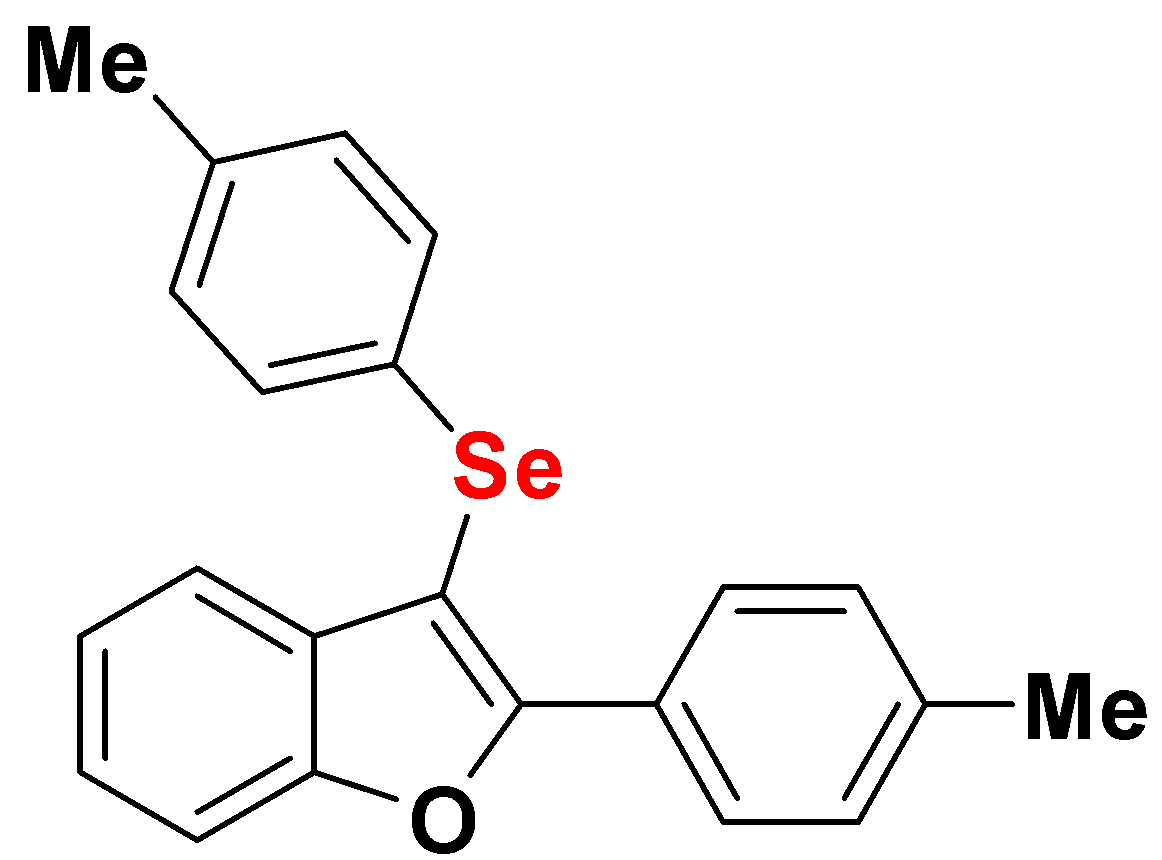

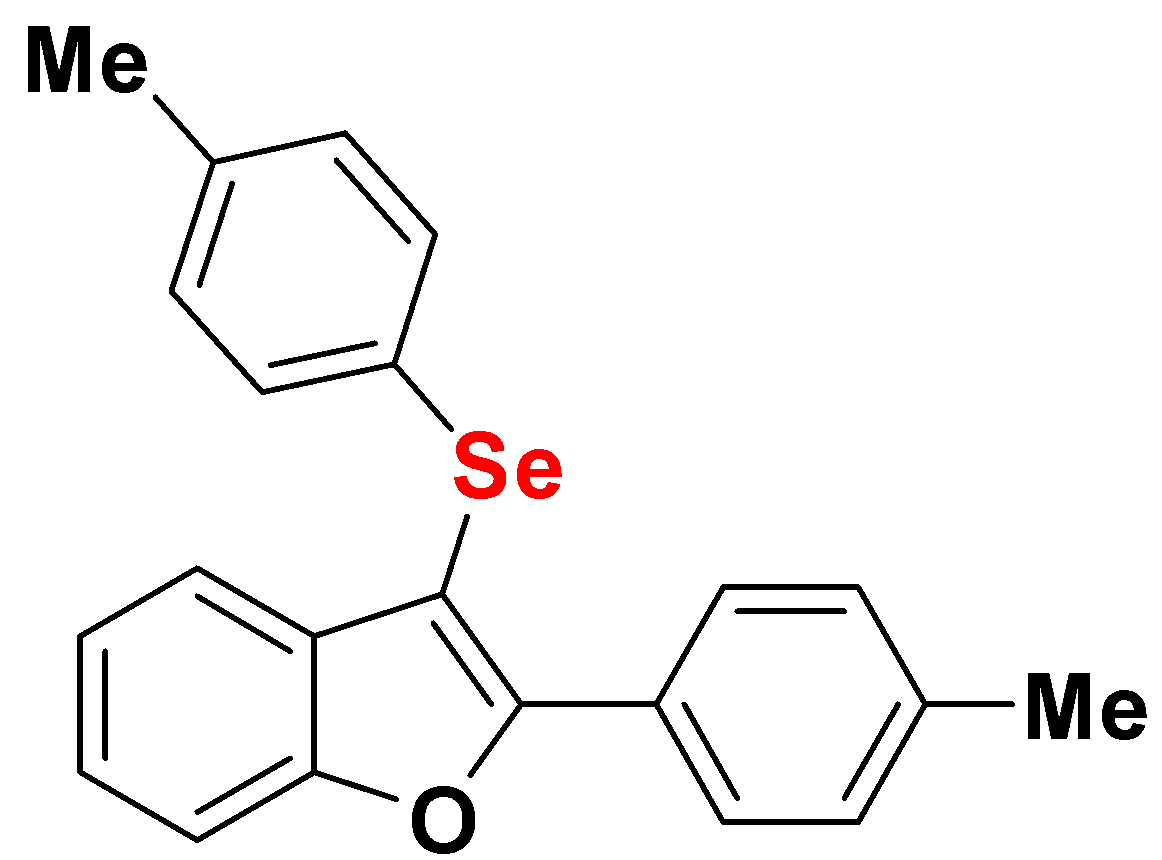


**^1^H NMR (top) and ^13^C NMR (bottom) spectra for Compound 3n in CDCl_3_.**


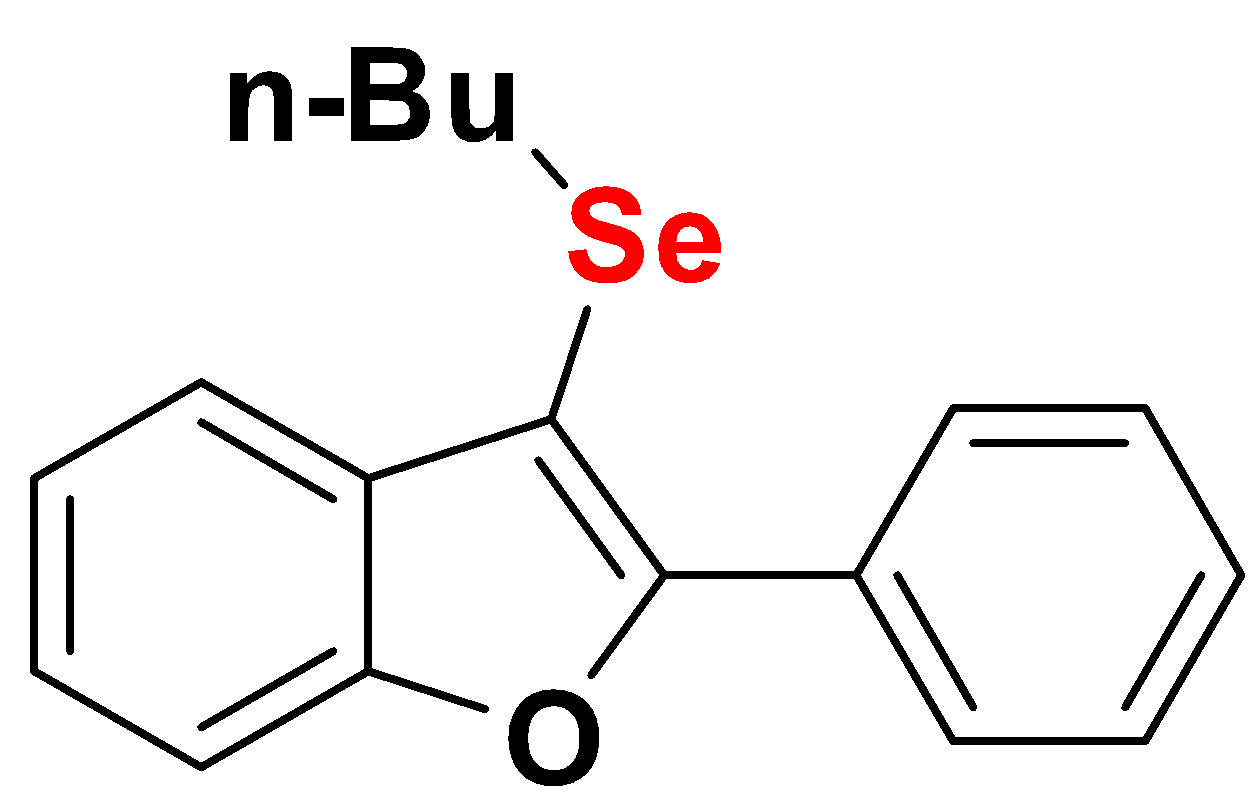

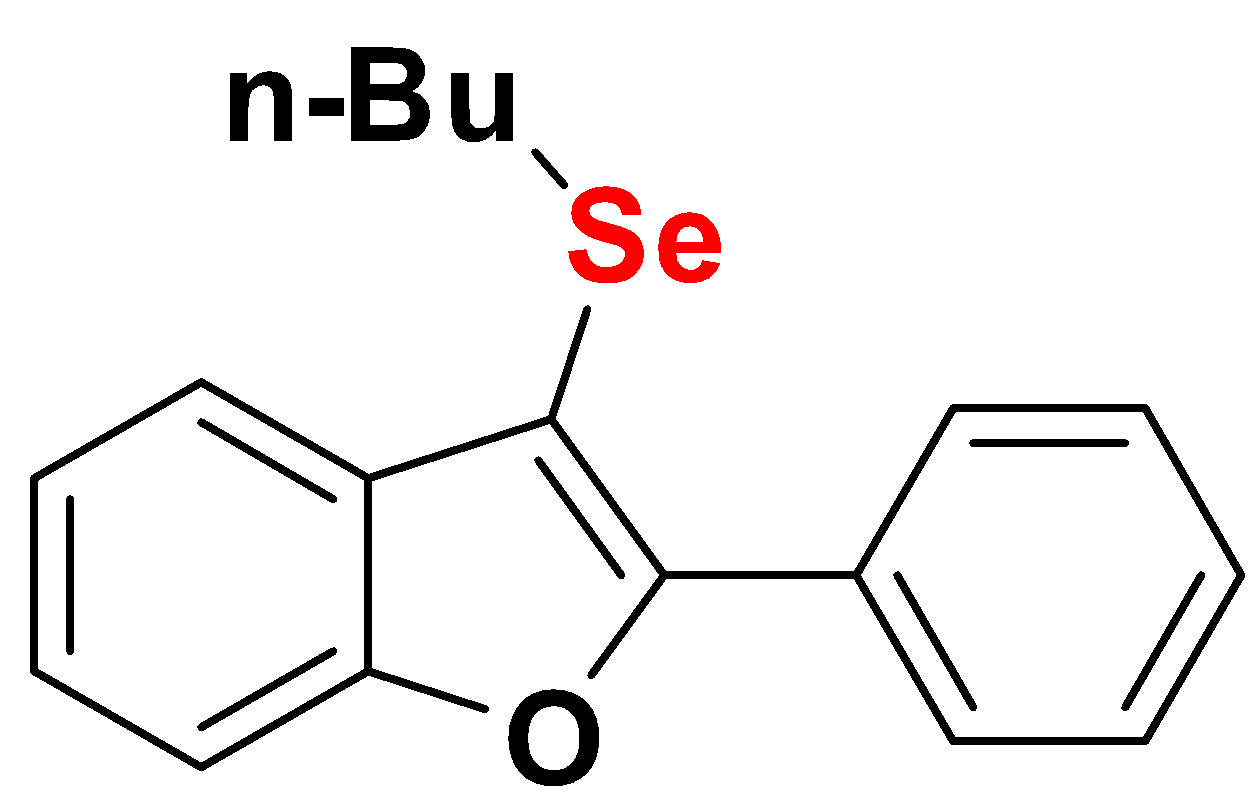


**^1^H NMR (top) and ^13^C NMR (bottom) spectra for 3o in CDCl_3_.**


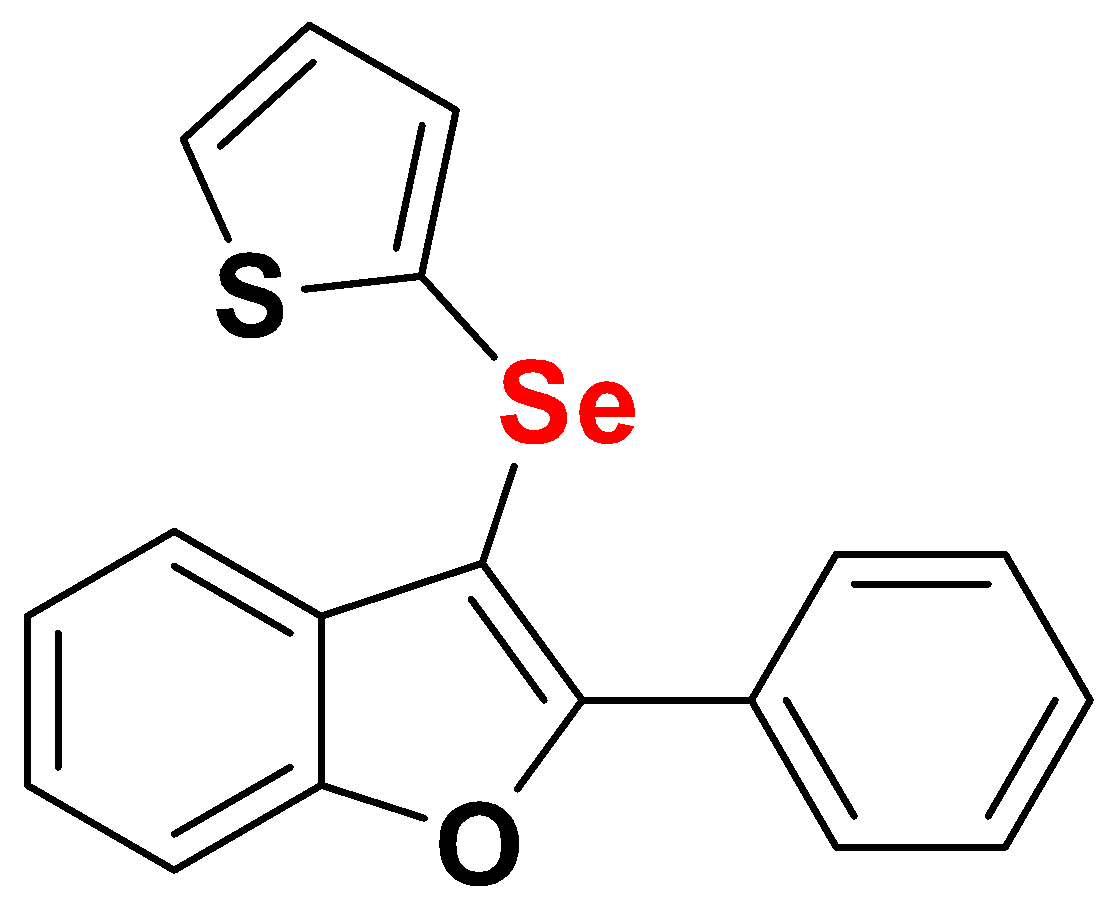

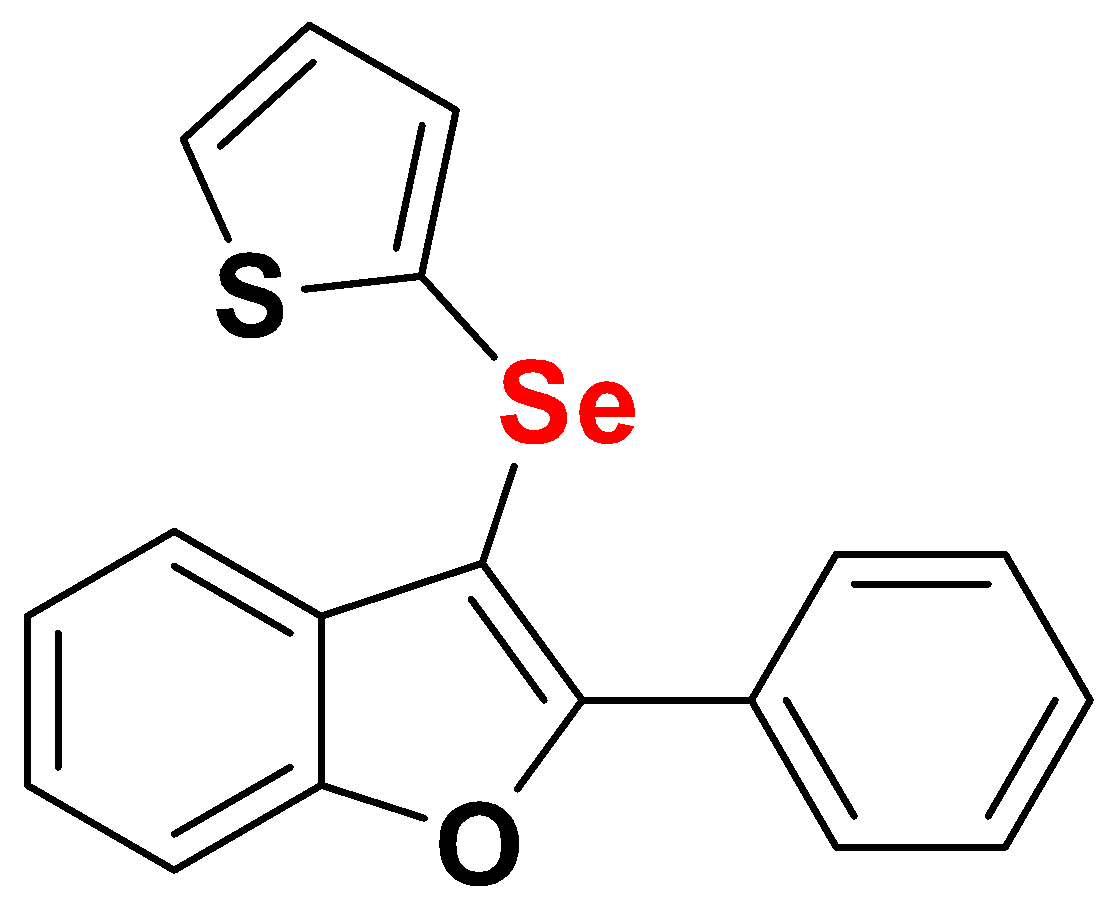

**^1^H NMR (top) and ^13^C NMR (bottom) CDCl_3_ spectra of Compound 3p**

**^1^H NMR (top) and ^13^C NMR (bottom) CDCl_3_ spectra of Compound 3q**

**^1^H NMR (top) and ^13^C NMR (bottom) CDCl_3_ spectra of Compound 4a**

**^1^H NMR (top) and ^13^C NMR (bottom) CDCl_3_ spectra of Compound 4b**

**^1^H NMR (top) and ^13^C NMR (bottom) CDCl_3_ spectra of Compound 4c**

**^1^H NMR (top) and ^13^C NMR (bottom) CDCl_3_ spectra of Compound 4d**

**^1^H NMR (top) and ^13^C NMR (bottom) CDCl_3_ spectra of Compound 1g**

**^1^H NMR (top) and ^13^C NMR (bottom) spectra for Compound 1h in CDCl_3_.**


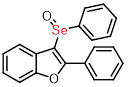


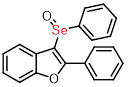


**^1^H NMR (top) and ^13^C NMR (bottom) spectra for Compound 5a in CDCl_3_.**

**8. References**

(1) Lee, E.; Ryu, T.; Park, Y.; Park, S.; Lee, P. H. Tandem Gold-Catalyzed Hydrosilyloxylation-Aldol and -Mannich Reaction with Alkynylaryloxysilanols in 6- Exo Mode. *Adv. Synth. Catal.* **2013**, *355* (8), 1585–1596. https://doi.org/10.1002/adsc.201300244.

(2) Sahani, R. L.; Patil, M. D.; Wagh, S. B.; Liu, R.-S. Catalytic Transformations of Alkynes into Either α-Alkoxy or α-Aryl Enolates: Mannich Reactions by Cooperative Catalysis and Evidence for Nucleophile-Directed Chemoselectivity. *Angew. Chemie Int. Ed.* **2018**, *57* (45), 14878–14882. https://doi.org/10.1002/anie.201806883.

(3) Manarin, F.; Roehrs, J. A.; Gay, R. M.; Brandão, R.; Menezes, P. H.; Nogueira, C. W.; Zeni, G. Electrophilic Cyclization of 2-Chalcogenealkynylanisoles: Versatile Access to 2-Chalcogen-Benzo[ b ]Furans. *J. Org. Chem.* **2009**, *74* (5), 2153–2162. https://doi.org/10.1021/jo802736e.

(4) Oliveira, I. M.; Esteves, H. A.; Darbem, M. P.; Sartorelli, A.; Correra, T. C.; Rodrigues‐Oliveira, A. F.; Pimenta, D. C.; Zukerman‐Schpector, J.; Manarin, F.; Stefani, H. A. Stereo‐ and Regioselective Cu‐Catalyzed Hydroboration of Alkynyl Chalcogenoethers. *ChemCatChem* **2020**, *12* (13), 3545–3552. https://doi.org/10.1002/cctc.202000395.

(5) Diem Ferreira Xavier, M. C.; Andia Sandagorda, E. M.; Santos Neto, J. S.; Schumacher, R. F.; Silva, M. S. Synthesis of 3-Selanylbenzo[: B] Furans Promoted by SelectFluor®. *RSC Adv.* **2020**, *10* (24), 13975–13983. https://doi.org/10.1039/D0RA01907K.

(6) An, C.; Li, C.-Y.; Huang, X.-B.; Gao, W.-X.; Zhou, Y.-B.; Liu, M.-C.; Wu, H.-Y. Selenium Radical Mediated Cascade Cyclization: Concise Synthesis of Selenated Benzofurans (Benzothiophenes). *Org. Lett.* **2019**, *21* (17), 6710–6714. https://doi.org/10.1021/acs.orglett.9b02315.

(7) Gay, R. M.; Manarin, F.; Schneider, C. C.; Barancelli, D. A.; Costa, M. D.; Zeni, G. FeCl3-Diorganyl Dichalcogenides Promoted Cyclization of 2-Alkynylanisoles to 3-Chalcogen Benzo[ b ]Furans. *J. Org. Chem.* **2010**, *75* (16), 5701–5706. https://doi.org/10.1021/jo101126q.

(8) Perin, G.; Soares, L. K.; Hellwig, P. S.; Silva, M. S.; Neto, J. S. S.; Roehrs, J. A.; Barcellos, T.; Lenardão, E. J. Synthesis of 2,3-Bis-Organochalcogenyl-Benzo[ b ]Chalcogenophenes Promoted by Oxone®. *New J. Chem.* **2019**, *43* (16), 6323–6331. https://doi.org/10.1039/C9NJ00526A.
